# An *in vitro* analysis of an innovative standardized phospholipid carrier-based *Melissa officinalis* L*.* extract as a potential neuromodulator for emotional distress and related conditions

**DOI:** 10.3389/fmolb.2024.1359177

**Published:** 2024-03-13

**Authors:** Mehtap Kara, Sumeyye Sahin, Fazle Rabbani, Ezgi Oztas, Gozde Hasbal-Celikok, Ebru Kanımdan, Abdurrahim Kocyigit, Ayesha Kanwal, Ursula Wade, Anastasia Yakunina, Francesco Di Pierro, Amjad Khan

**Affiliations:** ^1^ Department of Pharmaceutical Toxicology, Istanbul University Faculty of Pharmacy, Istanbul, Türkiye; ^2^ Department of Food Engineering, Ordu University, Ordu, Türkiye; ^3^ Department of Psychiatry, Lady Reading Hospital, Peshawar, Pakistan; ^4^ Department of Biochemistry, Istanbul University Faculty of Pharmacy, Istanbul, Türkiye; ^5^ Department of Medical Biochemistry, Bezmialem Vakif University, Istanbul, Türkiye; ^6^ Department of Basic and Clinical Neuroscience, Kings College London, London, United Kingdom; ^7^ Scientific and Research Department, Velleja Research, Milan, Italy; ^8^ Department of Medicine and Surgery, University of Insubria, Varese, Italy; ^9^ Nuffield Division of Clinical Laboratory Sciences, Radcliffe Department of Medicine, University of Oxford, Oxford, United Kingdom; ^10^ Department of Biochemistry, Liaquat University of Medical and Health Sciences, Jamshoro, Pakistan

**Keywords:** Relissa^™^, *Melissa officinalis* L., calming agent, γ-aminobutyrate transaminase (GABA-T), γ-aminobutyrate (GABA), monoamine oxidase A (MAO-A), Phytosome^™^

## Abstract

**Background:** *Melissa officinalis* L. (MO), commonly known as lemon balm, a member of the mint family, is considered a calming herb. In various traditional medicines, it has been utilized to reduce stress and anxiety and promote sleep. A growing body of clinical evidence suggests that MO leaf extract supplementation possesses considerable neuropharmacological properties. However, its possible mechanism of action largely remains unknown.

**Objective:** In the present *in vitro* studies, we comparatively investigated the central nervous system (CNS)-calming and antioxidative stress properties of an innovative standardized phospholipid carrier-based (Phytosome™) MO extract (Relissa™) vs. an unformulated dry MO extract.

**Methods:** The neuropharmacological effect of the extract was studied in the anti-depressant enzymes γ-aminobutyrate transaminase (GABA-T) and monoamine oxidase A (MAO-A) assays and SH-SY5Y cells brain-derived neurotrophic factor (BDNF) expression assay. The neuroprotective effect of the extract against oxidative stress was assessed in SH-SY5Y cell-based (H_2_O_2_-exposed) Total Antioxidant Status (TAS) and Total Reactive Oxygen Species (ROS) assays. The cytotoxic effect of the extract was evaluated using MTT and LDH assays. The extract antioxidant effect was also evaluated in cell-free chemical tests, including TEAC-ABTS, DPPH, Ferric Reducing Antioxidant Power (FRAP), Oxygen Radical Antioxidant Capacity (ORAC), and Hydroxyl Radical Antioxidant Capacity (HORAC) assays.

**Results:** Relissa™ exhibited high GABA-T inhibitory activity, IC_50_ (mg/mL) = 0.064 vs. unformulated dry MO extract, IC_50_ (mg/mL) = 0.27. Similar inhibitory effects were also observed for MAO-A. Relissa™ demonstrated an improved neuroprotective antioxidant effect on SH-SY5Y cells against H_2_O_2_-induced oxidative stress. Compared to unformulated dry MO extract, Relissa™ exerted high protective effect on H_2_O_2_-exposed SH-SY5Y cells, leading to higher cells BDNF expression levels. Moreover, cell-free chemical tests, including TEAC-ABTS, DPPH radical scavenging, FRAP, ORAC, and HORAC assays, validated the improved antioxidant effect of Relissa™ vs. unformulated dry MO extract.

**Conclusion:** The results of the present study support the neuromodulating and neuroprotective properties of Relissa™, and its supplementation may help in the amelioration of emotional distress and related conditions.

## 1 Introduction

Prevalent emotional distress conditions such as depression, anxiety, and chronic stress, in conjunction with sleep disorders, represent widespread mental health challenges that significantly affect an individual’s overall wellbeing. Conventional antidepressant drugs such as tricyclic antidepressants (TCAs), monoamine oxidase inhibitors (MAOIs), and related antidepressants are associated with multiple side effects (Khawam et al., 2006), including discontinuation syndrome, sexual dysfunction, gastrointestinal effects (low tolerability), and low probabilities of remission (Rush, 2007). Similarly, conventional anxiolytic drugs such as selective serotonin reuptake inhibitors (SSRIs) have insufficient overall efficacy in short-term, while long-term treatments have shown adverse effects (Baldwin et al., 2011). Despite the extensive studies on psychiatric disorders, the etiology and pathogenesis of mental health disorders remain unknown. Thus, considering the limitations associated with conventional antidepressants and anxiolytic drugs, it is necessary to identify pharmacological interventions that are both safe and well-tolerated, while also demonstrating effectiveness in the management of emotional distress and associated conditions.

Over recent years, the supplementation of botanical-based pharmacological agents has garnered growing interest in scientists and physicians as potential tools for managing emotional distress and related conditions (Kenda et al., 2022a). Amongst the several botanicals that have been extensively studied for psychopharmacological effects, the leaf extract of *Melissa officinalis* L. (hereafter referred to as MO), commonly known as lemon balm mint, has emerged as a promising agent for calming the central nervous system (CNS), and improving the low mood status. Extensive preclinical and clinical studies have suggested a therapeutic effect of MO extract supplementation as an efficacious and safe therapy to alleviate emotional distress conditions such as low mood, anxiety, and stress and improve sleep disorders (Kennedy et al., 2002; Akhondzadeh et al., 2003; Kennedy et al., 2003; Kennedy et al., 2004; Kennedy et al., 2006; López et al., 2009; Cases et al., 2011; Alijaniha et al., 2015; Arceusz et al., 2015; Soltanpour et al., 2019; Noguchi-Shinohara et al., 2020). Reported evidence suggest that the active chemical constituents of MO extract possess diverse psycho- and neuropharmacological properties, including antidepressant (Awad et al., 2007; Awad et al., 2009; López et al., 2009; Sahin et al., 2016), antioxidative stress (Özkol et al., 2011; Martins et al., 2012; Ghazizadeh et al., 2020; Abd Allah et al., 2022; Abo-Zaid et al., 2023), and anti-inflammatory effects (Baldwin et al., 2011).

An inherent constraint associated with most botanical extracts is their limited solubility and, subsequently, low bioavailability, which often limits their application as a pharmacological agent. In the present work, we carried out a comparative *in vitro* analysis of an innovative standardized phospholipid carrier-based (Phytosome™) MO extract (Relissa™) vs. an unformulated dry MO extract (not mixed with a carrier system) to investigate its antidepressant and antioxidative stress properties. On the basis of previous positive experiences with other botanical extracts (Riva et al., 2019a; Riva et al., 2019b; Rondanelli et al., 2021; Pivari et al., 2022), the Phytosome™ delivery system was applied to the MO extract in order to obtain a stable, food-grade formulation, which can protect the extract constituents from oxidation and optimize the interactions with the microbiome. The Phytosome™ technique constitutes a solid dispersion of botanicals or natural compounds into a 100% food-grade matrix, based on sunflower lecithin (phospholipids) and amphipathic molecules, which acts as an inhibitor of self-aggregation and an effective wetting agent, that also permits a better cellular permeability. The Phytosome™ structure contains the active ingredients of the herbal extract bound to phospholipids. The phospholipid vehicle molecular structure constitutes a water-soluble head and two fat-soluble tails. The interaction between the phospholipid and the phytoconstituents is mainly due to the formation of weak bonds between the polar head of the phospholipid (i.e., phosphate and ammonium groups) and the polar functional groups of the phytoconstituents. Following to these interactions, this phospholipid composition (Phytosome™) can be described as a not-well organized, but as a stable solid dispersion of botanical ingredients in a phospholipidic matrix. The surfactant properties of the phospholipids provides a solid dispersion able to optimize the bioavailability of standardized botanical extracts and improved absorption in the intestinal tract in an intact manner. The unique physicochemical properties and carrier mechanism of the Phytosome™ carrier make it an effective system for the improved delivery of botanical constituents in cells for pharmacological properties (Suryawanshi, 2011; Gandhi et al., 2012). The Phytosome™ carrier system have been shown an efficient carrier system for several botanical agents, including quercetin (Riva et al., 2019b), berberine (Rondanelli et al., 2021), curcumin (Pivari et al., 2022), boswellic acids (Riva et al., 2019a), and other botanical extracts, and has demonstrated improved biological activity for many phytonutrients. Moreover, the Phytosome™ technique is also nanoparticle-free, without any additive or adjuvant, safe, and well-tolerated. The results of the present study strongly support the scientific evidence of the phospholipid carrier-based MO extract as a possible CNS-calming agent that may help in the management of emotional distress conditions such as low mood, anxiety, stress, and related conditions such as sleep disorders.

## 2 Materials and methods

### 2.1 Assays

All assays were performed in triplicates with three freshly prepared samples. Assays were carried out as per the reported protocol or using commercially available assay kits. All assays were performed with a positive control as per the assay kit protocol. The Phytosome™ carrier alone was utilized as a negative control. Unless otherwise mentioned, all chemicals used were purchased, and solutions were prepared in 50 mM aqueous phosphate-buffered saline (PBS).

### 2.2 *Melissa officinalis* L*.* extract

Assays were carried out with an innovative phospholipid (sunflower lecithin) (Phytosome™) carrier-based MO extract (Relissa™) (Bano et al., 2023) and an unformulated dry MO extract (not mixed with a carrier system), both supplied by Indena S.p.A. (Milan, Italy). Relissa™ is an innovative formulation of the MO extract mixed with the Phytosome™ carrier system, which has been standardized and contains 17%–23% hydroxycinnamic acid derivatives and analyzed for its rosmarinic acid content.

### 2.3 SH-SY5Y cell culture

SH-SY5Y neuroblastoma cells (CRL-2266) were purchased from the American Type Culture Collection (ATCC). DMEM/F-12 supplemented with 10% fetal bovine serum (FBS) and penicillin/streptomycin (100 U/100 µg/mL) was used as the cell culture media. The cells were incubated at 37°C in a humidified atmosphere with 5% CO2. The cells were passaged every 3–4th day of the week when they reached confluence. The cells were seeded at densities ranging from 104 to 106 cells, as appropriate for the specific assay (Lopez-Suarez et al., 2022).

### 2.4 Antidepressant enzyme inhibition assays

#### 2.4.1 γ-Aminobutyrate transaminase inhibition assay

γ-Aminobutyrate transaminase (GABA-T) enzyme activity was measured using the reported *in vitro* GABase assay with some modifications (Jacoby, 1962). In this assay, the incubation solution (150 mM potassium pyrophosphate buffer, pH 8.0, and 228.5 mM α-ketoglutarate), 873.0 mM GABase (composed of GABA-T and succinic semialdehyde dehydrogenase (SSA-DH) (Sigma-Aldrich/Merck, United Kingdom) solution, and different concentrations of MO extract solution were added to the wells of a microtiter plate. The GABase solution (1.5–1.7 units/mg) was pipetted into each well. The microtiter plate was then shaken (500 U/min) for 2 min and pre-incubated for 30 min at 37°C. After pre-incubation, 26.1 mM nicotinamide adenine dinucleotide phosphate (NADP) disodium salt was pipetted into each well, and the microtiter plate was shaken for 30 s (500 U/min.). This was followed by the first absorbance measurement (starting value) at 340 nm using a microplate reader (Epoch 2, Agilent BioTek, United States). The microtiter plate was then incubated for 30 min at 37°C, and the absorbance was measured again. The observed change in absorbance due to the reduction in NADP disodium salt was used to calculate the percent GABA-T enzyme activity. To determine the IC_50_ value, the MO extract was tested at various concentrations in the range of 0.02–0.2 mg/mL. The unformulated MO dry extract was used in higher concentrations compared to Relissa™ to achieve 50% enzyme inhibition (0.04–0.4 mg/mL). The absorbance value of the control (water) was set to 100% activity, and activity of each concentration of MO extract was measured relative to the control. Clove (*Syzygium aromaticum*) extract (which has shown strong GABA-T inhibition compared to vigabatrin) was used as a positive GABA-T inhibitor control (Sahin, 2016). All chemicals and MO extract solutions were prepared in water.

#### 2.4.2 Monoamine oxidase A inhibition assay

Monoamine oxidase A (MAO-A) inhibition assay was performed using the MAO-A Inhibitor Screening Assay Kit (Abcam, Cambridge, United Kingdom) (Supplementary Information S1) (He et al., 2023). Briefly, 10 µL MO extract solution and kit-positive control (clorgyline) solution were added to a 96-well plate. Then, 50 µL of MAO-A enzyme solution was added, and the mixture was incubated for 10 min at 25°C. After incubation, kit MAO-A substrate solution was added to the wells, and fluorescence (Ex/Em = 535/587) was measured (Varioskan LUX multimode microplate reader, Thermo Fisher Scientific, Massachusetts, United States) kinetically for 10 min. The percent relative enzyme activity was calculated according to the formula in the protocol.

### 2.5 SH-SY5Y cells Brain-derived neurotrophic factor expression level evaluation assay

SH-SY5Y cells Brain-derived neurotrophic factor (BDNF) expression levels assay was performed using an Elisa Kit (Abcam, Cambridge, United Kingdom) (Supplementary Information S2). In this assay, 1 × 10^6^ SH-SY5Y cells were seeded into T25 culture flasks and incubated overnight for attachment. Cells were then co-treated with 150 µM H_2_O_2_ (to induce oxidative stress) (Law et al., 2014; Nieto et al., 2018; Morán-Santibañez et al., 2019; Deligoz and Cumaoglu, 2023) and appropriate concentrations of MO extract solution and incubated for 24 h. After the incubation period, the cell culture supernatant was prepared, and BDNF expression levels were evaluated (as per the kit protocol). Briefly, kit standards (positive control) and the cell culture supernatant were added onto a 96-well plate, 50 µL of antibody cocktail was added, and the mixture was incubated at room temperature for 1 h on a shaker with 400 rpm. Following incubation and the washing period, 100 µL of the kit TMB development reagent was added to each well, followed by the addition of 100 µL of the kit stop solution. Upon change in the assay mixture color from blue to yellow by the stop solution, the color intensity was measured at 450 nm (BioTek, Epoch, Vermont, United States) (Wauquier et al., 2022).

### 2.6 SH-SY5Y cells-based antioxidant activity assays

#### 2.6.1 Total Aantioxidant Status activity assay

The Total Antioxidant Status (TAS) assay was performed using a Total Antioxidant Status Elisa Kit (Elabscience Technology Laboratory, United States) (Supplementary Information S3). Briefly, SH-SY5Y neuroblastoma cells (CRL-2266) were treated with varying concentrations of MO extract, and absorbance was measured at 660 nm using a microplate reader (BioTek, Epoch, Vermont, United States). The TAS levels were calculated relative to the absorbance curve of the kit standard solution (Erel, 2004).

#### 2.6.2 Reactive Oxygen Species generation assay

Reactive Oxygen Species (ROS) assay was performed as previously reported (Kara et al., 2020). The total ROS production in SH-SY5Y cells was evaluated with H_2_DCF-DA dye using a flow cytometer. Briefly, 5 × 10^5^ SH-SY5Y cells were seeded into a 6-well plate and incubated overnight for the attachment. Cells were then co-treated with 150 µM H_2_O_2_ (to induce oxidative stress) and MO extract solution. After 24 h of incubation, cells were washed with PBS and then incubated with 20 µM H_2_DCF-DA at 37°C for 30 min. After incubation, cells were detached and washed again with PBS. Following washing, cells were resuspended with 1% BSA in 150 µL PBS. The fluorescence intensity of 1 × 10^4^ cells was measured using an ACEA NovoCyte flow cytometer (San Diego, California, United States), and the results were expressed as percent median fluorescence intensity (MFI%) (Kara et al., 2020).

### 2.7 Cytotoxicity assay

#### 2.7.1 MTT assay

The metabolic activity of SH-SY5Y cells was evaluated using the MTT [3-(4,5-dimethylthiazol-2-yl)-2,5 diphenyl tetrazolium bromide] assay, as previously reported (Kara et al., 2022). Briefly, after 24 h of incubation for the attachment of cells, cells were treated with various concentrations of the MO extract solution and were incubated again for 24 h. The MTT solution (5 mg/mL) was then added to each well and further incubated for 3 h at 37°C, and absorbances were measured using a spectrophotometer (BioTek, Epoch, Vermont, United States) at 590 nm.

In another MTT assay, SH-SY5Y cells were co-treated with a mixture of 150 µM H_2_O_2_ (to induce oxidative stress) (Law et al., 2014; Nieto et al., 2018; Morán-Santibañez et al., 2019; Deligoz and Cumaoglu, 2023) and the protective effect of MO extract on SH-SY5Y cells viability against H_2_O_2_-induced oxidative stress was assessed (Kara et al., 2022).

#### 2.7.2 Lactate dehydrogenase release assay

The assay was performed using a lactate dehydrogenase (LDH) cytotoxicity assay kit (Roche) (Supplementary Information S4). Briefly, SH-SY5Y cells were seeded onto a 96-well plate (1 × 10^4^ cells/well) and grown for 24 h. Cells were then co-treated with a mixture of 150 µM H_2_O_2_ (to induce oxidative stress) and MO extract solution (Law et al., 2014; Nieto et al., 2018; Deligoz and Cumaoglu, 2023) and incubated for 24 h, and the LDH released due to membrane damage was evaluated according to the manufacturer’s protocol. 1% Triton X-100 was used as a positive control. Optical density was measured at 495 nm using a microplate reader. The percent LDH released was calculated using the equation of a linear regression curve (Sevim et al., 2020).

### 2.8 Cell-free system antioxidant activity assays

#### 2.8.1 Trolox Equivalent Antioxidant Capacity-ABTS assay

The Trolox Equivalent Antioxidant Capacity (TEAC) assay was performed using a commercial kit (Cell Biolabs, San Diego, United States) (Supplementary Information S5). Briefly, 25 µL of the kit standard (Trolox) and MO extract solution were added to a 96-well plate, followed by the addition of 150 µL of [2,2′-azinobis (ethylbenzothiazoline 6-sulfonate)] (ABTS) to each MO extract sample and standard well. After 5 min of incubation, solution absorbance was measured at 405 nm using a plate reader spectrophotometer (BioTek, Epoch, Vermont, United States) (Romulo, 2020).

#### 2.8.2 2,2-Diphenyl-1-picrylhydrazyl) radical scavenging/antioxidant activity assay

The 2,2-diphenyl-1-picrylhydrazyl (DPPH) radical scavenging activity assay was carried out according to the reported modified method of Brand-Williams et al. (1995). Briefly, 10 μL MO extract solution was mixed with 240 μL of 0.1 mM DPPH radical working solution. The mixture was incubated in a dark room at room temperature for 30 min. The decrease in assay solution absorbance was measured at 517 nm against a methanol blank solution. Quercetin was used as a standard antioxidant (positive control), while the assay buffer solution was used as a negative control. DPPH radical scavenging activity (%) was calculated using the below equation (Brand-Williams et al., 1995; Hasbal et al., 2015):

DPPH radical scavenging activity (%) = 1- [(Absorbance of the extract at 517 nm)/(Absorbance of the control at 517 nm)] ×100.

#### 2.8.3 Ferric Reducing Antioxidant Power assay

The assay was performed using a Ferric Reducing Antioxidant Power (FRAP) assay kit (BQC, Asturias, Spain) (Supplementary Information S6). Briefly, 10 µL of each Fe (II) standard and MO extract solution were added to a 96-well plate, followed by the addition of 220 µL of the reaction reagent to each of the extract and standard wells. After 5 min of incubation, assay solution absorbance was measured at 593 nm using a plate reader spectrophotometer (BioTek, Epoch, Vermont, United States).

#### 2.8.4 Oxygen Radical Antioxidant Capacity assay

The assay was performed using an Oxygen Radical Antioxidant Capacity (ORAC) assay kit (BQC, Asturias, Spain) (Supplementary Information S7). Briefly, 15 µL of the kit standard and MO extract solution were added to a 96-well plate, followed by the addition of 90 µL of kit solution B reaction reagent to both the extract and standard wells. After 15 min of incubation at 37°C, 45 µL of kit solution C reaction reagent was added to the standards and extract sample wells. Assay solution absorbance was measured at Ext/Em 485 nm/528–538 nm using a fluorescent plate reader (FLx800, BioTek Instruments Inc., Winooski, VT, United States).

#### 2.8.5 Hydroxyl Radical Antioxidant Capacity assay

The assay was performed using a Hydroxyl Radical Antioxidant Capacity (HORAC) assay kit (Zen-Bio, Durham, United States) (Supplementary Information S8). Briefly, 20 µL of the kit standard and MO extract solution were added to a 96-well plate and incubated for 10 min, followed by the addition of 140 µL of fluorescent working solution and then 20 µL of the kit radical initiator solution to the wells. Assay solution absorbance was measured using a fluorescent plate reader (FLx800, BioTek Instruments Inc., Winooski, VT, United States).

## 3 Statistical analysis

Data were expressed as the mean ± standard deviation (SD). Statistical analyses were performed with one-way ANOVA and *post hoc* Dunnett’s *t*-test using SPSS v.20 (IBM SPSS Inc., New York, NY, United States). *p*-value <0.05 was considered as statistically significant difference. For MO extract GABase IC_50_ values with a 95% confidence interval (±95% CI), a linear regression was performed using OriginPro^®^ (OriginPro^®^ 2023b, OriginLab Corporation, Northampton, MA 01060, United States).

## 4 Results

### 4.1 Antidepressant enzyme assays

#### 4.1.1 GABA-T inhibitory activity assay

**FIGURE 1:**
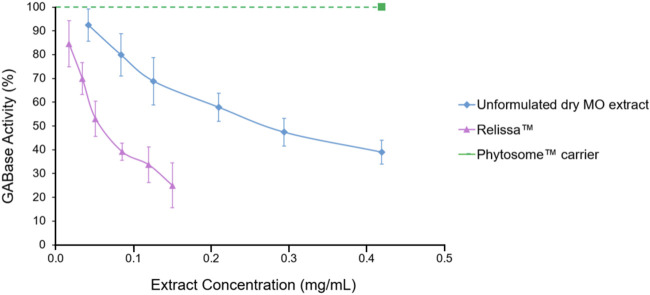
*Melissa Officinalis* L*.* (MO) extract GABA-T inhibition assay. Error bars represent standard deviation from the mean of three separate measurements.

The Relissa™ extract was used at low-to-high concentrations of 0.02, 0.03, 0.05, 0.09, 0.12, and 0.15 mg/mL in the GABase assay to investigate its inhibitory effect on GABA metabolizing enzymes (Figure 1). The unformulated dry MO extract was used at higher concentrations (0.04–0.42 mg/mL) as compared to Relissa™ extract to achieve GABA-T inhibitory effect (Figure 1). Relissa™ demonstrated a strong GABA-T inhibitory effect with an IC_50_ value of 0.064 mg/mL, while the unformulated dry MO extract exhibited reduced potency (IC_50_ 0.27 mg/mL). The linear regression plots of the effect of Relissa™ and the unformulated dry MO extract on GABase activity are presented in Supplementary Information S9. The Phytosome™ carrier alone did not exhibit any noticeable GABA-T inhibitory activity (Figure 1).

#### 4.1.2 MAO-A inhibition assay

**FIGURE 2:**
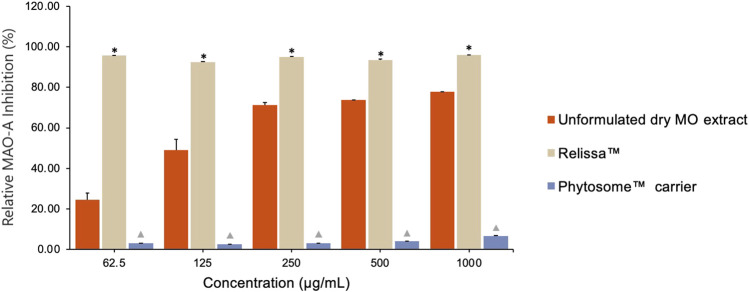
Qualitative assay showing MO extract MAO-A inhibitory effect. Error bars represent standard deviation from the mean of three separate measurements. *p < 0.05, Relissa™ compared to unformulated dry MO extract; 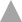 *p* < 0.05, Phytosome™ compared to Relissa™ and unformulated dry MO extract.

The Relissa™ MAO-A inhibitory effect was statistically improved even at low concentrations as compared to unformulated dry MO extract (*p < 0.05) (Figure 2). However, the MAO-A inhibitory effect of unformulated dry MO extract increased in a concentration-dependent manner. The Phytosome™ carrier alone did not demonstrate any noticeable MAO-A inhibitory activity (*p* < 0.05).

### 4.2 SH-SY5Y cells BDNF expression level evaluation assay

**FIGURE 3:**
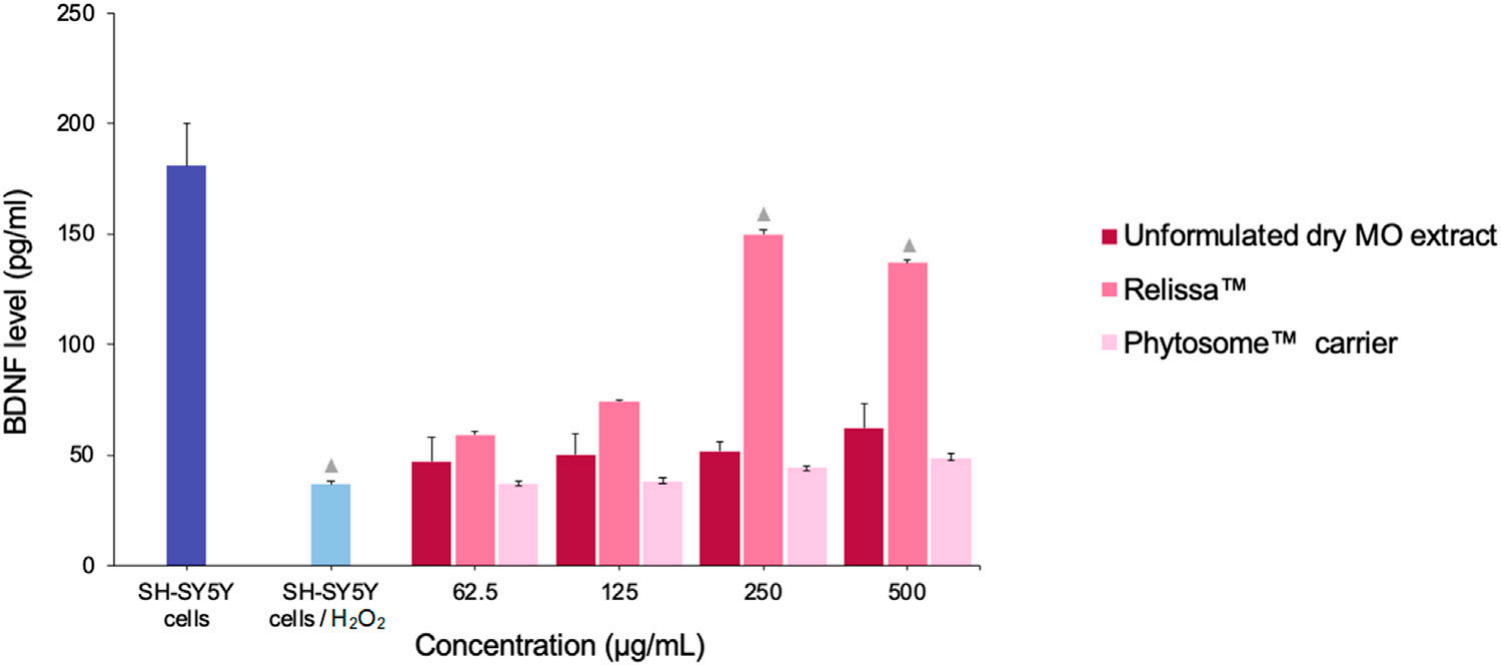
Treatment effect of MO extracts on SH-SY5Y cells (exposed to H_2_O_2_-induced oxidative stress) BDNF expression levels. 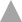 *p* < 0.05, compared to SH-SY5Y cells/H_2_O_2_. Error bars represent standard deviation from the mean of three separate measurements.

The SH-SY5Y cells BDNF expression levels decreased significantly upon exposure to H_2_O_2_ due to oxidative stress damage (Figure 3). In the presence of Relissa™, in a dose-dependent manner, through the protective effect of SH-SY5Y cells, BDNF expression levels increased significantly, while the unformulated dry MO extract, as well as the Phytosome™ carrier alone, did not lead to an increase in the SH-SY5Y cells BDNF expression levels (*p* < 0.05).

### 4.3 SH-SY5Y cells-based antioxidant activity assays

#### 4.3.1 TAS and total ROS generation assays

**FIGURE 4:**
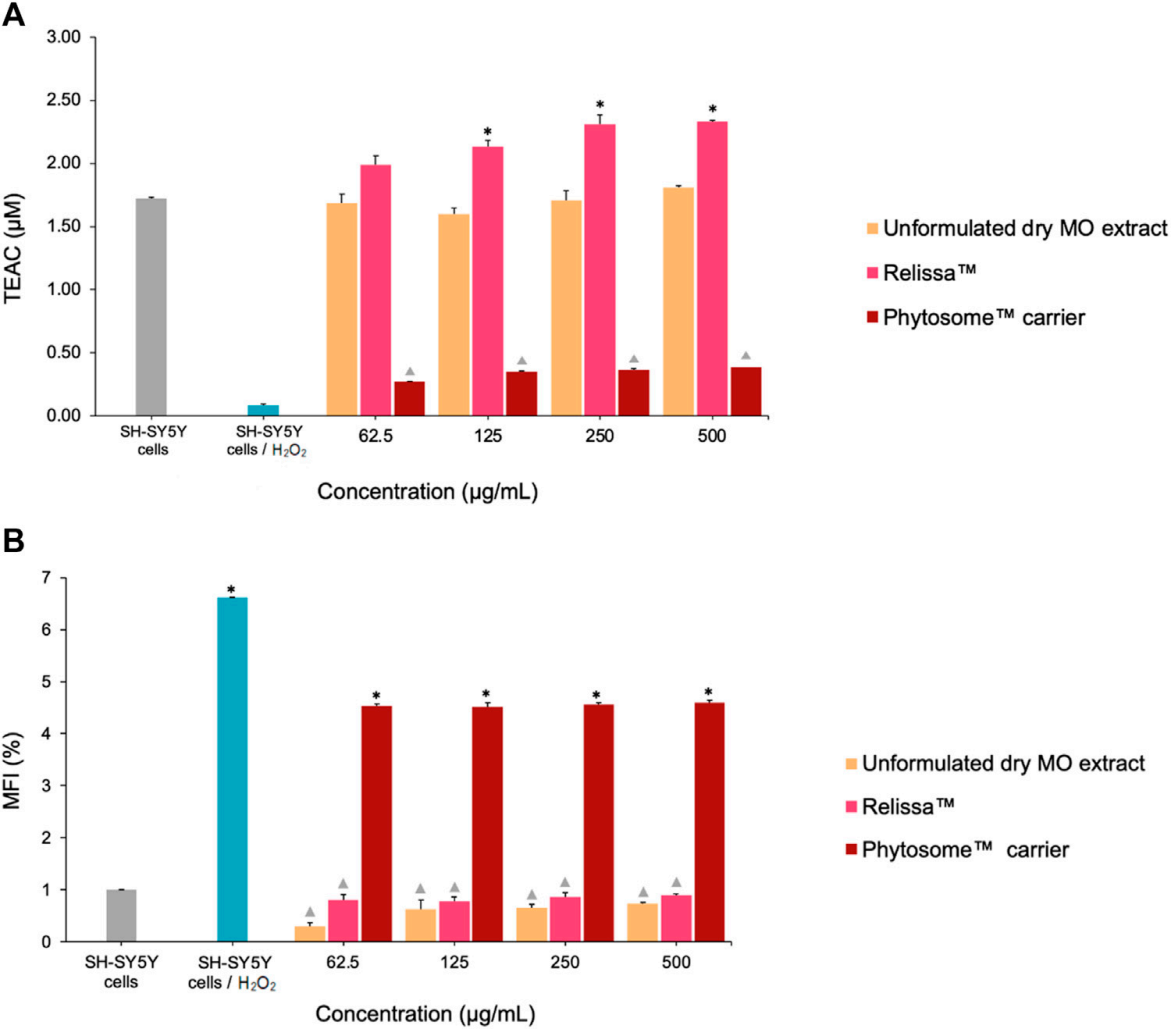
Protective effect of the MO extract on SH-SY5Y cells exposed to H_2_O_2_-induced oxidative stress in the **(A)** TAS and **(B)** total ROS assays. TEAC, Trolox Equivalent Antioxidant Capacity; MFI, mean fluorescence intensity. *p < 0.05, Relissa™ compared to unformulated dry MO extract; 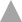 *p* < 0.05, Phytosome™ carrier compared to Relissa™ and unformulated dry MO extract. Error bars represent standard deviation from the mean of three separate measurements.

In SH-SY5Y cell-based TAS assay (Figure 4A), both Relissa™ and unformulated MO extract demonstrated a high protective effect on the cells against H_2_O_2_-induced oxidative stress (Law et al., 2014; Nieto et al., 2018; Morán-Santibañez et al., 2019; Deligoz and Cumaoglu, 2023) even at low concentrations tested under the assay conditions used. However, the protective effect of Relissa™ was significantly stronger than that of the unformulated dry MO extract (*p < 0.05). The Phytosome™ carrier alone did not exhibit any noticeable protective effect (*p* < 0.05).

Similarly, in the ROS production assay (Figure 4B), both the Relissa™ and unformulated dry MO extracts exhibited a strong and comparable protective effect on SH-SY5Y cells against H_2_O_2_-induced oxidative stress (Law et al., 2014; Nieto et al., 2018; Morán-Santibañez et al., 2019; Deligoz and Cumaoglu, 2023) and demonstrated an inhibitory effect on the generation of ROS. The Phytosome™ carrier alone did not show any noticeable protective effects (*p* < 0.05). It is worth to mention that all concentrations of Relissa™ utilized in the cell-based assays are devoid of any cytotoxic effect (see Section 4.4).

### 4.4 Cytotoxicity assays

#### 4.4.1 MTT assay

In the SH-SY5Y cells viability MTT assay, both the Relissa™ and unformulated dry MO extracts, as well as the Phytosome™ carrier alone, showed no cytotoxic effects up to a concentration of 5,000 μg/mL, demonstrating the extract/carrier excellent safety and tolerability on SH-SY5Y cells (Supplementary Information S10).

**FIGURE 5:**
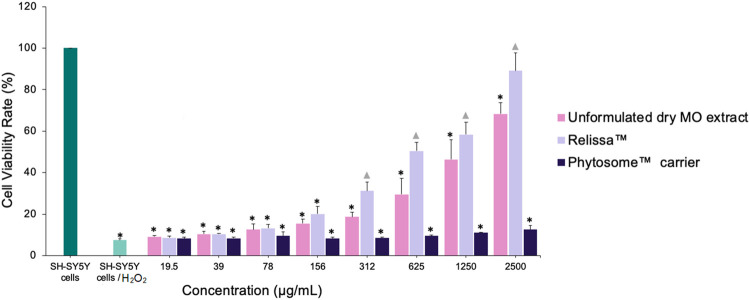
Protective effect of MO extract on SH-SY5Y cells exposed to H_2_O_2_-induced oxidative stress, as shown in the MTT assay. *p < 0.05, compared to SH-SY5Y control cells; 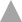 *p* < 0.05, compared to SH-SY5Y cells/H_2_O_2_. Error bars represent standard deviation from the mean of three separate measurements.

In another MTT assay, H_2_O_2_ was used as an oxidative stress inducer. The addition of H_2_O_2_ resulted in a significant decrease (*p < 0.001) in SH-SY5Y cells viability (Figure 5) due to cellular oxidative damage. However, in a dose-dependent manner, the presence of Relissa™ and unformulated dry MO extracts exerted a significant protective effect (*p < 0.05) on SH-SY5Y cells and increased their viability. The protective effect of Relissa™ was improved compared to that observed for the unformulated dry MO extract (*p < 0.05). The Phytosome™ carrier alone did not exhibit any protective effect on SH-SY5Y cells viability against H_2_O_2_-induced oxidative damage.

#### 4.4.2 LDH assay

**FIGURE 6:**
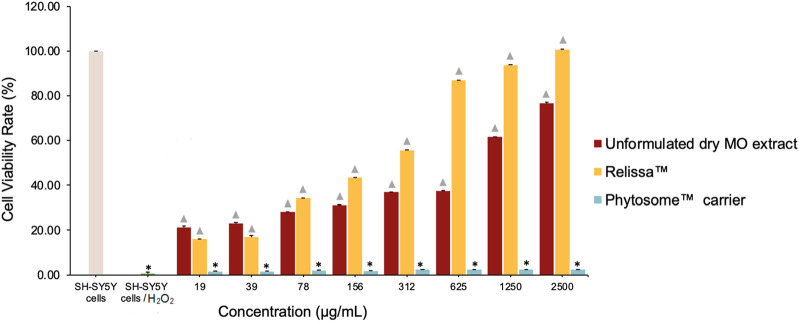
Protective effect of MO extract on SH-SY5Y cells exposed to H_2_O_2_-induced oxidative stress in the LDH assay. *p < 0.05, compared to SH-SY5Y control cells; 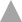 *p* < 0.05, compared to SH-SY5Y cells/H_2_O_2_. Error bars represent standard deviation from the mean of three separate measurements.

Like the MTT assay, similar results were observed in the LDH assay. In a dose-dependent manner, Relissa™ resulted in a significant increase in the viability of SH-SY5Y cells exposed to H_2_O_2_-induced oxidative stress compared to unformulated dry MO extract (*p < 0.05) (Figure 6). The Phytosome™ carrier alone did not reveal any protective effect on SH-SY5Y cells viability against the H_2_O_2_-induced oxidative damage.

### 4.5 Cell-free system antioxidant activity assays

#### 4.5.1 TEAC-ABTS, DPPH, and FRAP assays

**FIGURE 7:**
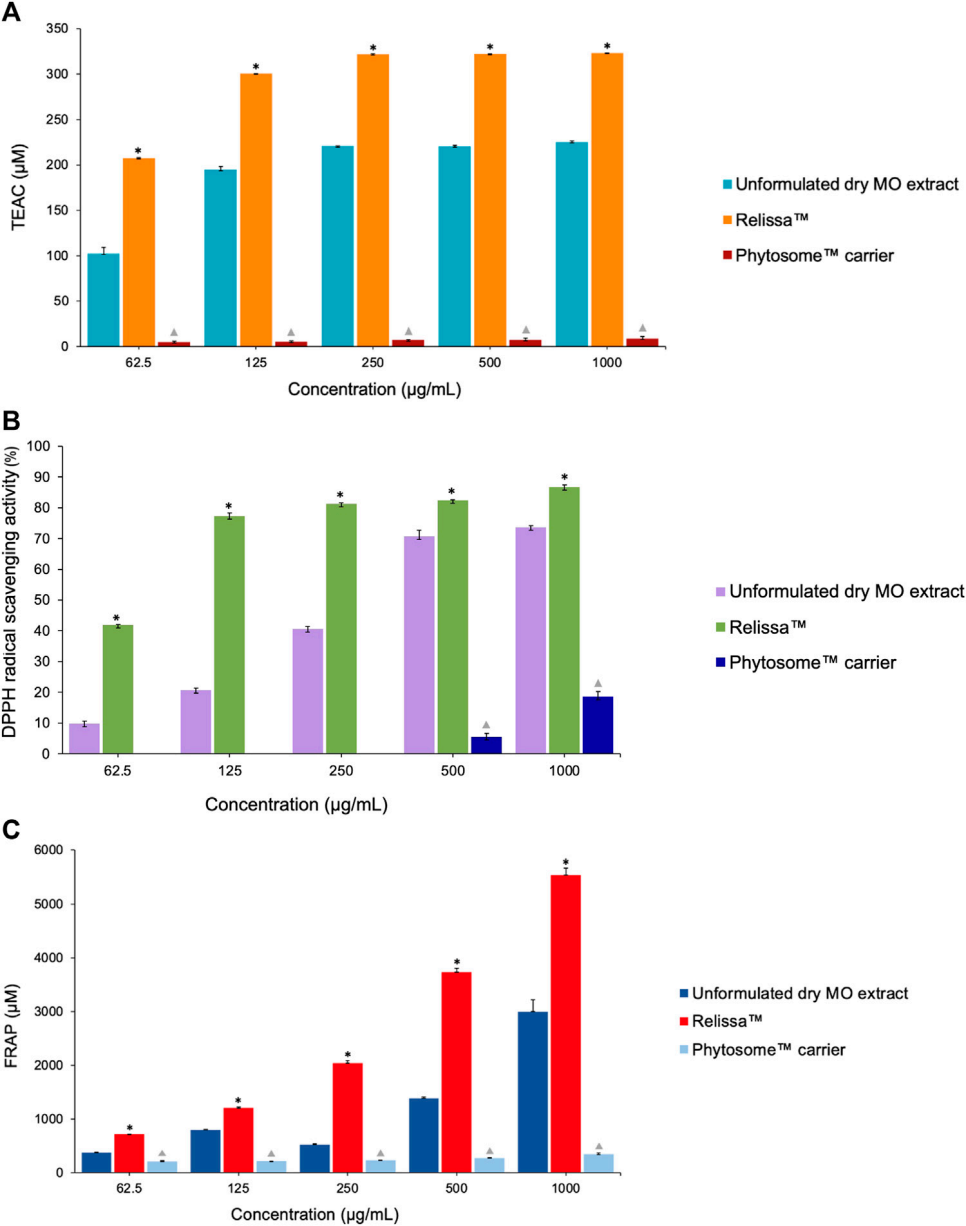
Antioxidant effect of the MO extract as shown by cell-free system chemical tests **(A)** (TEAC)-ABTS, **(B)** DPPH, and **(C)** FRAP assays. *p < 0.05, Relissa™ compared to unformulated dry MO extract; 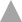 *p* < 0.05, Phytosome™ carrier compared to Relissa™ and the unformulated dry MO extract. Error bars represent standard deviation from the mean of three separate measurements.

In both the TEAC-ABTS (Figure 7A) and DPPH (Figure 7B) assays, with the extracts tested in low-to-higher concentrations under the assay conditions, Relissa™ solution exhibited a significantly strong antioxidant effect even at a low concentration, while the unformulated dry MO extract showed an increase in the antioxidant effect in a concentration-dependent manner. At all concentrations tested, the antioxidant effect of Relissa™ was significantly stronger than that of the unformulated dry MO extract in both the TEAC-ABTS and DPPH antioxidant activity assays (*p < 0.05).

In the FRAP antioxidant activity test (Figure 7C), both the Relissa™ and unformulated dry MO extracts showed antioxidant effects in a dose-dependent manner. The antioxidant effect of Relissa™ was significantly stronger than that of the unformulated dry MO extract (*p < 0.05).

In all the three antioxidant activity tests (TEAC-ABTS, DPPH, and FRAP assays), the Phytosome™ carrier alone did not exhibit any noticeable antioxidant effect (*p* < 0.05).

#### 4.5.2 ORAC and HORAC assays

**FIGURE 8:**
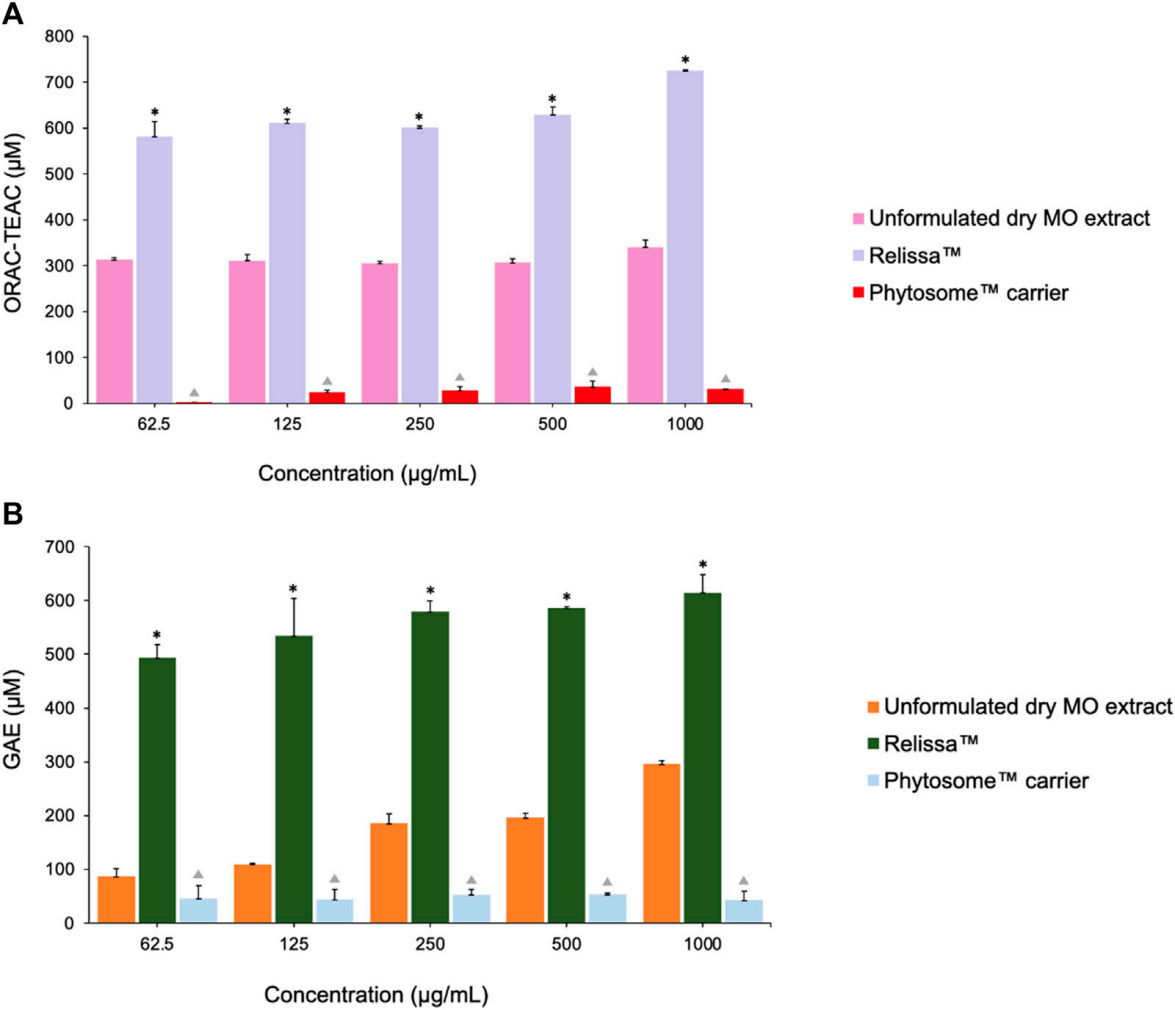
Antioxidant effects of MO extracts as shown by the **(A)** ORAC and **(B)** HORAC assays. Error bars represent standard deviation from the mean of three separate measurements. *p < 0.05, Relissa™ compared to unformulated dry MO extract; 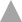 *p* < 0.05, Phytosome™ carrier compared to Relissa™ and unformulated dry MO extract; GAE, gallic acid equivalent.

In the ORAC antioxidant activity test (Figure 8A), even at low concentrations tested under the assay conditions, Relissa™ exhibited a stronger antioxidant effect than that of the unformulated MO extract (*p < 0.05). The antioxidant effect of unformulated MO extract increased in a dose-dependent manner but was significantly weaker than that of Relissa™ at all concentrations tested under the assay conditions.

Similarly, in the HORAC antioxidant activity test (Figure 8B), Relissa™ exhibited a stronger antioxidant effect even at a low concentration than that of the unformulated dry MO extract (*p < 0.05). An increase in concentration of the unformulated dry MO extract did not lead to an increase in its antioxidant effect. In both the ORAC and HORAC assays, the Phytosome™ carrier alone did not exhibit any noticeable antioxidant effect (*p* < 0.05).

## 5 Discussion

In this study, for the first time, the possible mechanism of the beneficial effects of an innovative standardized phospholipid carrier-based (Phytosome™) MO extract (Relissa™) compared to an unformulated dry MO extract on mood modulation and neuroprotective effects against oxidative stress was evaluated in *in vitro* assays. The results revealed that Relissa™ possesses a strong inhibitory activity against the GABA-T enzyme, while the unformulated dry MO extract poorly inhibits GABA-T. These results are in accordance with the findings first reported by Awad et al. (2007), who showed that both the MO aqueous and ethanolic extract had considerable GABA-T inhibitory activity with IC_50_ = 0.35 and 0.79 mg/mL, respectively. In a second study, the authors reported the GABA-T inhibitory activity of aqueous MO extract (yield 10.6%) IC_50_ (mg/mL) (95% CI) = 0.82, methanolic extract (yield 8.9%) IC_50_ = 0.55, and ethyl acetate extract (yield 2%) IC_50_ = 2.55^20^.

GABA is a fundamental neurotransmitter in the mammalian CNS and plays a pivotal role in the pathophysiology of anxious states. Low brain GABA levels lead to hyperactivity and have been implicated in mental health disorders, including anxiety, depression, epilepsy, Parkinson’s disease, Alzheimer’s disease, Huntington’s chorea, stiff-man syndrome, and various other motor neuron diseases (Pearl and Gibson, 2004). In recent years, the GABAergic transmission system has attracted increasing scientific interest as a therapeutic target for the treatment of anxiety. It has been suggested that the symptoms of anxiety can be controlled by increasing the GABA levels in the brain, which may be achieved by inhibiting GABA-T (Ashton and Young, 2003), the enzyme responsible for the catabolism of the GABA neurotransmitter. The inhibitory effect of GABA-T by Relissa™ supports its psychoneurological role as a CNS-calming agent, and its supplementation may help in the amelioration of symptoms of anxiety and associated conditions by upregulating the GABA brain levels via the inhibition of GABA-T.

The present study also revealed the potential inhibitory activity of MAO-A by Relissa™ compared to unformulated dry MO extract. These results are consistent with results reported by López et al. (2009). MAO-A is a crucial enzyme in the CNS, and it regulates levels of all three major monoamine neurotransmitters (serotonin, norepinephrine, and dopamine) in the brain (Shih et al., 1999; Naoi et al., 2018). The inhibition of MAO-A has been linked to the alleviation of depression symptoms (Rang and Dale, 2003). The results of MAO-A inhibition by Relissa™ further support the beneficial effects of Relissa™ as a calming agent that may help in the alleviation of low mood/stress conditions via upregulation of the monoamine neurotransmitters in the CNS by inhibiting the MAO-A enzyme.

The GABA-T and MAO-A inhibitory properties of Relissa™ are of particular interest for its applications in low-mood states and associated conditions such as sleep disorders, which are intricately interconnected with emotional distress, reflecting a bidirectional relationship that has been extensively documented in scientific literature (Philbrook and Macdonald-Gagnon, 2021; Yasugaki et al., 2023). A distinct mechanism supporting this connection is the disruption of circadian rhythms and the dysregulation of neurochemical processes that directly influence mood and emotion (Armitage, 2007; Palagini et al., 2022). Chronic sleep disorders can lead to impaired cognitive function, reduced resilience to stressors, and chronic fatigue, all of which contribute to mental health vulnerability (Vargas et al., 2015). Conversely, mental health distress can also perpetuate or exacerbate sleep disorders (Cipriani et al., 2021). Conditions such as anxiety and low-mood states are frequently noted to be accompanied by insomnia or hypersomnia, whereby emotions disrupt the quantity and quality of sleep (Lustberg and Reynolds, 2000). The resultant negative impact on sleep quality can further worsen the individual’s mental health, creating a self-reinforcing cycle. Given that Relissa™ possesses high GABA-T and MAO-A inhibitory activity, it is proposed that its supplementation may, therefore, also help in the improvement of sleep disorder conditions.

In this study, Relissa™ also increased the expression levels of BDNF in SH-SY5Y cells exposed to H_2_O_2_-induced oxidative stress. BDNF is one of the major neurotrophic factors that play key roles in the development and survival of neurons, development of neuronal processes, and synaptic plasticity. An aberrant level of BDNF expression is closely associated with the pathophysiology of numerous neurological disorders, including depression (neurotrophic hypothesis of depression) (Dwivedi, 2009; Castrén and Monteggia, 2021; Duman et al., 2021), Alzheimer’s disease, Parkinson’s disease, and Huntington’s disease (Murer et al., 2001; Lee et al., 2005; Peng et al., 2005). The results of the present study show that Relissa™ can possibly help in emotional distress conditions by upregulating the BDNF expression levels in the brain and improving neuronal plasticity.

The present study also evaluated the antioxidative stress activity of the MO extract in SH-SY5Y human neuroblastoma cells, commonly used as neuronal models, as well as in cell-free chemical tests. Overall, in both SH-SY5Y cell line (exposed to H_2_O_2_-induced oxidative stress) system-based TAS and ROS assays and in cell-free tests including TEAC-ABTS, DPPH, FRAP, ORAC, and HORAC assays, Relissa™ exhibited significant antioxidant effects compared to unformulated dry MO extract. These results support the favorable bioavailability and effectiveness of Relissa™ for its neuroprotective activity against oxidative stress damage. Moreover, the antioxidant effect of MO extracts evaluated in the present study is in agreement with previously reported studies (Dastmalchi et al., 2008; López et al., 2009; Özkol et al., 2011; Lin et al., 2012; Martins et al., 2012; Ghazizadeh et al., 2020; Abd Allah et al., 2022; Abo-Zaid et al., 2023).

The link between unbalanced oxidative stress and emotional distress is well-known. Oxidative stress mechanism has been implicated in the pathogenesis of various neurological disorders, such as depression (Correia et al., 2023), anxiety (Bouayed et al., 2009), stress, Parkinson’s disease, Alzheimer’s disease, Huntington’s disease, amyotrophic lateral sclerosis, schizophrenia (Yao et al., 2001), major depressive disorder (Bilici et al., 2001), and bipolar disorder (Ng et al., 2008; Pizzino et al., 2017). The brain is intrinsically vulnerable to oxidative stress due to its high O_2_ consumption, modest antioxidant defenses, and lipid-rich constitution (Halliwell, 2006; Ng et al., 2008). When the production of oxygen-derived metabolites surpasses the brain’s defense systems, oxidative damage can occur in the nucleic acids, proteins, and neuronal membrane lipids (Delattre et al., 2005; Halliwell, 2006; Valko et al., 2007). In the presence of oxidative stress, the lipid-rich constitution of brain favors lipid peroxidation, resulting in the decrease in membrane fluidity and damage to membrane proteins, inactivating receptors, enzymes, and ion channels (Delattre et al., 2005; Halliwell, 2006; Valko et al., 2007). As a result, oxidative stress can alter neurotransmission, neuronal function, and overall brain activity (LeBel and Bondy, 1991; Cardozo-Pelaez et al., 1999; Delattre et al., 2005). It is widely accepted that oxidative damage in the brain has potential to trigger processes such as inflammation, neurodegeneration, and neuronal death, causing an impairment of the nervous system (Correia et al., 2023). According to the results of the present study, Relissa™ possesses considerable biological potential to play a role in the neuronal antioxidant defense system and can, therefore, ameliorate symptoms of emotional distress and related conditions such as sleep disorder.

Finally, the present study also demonstrated that both Relissa™ and unformulated dry MO extract treatments at concentrations up to 5,000 μg/mL have no toxicity effect on SH-SY5Y cells viability in the MTT assay. In the MTT assay with SH-SY5Y cells exposed to H_2_O_2_-induced toxicity (oxidative stress), MO extract at a concentration of 2,500 μg/mL demonstrated a strong protective effect on SH-SY5Y cells against oxidative damage. This result was also demonstrated in the LDH release assay, which is in agreement with the previously reported study by López et al. (2009), and supports the neuroprotective effect of MO extracts against oxidative stress and toxicity.

It is noteworthy that the results of the present study suggest that the Phytosome™ carrier system allows an enhancing power of MO leaf extract phytonutrients, as suggested by other studies on botanical extracts (Riva et al., 2019a; Riva et al., 2019b; Rondanelli et al., 2021; Pivari et al., 2022; Bano et al., 2023). The application of the Phytosome™ carrier technology to the MO extract allowed it to confer more stability to oxidation and cellular accessibility compared to unformulated MO extract, with consequent benefits of improved biological activity. In terms of the chemical constituents, MO leaf extract contains a variety of compounds, primarily polyphenolics, flavonoids, and terpenes, with rosmarinic acid as the major constituent (Carnat et al., 1998; Žiaková et al., 2003; Dastmalchi et al., 2008; Awad et al., 2009; Popova et al., 2016). The anxiolytic and mood-calming effects of MO have been documented in numerous studies; however, the mechanism(s) of action are unclear, and only a few studies have investigated its potential role in the CNS. Awad et al. (2009) showed that MO extract GABA-T inhibitory activity is primarily due to rosmarinic acid and, to a smaller extent, due to other triterpenoids, such as ursolic acid and oleanolic acid. Numerous preclinical studies have revealed the efficacy of rosmarinic acid against several neuropsychiatric disorders, including anxiety, depression, and sleep disorders (Ghasemzadeh Rahbardar and Hosseinzadeh, 2020; Dahchour, 2022).

Based on the results of the revealed mechanisms of action investigated in the present study, Bano et al. (2023) carried out a perspective, double-blinded, placebo-controlled randomized clinical trial of Relissa™ supplementation in healthy adults with a moderate level of depression, anxiety, stress, and sleep disorders. The clinical outcomes confirmed a significant improvement in all the above-mentioned conditions of emotional distress in subjects who received the Relissa™ supplementation compared to those who received the placebo, supporting the beneficial effects of Relissa™ supplementation in low-mood states.

In conclusion, the results of the present study suggest that Relissa™ possesses considerable neuroprotective, mood improving, anxiolytic, antioxidant, and anti-inflammatory pharmacological properties. These effects potentially support the role of Relissa™ as a CNS-calming agent, and its supplementation can possibly help in the management of emotional distress conditions such as anxiety, stress, low-mood states, and related conditions such as sleep disorders.

## Data Availability

The original contributions presented in the study are included in the article/Supplementary Material; further inquiries can be directed to the corresponding authors.

## References

[B1] Abd Allah H. N. Abdul-Hamid M. Mahmoud A. M. Abdel-Reheim E. S. (2022). *Melissa officinalis* L. ameliorates oxidative stress and inflammation and upregulates Nrf2/HO-1 signaling in the hippocampus of pilocarpine-induced rats. Environ. Sci. Pollut. Res. Int. 29 (2), 2214–2226. 10.1007/s11356-021-15825-y 34363578

[B2] Abo-Zaid O. A. R. Moawed F. S. Taha E. F. Ahmed E. S. A. Kawara R. S. (2023). *Melissa officinalis* extract suppresses endoplasmic reticulum stress-induced apoptosis in the brain of hypothyroidism-induced rats exposed to γ-radiation. Cell. Stress Chaperones 28 (6), 709–720. 10.1007/s12192-023-01363-8 37368180 PMC10746611

[B3] Akhondzadeh S. Noroozian M. Mohammadi M. Ohadinia S. Jamshidi A. H. Khani M. (2003). *Melissa officinalis* extract in the treatment of patients with mild to moderate Alzheimer's disease: a double blind, randomised, placebo controlled trial. J. Neurol. Neurosurg. Psychiatry 74 (7), 863–866. 10.1136/jnnp.74.7.863 12810768 PMC1738567

[B4] Alijaniha F. Naseri M. Afsharypuor S. Fallahi F. Noorbala A. Mosaddegh M. (2015). Heart palpitation relief with *Melissa officinalis* leaf extract: double blind, randomized, placebo controlled trial of efficacy and safety. J. Ethnopharmacol. 164, 378–384. 10.1016/j.jep.2015.02.007 25680840

[B5] Arceusz A. Wesolowski M. Ulewicz-Magulska B. (2015). Flavonoids and phenolic acids in methanolic extracts, infusions and tinctures from commercial samples of lemon balm. Nat. Prod. Commun. 10 (6), 1934578X1501000–81. 10.1177/1934578x1501000645 26197530

[B6] Armitage R. (2007). Sleep and circadian rhythms in mood disorders. Acta Psychiatr. Scand. Suppl. 115 (433), 104–115. 10.1111/j.1600-0447.2007.00968.x 17280576

[B7] Ashton H. Young A. H. (2003). GABA-ergic drugs: exit stage left, enter stage right. J. Psychopharmacol. 17 (2), 174–178. 10.1177/0269881103017002004 12870563

[B8] Awad R. Levac D. Cybulska P. Merali Z. Trudeau V. L. Arnason J. T. (2007). Effects of traditionally used anxiolytic botanicals on enzymes of the γ-aminobutyric acid (GABA) system. Can. J. physiology Pharmacol. 85 (9), 933–942. 10.1139/Y07-083 18066140

[B9] Awad R. Muhammad A. Durst T. Trudeau V. L. Arnason J. T. (2009). Bioassay‐guided fractionation of lemon balm (*Melissa officinalis* L.) using an *in vitro* measure of GABA transaminase activity. Phytotherapy Res. Int. J. Devoted Pharmacol. Toxicol. Eval. Nat. Prod. Deriv. 23 (8), 1075–1081. 10.1002/ptr.2712 19165747

[B10] Baldwin D. S. Waldman S. Allgulander C. (2011). Evidence-based pharmacological treatment of generalized anxiety disorder. Int. J. Neuropsychopharmacol. 14 (5), 697–710. 10.1017/S1461145710001434 21211105

[B11] Bano A. Hepsomali P. Rabbani F. Farooq U. Kanwal A. Saleem A. (2023). The possible “calming effect” of subchronic supplementation of a standardised phospholipid carrier-based *Melissa officinalis* L. extract in healthy adults with emotional distress and poor sleep conditions: results from a prospective, randomised, double-blinded, placebo-controlled clinical trial . Front. Pharmacol. 14, 1250560. 10.3389/fphar.2023.1250560 37927585 PMC10620697

[B12] Bilici M. Efe H. Köroğlu M. A. Uydu H. A. Bekaroğlu M. Değer O. (2001). Antioxidative enzyme activities and lipid peroxidation in major depression: alterations by antidepressant treatments. J. Affect Disord. 64 (1), 43–51. 10.1016/s0165-0327(00)00199-3 11292519

[B13] Bouayed J. Rammal H. Soulimani R. (2009). Oxidative stress and anxiety: relationship and cellular pathways. Oxid. Med. Cell. Longev. 2 (2), 63–67. 10.4161/oxim.2.2.7944 20357926 PMC2763246

[B14] Brand-Williams W. Cuvelier M. E. Berset C. (1995). Use of a free radical method to evaluate antioxidant activity. LWT - Food Sci. Technol. 28 (1), 25–30. 10.1016/s0023-6438(95)80008-5

[B15] Cardozo-Pelaez F. Song S. Parthasarathy A. Hazzi C. Naidu K. Sanchez-Ramos J. (1999). Oxidative DNA damage in the aging mouse brain. Mov. Disord. 14 (6), 972–980. 10.1002/1531-8257(199911)14:6<972::aid-mds1010>3.0.co;2-0 10584672

[B16] Carnat A. P. Fraisse D. Lamaison J. (1998). The aromatic and polyphenolic composition of lemon balm (*Melissa officinalis* L. subsp. officinalis) tea. Pharm. Acta Helvetiae 72 (5), 301–305. 10.1016/s0031-6865(97)00026-5

[B17] Cases J. Ibarra A. Feuillère N. Roller M. Sukkar S. G. (2011). Pilot trial of *Melissa officinalis* L. leaf extract in the treatment of volunteers suffering from mild-to-moderate anxiety disorders and sleep disturbances. Med. J. Nutr. Metab. 4 (3), 211–218. 10.1007/s12349-010-0045-4 PMC3230760 22207903

[B18] Castrén E. Monteggia L. M. (2021). Brain-derived neurotrophic factor signaling in depression and antidepressant action. Biol. Psychiatry 90 (2), 128–136. 10.1016/j.biopsych.2021.05.008 34053675

[B19] Cipriani G. E. Bartoli M. Amanzio M. (2021). Are sleep problems related to psychological distress in healthy aging during the COVID-19 pandemic? A review. Int. J. Environ. Res. Public Health 18 (20), 10676. 10.3390/ijerph182010676 34682423 PMC8536178

[B20] Correia A. S. Cardoso A. Vale N. (2023). Oxidative stress in depression: the link with the stress response, neuroinflammation, serotonin, neurogenesis and synaptic plasticity. Antioxidants (Basel) 12 (2), 470. 10.3390/antiox12020470 36830028 PMC9951986

[B21] Dahchour A. (2022). Anxiolytic and antidepressive potentials of rosmarinic acid: a review with a focus on antioxidant and anti-inflammatory effects. Pharmacol. Res. 184, 106421. 10.1016/j.phrs.2022.106421 36096427

[B22] Dastmalchi K. Damien Dorman H. Oinonen P. P. Darwis Y. Laakso I. Hiltunen R. (2008). Chemical composition and *in vitro* antioxidative activity of a lemon balm (*Melissa officinalis* L.) extract. LWT - Food Sci. Technol. 41 (3), 391–400. 10.1016/j.lwt.2007.03.007

[B23] Delattre J. Beaudeux J. Bonnefont-Rousselot D. (2005). Radicaux libres et stress oxydant: aspects biologiques et pathologiques: Éditions Tec & doc. Paris: France.

[B24] Deligoz H. Cumaoglu A. (2023). Hydrogen peroxide-induced oxidative stress and apoptosis in SH-SY5Y cells: protective effect of Momordica charantia fruit extract. J. Exp. Clin. Med. 40 (3), 497–501. 10.52142/omujecm.40.3.13

[B25] Duman R. S. Deyama S. Fogaça M. V. (2021). Role of BDNF in the pathophysiology and treatment of depression: activity-dependent effects distinguish rapid-acting antidepressants. Eur. J. Neurosci. 53 (1), 126–139. 10.1111/ejn.14630 31811669 PMC7274898

[B26] Dwivedi Y. (2009). Brain-derived neurotrophic factor: role in depression and suicide. Neuropsychiatr. Dis. Treat. 5, 433–449. 10.2147/ndt.s5700 19721723 PMC2732010

[B27] Erel O. (2004). A novel automated direct measurement method for total antioxidant capacity using a new generation, more stable ABTS radical cation. Clin. Biochem. 37 (4), 277–285. 10.1016/j.clinbiochem.2003.11.015 15003729

[B28] Gandhi A. Dutta A. Pal A. Bakshi P. (2012). Recent trends of phytosomes for delivering herbal extract with improved bioavailability. J. Pharmacogn. Phytochemistry 1, 6–14.

[B29] Ghasemzadeh Rahbardar M. Hosseinzadeh H. (2020). Effects of rosmarinic acid on nervous system disorders: an updated review. Naunyn-Schmiedeberg's Archives Pharmacol. 393 (10), 1779–1795. 10.1007/s00210-020-01935-w 32725282

[B30] Ghazizadeh J. Hamedeyazdan S. Torbati M. Farajdokht F. Fakhari A. Mahmoudi J. (2020). *Melissa officinalis* L. hydro-alcoholic extract inhibits anxiety and depression through prevention of central oxidative stress and apoptosis. Exp. Physiol. 105 (4), 707–720. 10.1113/EP088254 32003913

[B31] Halliwell B. (2006). Oxidative stress and neurodegeneration: where are we now? J. Neurochem. 97 (6), 1634–1658. 10.1111/j.1471-4159.2006.03907.x 16805774

[B32] Hasbal G. Yilmaz-Ozden T. Can A. (2015). Antioxidant and antiacetylcholinesterase activities of Sorbus torminalis (L.) Crantz (wild service tree) fruits. J. Food Drug Anal. 23 (1), 57–62. 10.1016/j.jfda.2014.06.006 28911446 PMC9351744

[B33] He Z.-W. Jiang B. S. K. Y. Sun X. H. Tang M. Liu Y. W. Guan L. P. (2023). Benzothiazole-propanamide linker pyrrolidine (morpholine) as monoamine oxidase-B and butyrylcholinesterase inhibitors. Chem. Biodivers. 20, e202301271. 10.1002/cbdv.202301271 37806964

[B34] Jacoby W. (1962). Enzymes of 4-aminobutyrate metabolism. Methods Enzym. 5, 765–779.

[B35] Kara M. Boran T. Öztaş E. Jannuzzi A. T. Özden S. Özhan G. (2022). Zoledronic acid-induced oxidative damage and endoplasmic reticulum stress-mediated apoptosis in human embryonic kidney (HEK-293) cells. J. Biochem. Mol. Toxicol. 36 (8), e23083. 10.1002/jbt.23083 35587103

[B36] Kara M. ÖztaŞ E. Özhan G. (2020). Acetamiprid-induced cyto- and genotoxicity in the AR42J pancreatic cell line. Turk J. Pharm. Sci. 17 (5), 474–479. 10.4274/tjps.galenos.2019.89719 33177926 PMC7650742

[B37] Kenda M. Kočevar Glavač N. Nagy M. Sollner Dolenc M. (2022a). Medicinal plants used for anxiety, depression, or stress treatment: an update. Molecules 27 (18), 6021. 10.3390/molecules27186021 36144755 PMC9500625

[B38] Kennedy D. O. Little W. Haskell C. F. Scholey A. B. (2006). Anxiolytic effects of a combination of *Melissa officinalis* and Valeriana officinalis during laboratory induced stress. Phytother. Res. 20 (2), 96–102. 10.1002/ptr.1787 16444660

[B39] Kennedy D. O. Little W. Scholey A. B. (2004). Attenuation of laboratory-induced stress in humans after acute administration of *Melissa officinalis* (Lemon Balm). Psychosom. Med. 66 (4), 607–613. 10.1097/01.psy.0000132877.72833.71 15272110

[B40] Kennedy D. O. Scholey A. B. Tildesley N. T. J. Perry E. K. Wesnes K. A. (2002). Modulation of mood and cognitive performance following acute administration of *Melissa officinalis* (lemon balm). Pharmacol. Biochem. Behav. 72 (4), 953–964. 10.1016/s0091-3057(02)00777-3 12062586

[B41] Kennedy D. O. Wake G. Savelev S. Tildesley N. T. J. Perry E. K. Wesnes K. A. (2003). Modulation of mood and cognitive performance following acute administration of single doses of *Melissa officinalis* (Lemon balm) with human CNS nicotinic and muscarinic receptor-binding properties. Neuropsychopharmacology 28 (10), 1871–1881. 10.1038/sj.npp.1300230 12888775

[B42] Khawam E. A. Laurencic G. Malone D. A. Jr. (2006). Side effects of antidepressants: an overview. Cleve Clin. J. Med. 73 (4), 351–353. 10.3949/ccjm.73.4.351 16610395

[B43] Law B. N. Ling A. P. K. Koh R. Y. Chye S. M. Wong Y. P. (2014). Neuroprotective effects of orientin on hydrogen peroxide-induced apoptosis in SH-SY5Y cells. Mol. Med. Rep. 9 (3), 947–954. 10.3892/mmr.2013.1878 24366367

[B44] LeBel C. P. Bondy S. C. (1991). Oxygen radicals: common mediators of neurotoxicity. Neurotoxicol Teratol. 13 (3), 341–346. 10.1016/0892-0362(91)90081-7 1886545

[B45] Lee J. Fukumoto H. Orne J. Klucken J. Raju S. Vanderburg C. R. (2005). Decreased levels of BDNF protein in Alzheimer temporal cortex are independent of BDNF polymorphisms. Exp. Neurol. 194 (1), 91–96. 10.1016/j.expneurol.2005.01.026 15899246

[B46] Lin J.-T. Chen Y. C. Lee Y. C. Rolis Hou C. W. Chen F. L. Yang D. J. (2012). Antioxidant, anti-proliferative and cyclooxygenase-2 inhibitory activities of ethanolic extracts from lemon balm (*Melissa officinalis* L.) leaves. LWT 49 (1), 1–7. 10.1016/j.lwt.2012.04.009

[B47] López V. Martín S. Gómez-Serranillos M. P. Carretero M. E. Jäger A. K. Calvo M. I. (2009). Neuroprotective and neurological properties of *Melissa officinalis* . Neurochem. Res. 34 (11), 1955–1961. 10.1007/s11064-009-9981-0 19760174

[B48] Lopez-Suarez L. Awabdh S. A. Coumoul X. Chauvet C. (2022). The SH-SY5Y human neuroblastoma cell line, a relevant *in vitro* cell model for investigating neurotoxicology in human: focus on organic pollutants. Neurotoxicology 92, 131–155. 10.1016/j.neuro.2022.07.008 35914637

[B49] Lustberg L. Reynolds C. F. (2000). Depression and insomnia: questions of cause and effect. Sleep. Med. Rev. 4 (3), 253–262. 10.1053/smrv.1999.0075 12531168

[B50] Martins E. N. Pessano N. T. C. Leal L. Roos D. H. Folmer V. Puntel G. O. (2012). Protective effect of *Melissa officinalis* aqueous extract against Mn-induced oxidative stress in chronically exposed mice. Brain Res. Bull. 87 (1), 74–79. 10.1016/j.brainresbull.2011.10.003 22020131

[B51] Morán-Santibañez K. Vasquez A. H. Varela-Ramirez A. Henderson V. Sweeney J. Odero-Marah V. (2019). Larrea tridentata extract mitigates oxidative stress-induced cytotoxicity in human neuroblastoma SH-SY5Y cells. Antioxidants (Basel) 8 (10), 427. 10.3390/antiox8100427 31557847 PMC6827101

[B52] Murer M. G. Yan Q. Raisman-Vozari R. (2001). Brain-derived neurotrophic factor in the control human brain, and in Alzheimer's disease and Parkinson's disease. Prog. Neurobiol. 63 (1), 71–124. 10.1016/s0301-0082(00)00014-9 11040419

[B53] Naoi M. Maruyama W. Shamoto-Nagai M. (2018). Type A monoamine oxidase and serotonin are coordinately involved in depressive disorders: from neurotransmitter imbalance to impaired neurogenesis. J. Neural Transm. 125 (1), 53–66. 10.1007/s00702-017-1709-8 28293733

[B54] Ng F. Berk M. Dean O. Bush A. I. (2008). Oxidative stress in psychiatric disorders: evidence base and therapeutic implications. Int. J. Neuropsychopharmacol. 11 (6), 851–876. 10.1017/S1461145707008401 18205981

[B55] Nieto C. I. Cornago M. P. Cabildo M. P. Sanz D. Claramunt R. M. Torralba M. C. (2018). Evaluation of the antioxidant and neuroprotectant activities of new asymmetrical 1,3-diketones. Molecules 23 (8), 1837. 10.3390/molecules23081837 30042315 PMC6222706

[B56] Noguchi-Shinohara M. Ono K. Hamaguchi T. Nagai T. Kobayashi S. Komatsu J. (2020). Safety and efficacy of *Melissa officinalis* extract containing rosmarinic acid in the prevention of Alzheimer’s disease progression. Sci. Rep. 10 (1), 18627. 10.1038/s41598-020-73729-2 33122694 PMC7596544

[B57] Özkol H. Koyuncu I. Tülüce Y. (2011). Some medicinal plants counteract alterations of neuroendocrine stress response system, oxidative and nitrosative stress caused by repeated restraint in rats. J. Med. Plants Res. 5.

[B58] Palagini L. Miniati M. Marazziti D. Massa L. Grassi L. Geoffroy P. A. (2022). Circadian rhythm alterations may be related to impaired resilience, emotional dysregulation and to the severity of mood features in bipolar I and II disorders. Clin. Neuropsychiatry 19 (3), 174–186. 10.36131/cnfioritieditore20220306 35821870 PMC9263680

[B59] Pearl P. L. Gibson K. M. (2004). Clinical aspects of the disorders of GABA metabolism in children. Curr. Opin. Neurol. 17 (2), 107–113. 10.1097/00019052-200404000-00005 15021235

[B60] Peng S. Wuu J. Mufson E. J. Fahnestock M. (2005). Precursor form of brain-derived neurotrophic factor and mature brain-derived neurotrophic factor are decreased in the pre-clinical stages of Alzheimer's disease. J. Neurochem. 93 (6), 1412–1421. 10.1111/j.1471-4159.2005.03135.x 15935057

[B61] Philbrook L. E. Macdonald-Gagnon G. E. (2021). Bidirectional relations between sleep and emotional distress in college students: loneliness as a moderator. J. Genet. Psychol. 182 (5), 361–373. 10.1080/00221325.2021.1913982 33952050

[B62] Pivari F. Mingione A. Piazzini G. Ceccarani C. Ottaviano E. Brasacchio C. (2022). Curcumin supplementation (Meriva(®)) modulates inflammation, lipid peroxidation and gut microbiota composition in chronic kidney disease. Nutrients 14 (1), 231. 10.3390/nu14010231 35011106 PMC8747135

[B63] Pizzino G. Irrera N. Cucinotta M. Pallio G. Mannino F. Arcoraci V. (2017). Oxidative stress: harms and benefits for human health. Oxid. Med. Cell. Longev. 2017, 8416763. 10.1155/2017/8416763 28819546 PMC5551541

[B64] Popova A. Dalemska Z. Mihaylova D. Hristova I. Alexieva I. (2016). *Melissa officinalis* L.—GC profile and antioxidant activity. Int. J. Pharmacogn. Phytochem. Res. 8, 634–638.

[B65] Rang H. P. Dale M. M. (2003). Pharmacology. Edinburgh: Churchill Livingstone, 456–610.

[B66] Riva A. Giacomelli L. Togni S. Franceschi F. Eggenhoffner R. Zuccarini M. C. (2019a). Oral administration of a lecithin-based delivery form of boswellic acids (Casperome®) for the prevention of symptoms of irritable bowel syndrome: a randomized clinical study. Minerva Gastroenterol. Dietol. 65 (1), 30–35. 10.23736/S1121-421X.18.02530-8 30676012

[B67] Riva A. Ronchi M. Petrangolini G. Bosisio S. Allegrini P. (2019b). Improved oral absorption of quercetin from quercetin Phytosome®, a new delivery system based on food grade lecithin. Eur. J. Drug Metab. Pharmacokinet. 44 (2), 169–177. 10.1007/s13318-018-0517-3 30328058 PMC6418071

[B68] Romulo A. (2020). The principle of some *in vitro* antioxidant activity methods: review. IOP Conf. Ser. Earth Environ. Sci. 426 (1), 012177. 10.1088/1755-1315/426/1/012177

[B69] Rondanelli M. Riva A. Petrangolini G. Allegrini P. Giacosa A. Fazia T. (2021). Berberine phospholipid is an effective insulin sensitizer and improves metabolic and hormonal disorders in women with polycystic ovary syndrome: a one-group pretest-Post-test explanatory study. Nutrients 13 (10), 3665. 10.3390/nu13103665 34684666 PMC8538182

[B70] Rush A. J. (2007). STAR*D: what have we learned? Am. J. Psychiatry 164 (2), 201–204. 10.1176/ajp.2007.164.2.201 17267779

[B71] Sahin S. (2016). Modulation der GABAergen Wirkung durch Lebensmittelinhaltsstoffe Doctoral Thesis. Germany: Friedrich-Alexander Universität Erlangen-Nürnberg. Available at: https://open.fau.de/items/293fefef-d522-46ac-a852-64ed774cb498.

[B72] Sahin S. Eulenburg V. Kreis W. Villmann C. Pischetsrieder M. (2016). Three-step test system for the identification of novel GABAA receptor modulating food plants. Plant Foods Hum. Nutr. 71 (4), 355–360. 10.1007/s11130-016-0566-1 27392961

[B73] Sevim Ç. Taghİzadehghalehjoughİ A. Kara M. (2020). *In vitro* investigation of the effects of imidacloprid on AChE, LDH, and GSH levels in the L-929 fibroblast cell line. Turk J. Pharm. Sci. 17 (5), 506–510. 10.4274/tjps.galenos.2019.15807 33177931 PMC7650733

[B74] Sharifi-Rad J. Quispe C. Herrera-Bravo J. Akram M. Abbaass W. Semwal P. (2021). Phytochemical constituents, biological activities, and health-promoting effects of the *Melissa officinalis* . Oxidative Med. Cell. Longev. 2021, 1–20. 10.1155/2021/6584693 PMC11283336 39071243

[B75] Shih J. C. Chen K. Ridd M. J. (1999). Monoamine oxidase: from genes to behavior. Annu. Rev. Neurosci. 22, 197–217. 10.1146/annurev.neuro.22.1.197 10202537 PMC2844879

[B76] Soltanpour A. Alijaniha F. Naseri M. Kazemnejad A. Heidari M. R. (2019). Effects of *Melissa officinalis* on anxiety and sleep quality in patients undergoing coronary artery bypass surgery: a double-blind randomized placebo controlled trial. Eur. J. Integr. Med. 28, 27–32. 10.1016/j.eujim.2019.01.010

[B77] Suryawanshi J. (2011). Phytosome: an emerging trend in herbal drug treatment. J. Med. Gene Geno 3.

[B78] Świąder K. Startek K. Wijaya C. H. (2019). The therapeutic properties of Lemon balm (*Melissa officinalis* L.): reviewing novel findings and medical indications. J. Appl. Bot. Food Qual. 92, 327–335. 10.5073/JABFQ.2019.092.044

[B79] Valko M. Leibfritz D. Moncol J. Cronin M. T. D. Mazur M. Telser J. (2007). Free radicals and antioxidants in normal physiological functions and human disease. Int. J. Biochem. Cell. Biol. 39 (1), 44–84. 10.1016/j.biocel.2006.07.001 16978905

[B80] Vargas I. Friedman N. P. Drake C. L. (2015). Vulnerability to stress-related sleep disturbance and insomnia: investigating the link with comorbid depressive symptoms. Transl. Issues Psychol. Sci. 1 (1), 57–66. 10.1037/tps0000015 25914895 PMC4406050

[B81] Wauquier F. Boutin-Wittrant L. Bouvret E. Le Faouder J. Roux V. Macian N. (2022). Circulating human serum metabolites derived from the intake of a saffron extract (Safr'Inside(TM)) protect neurons from oxidative stress: consideration for depressive disorders. Nutrients 14 (7), 5027. 10.3390/nu14235027 35406124 PMC9002571

[B82] Yao J. K. Reddy R. D. van Kammen D. P. (2001). Oxidative damage and schizophrenia: an overview of the evidence and its therapeutic implications. CNS Drugs 15 (4), 287–310. 10.2165/00023210-200115040-00004 11463134

[B83] Yasugaki S. Okamura H. Kaneko A. Hayashi Y. (2023). Bidirectional relationship between sleep and depression. Neurosci. Res. 10.1016/j.neures.2023.04.006 37116584

[B84] Žiaková A. Brandšteterová E. Blahová E. (2003). Matrix solid-phase dispersion for the liquid chromatographic determination of phenolic acids in *Melissa officinalis* . J. Chromatogr. A 983 (1-2), 271–275. 10.1016/s0021-9673(02)01712-0 12568390

