# Supplementary material for: An in vitro analysis of an innovative standardized phospholipid carrier-based Melissa officinalis L. extract as a potential neuromodulator for emotional distress and related conditions
Source: Front Mol Biosci. 2024 Mar 13;11:1359177. doi: 10.3389/fmolb.2024.1359177 (PMC10965792; doi:10.3389/fmolb.2024.1359177)
Supplement: Supplementary file 1 [file DataSheet1.PDF]

ab284510 – Monoamine Oxidase A (MOA-A) Inhibitor Screening Kit (Fluorometric)

For the screening of potential MOA-A inhibitors.
For research use only - not intended for diagnostic use.

PLEASE NOTE: With the acquisition of BioVision by Abcam, we have made some changes to component names and packaging to better align with our global standards as we work towards environmental-friendly and efficient growth. You are receiving the same high-quality products as always, with no changes to specifications or protocols.

For overview, typical data and additional information please visit:
http://www.abcam.com/ab284510

Storage and Stability

On receipt entire assay kit should be stored at -20°C, protected from light. Kit has a storage time of 1 year from receipt, providing components have not been reconstituted.

Materials Supplied

Item	Quantity	Storage Condition
Assay Buffer XVIII/MAO-A Assay Buffer	25 mL	-20°C
MAO Substrate/MAO-A Substrate	1 vial	-20°C
MAO-A Enzyme	1 vial	-20°C
MAO-A Inhibitor/Inhibitor Control (Clorgyline)	1 vial	-20°C
OxiRed Probe (in DMSO)	0.2 mL	-20°C
Developer Solution V/Developer	1 vial	-20°C

Materials Required, Not Supplied

- 96-well black plate with flat bottom
- Multi-well spectrophotometer
- H_2O_2

Reagent Preparation

– Before using the kit, spin the tubes prior to opening.

Assay Buffer XVIII/MAO-A Assay Buffer: Bring to room temperature before use. Store at -20°C.

OxiRed Probe: Bring to room temperature before use. Protect from light & moisture. Store at -20°C. Stable for two months.

MAO-A Enzyme: Reconstitute with 25 µL Assay Buffer XVIII/MAO-A Assay Buffer. Mix well. Aliquot & store at -80°C. Stable for two months.

MAO Substrate/MAO-A Substrate: Reconstitute with 110 µL ddH_2O. Store at -20°C. Stable for two months.

Developer Solution V/Developer: Reconstitute with 220 µL Assay Buffer XVIII/MAO-A Assay Buffer. Mix well. Store at -20°C. Stable for two months.

MAO-A Inhibitor/Inhibitor Control (Clorgyline): Reconstitute with 250 µL ddH_2O to make a stock solution of 2 mM. Mix well. Make a 10 µM working solution by adding 5 µL of the 2 mM stock solution into 995 µL ddH_2O. Store the stock solution at -20°C. Stable for two months. Inhibitor's working solution can be stored at 4°C to use within 24 hrs.

Assay Protocol

Screening Compounds, Inhibitor Control, and Blank Control Preparations

1. Dissolve candidate test compounds into proper solvent.
2. Dilute to 10X concentration with Assay Buffer XVIII/Assay Buffer.
3. Add 10 µL diluted test compounds (S), working solution of MAO-A Inhibitor/Inhibitor Control (IC) and Assay Buffer XVIII/MAO-A Assay Buffer (Enzyme Control; EC) into assigned wells.

∆ Note: *Preferred final solvent concentration should not be more than 2% by volume. If solvent exceeds 2% include a Solvent Control to test the effect of the solvent on enzyme activity.*

∆ Note: *Optional - To check the possible inhibitory effect of test inhibitors on Developer Solution V/Developer, prepare a parallel test inhibitor well (TI). MAO-A Inhibitor/Inhibitor Control-Clorgyline does not inhibit the Developer Solution V/Developer.*

MAO-A Enzyme Solution preparation:

1. Dilute the Enzyme stock solution 5 times by adding 2 µL of MAO-A Enzyme Stock Solution into 8 µL of Assay Buffer XVIII/MAO-A Assay Buffer.
2. For each well, prepare 50 µL MAO-A Enzyme Solution:

	Enzyme Solution
Assay Buffer XVIII/MAO-A Assay Buffer	49 µL
Diluted MAO-A Enzyme	1 µL

3. Mix. Add 50 µL/well into wells containing test inhibitors, MAO-A Inhibitor/Inhibitor Control, & Enzyme Control. Incubate for 10 minutes at 25°C.

∆ Note: *Always freshly prepare MAO-A Enzyme working solution. Don't store the enzyme working solution.*

∆ Note: *To check the possible inhibitory effect of test inhibitors on Developer Solution V/Developer, replace the 1 µL of diluted MAO-A Enzyme with 1 µL of 10 mM H_2O_2. Mix & add 50 µL to the TI well. Incubate for 10 minutes at 25°C.*

MAO Substrate/MAO-A Substrate Solution Preparation:

1. For each well, prepare 40 µl of MAO-A Substrate Solution:

	Substrate Solution
Assay Buffer XVIII/MAO-A Assay Buffer	37 µL
MAO Substrate/MAO-A Substrate	1 µL
Developer Solution V/Developer	1 µL
OxiRed Probe	1 µL

2. Mix well and add 40 µL of the MAO-A Substrate Solution into each well. Mix well.

Measurement

Measure the fluorescence (Ex/Em = 535/587 nm) kinetically at 25°C for 10-30 min. Choose two points (T_1 and T_2) in the linear range of the plot and obtain the corresponding fluorescence values (RFU_1 and RFU_2).

Calculation

1. Calculate the slope for all samples, including Enzyme Control (EC), by dividing the net ΔRFU ($RFU_2 - RFU_1$) values by the time Δt ($T_2 - T_1$). Calculate % Relative Inhibition as follows:

$$\% \text{ Relative Inhibition} = \frac{(Slope\ of\ EC - Slope\ of\ S)}{Slope\ of\ EC} \times 100$$

Technical Support

Copyright © 2023 Abcam. All Rights Reserved. The Abcam logo is a registered trademark. All information / detail is correct at time of going to print.
For all technical or commercial enquiries please go to:

www.abcam.com/contactus
www.abcam.cn/contactus (China)
www.abcam.co.jp/contactus (Japan)

Supplementary information 2

ab212166 – Human BDNF SimpleStep ELISA® Kit

For the quantitative measurement of free BDNF in human serum, plasma (heparin), plasma (EDTA), plasma (citrate), cell culture supernatant, and CSF.
For research use only - not intended for diagnostic use.

For overview, typical data and additional information please visit: www.abcam.com/ab212166

Storage and Stability: Store kit at 2-8°C immediately upon receipt. Refer to list of materials supplied for storage conditions of individual components. Observe the storage conditions for individual prepared components in the Standard Preparation and Reagent preparation sections.

Limitations: All data, except Typical Standard Curve and Sensitivity, were collected using the colorimetric version of this kit (ab212166).

Materials Supplied

Item	Quantity	Storage Condition
Human BDNF Capture Antibody 10X	600 µL	+4°C
Human BDNF Detector Antibody 10X	600 µL	+4°C
Human BDNF Lyophilized Recombinant Protein	2 Vials	+4°C
Antibody Diluent CPI2	6 mL	+4°C
Sample Diluent NS	50 mL	+4°C
Wash Buffer PT 10X	20 mL	+4°C
TMB Development Solution	12 mL	+4°C
Stop Solution	12 mL	+4°C
SimpleStep Pre-Coated 96-Well Microplate	96 Wells	+4°C
Plate Seal	1	+4°C

Note: Antibody Diluent CPI2- This buffer has been reformulated to enhance stability after freeze-thaw cycles while producing data equivalent to the original formulation of antibody diluent CPI previously used in this kit.
While we run stock down, you may receive kits containing antibody diluent CPI. This does not affect the way you should use the kit.
If you have any questions please contact Abcam Scientific Support.

Materials Required, Not Supplied

These materials are not included in the kit, but will be required to successfully utilize this assay:

Microplate reader capable of measuring absorbance at 450 or 600 nm.
Deionized water.
Multi- and single-channel pipettes.
Tubes for standard dilution.
Plate shaker for all incubation steps.
Optional: Phenylmethylsulfonyl Fluoride (PMSF) (or other protease inhibitors).

Reagent Preparation

Equilibrate all reagents to room temperature (18-25°C) prior to use. The kit contains enough reagents for 96 wells. The sample volumes below are sufficient for 48 wells (6 x 8-well strips); adjust volumes as needed for the number of strips in your experiment.

Prepare only as much reagent as is needed on the day of the experiment. Capture and Detector Antibodies have only been tested for stability in the provided 10X formulations

1X Wash Buffer PT: Prepare 1X Wash Buffer PT by diluting Wash Buffer PT 10X with deionized water. To make 50 mL 1X Wash Buffer PT combine 5 mL Wash Buffer PT 10X with 45 mL deionized water. Mix thoroughly and gently.

Antibody Cocktail: Prepare Antibody Cocktail by diluting the capture and detector antibodies in Antibody Diluent CPI2. To make 3 mL of the Antibody Cocktail combine 300 µL 10X Capture Antibody and 300 µL 10X Detector Antibody with 2.4 mL Antibody Diluent CPI2. Mix thoroughly and gently.

Standard Preparation

Always prepare a fresh set of standards for every use. Discard working standard dilutions after use as they do not store well. The following section describes the preparation of a standard curve for duplicate measurements (recommended).

1. Reconstitute the BDNF standard sample by adding the volume of Sample Diluent NS indicated on the protein vial label. Hold at room temperature for 10 minutes. Mix thoroughly and gently. This is the 2,000 pg/mL **Stock Standard** Solution.
2. Label 8 tubes, Standards 1– 8.
3. Add 150 µL of Sample Diluent NS into tube number 1-8.
4. Use the Stock Standard to prepare the following dilution series. Standard #8 contains no protein and is the Blank control.

Standard #	Dilution Sample	Volume to Dilute (µL)	Volume of Diluent (µL)	Starting Conc. (pg/mL)	Final Conc. (pg/mL)
1	**Stock Standard**	150	150	2,000	1,000
2	Standard#1	150	150	1,000	500
3	Standard#2	150	150	500	250
4	Standard#3	150	150	250	125
5	Standard#4	150	150	125	62.5
6	Standard#5	150	150	62.5	31.3
7	Standard#6	150	150	31.3	15.6
8	Blank Control	0	150	0	0

Sample Preparation

Typical Sample Dynamic Range	
**Sample Type**	**Range**
Human Serum	1 - 10%
Human Plasma - Heparin	1 - 10%
Human Plasma - EDTA	1 - 10%
Human Plasma - Citrate	1 - 10%
Rat Serum	1.25 - 20%
L929 Stimulated Supernatant*	≤ 50%
Human Cerebrospinal Fluid*	≤ 50%

*Based on spiked sample

Serum Samples should be collected into a serum separator tube. After clot formation, centrifuge samples at 2,000 x g for 10 minutes and collect serum. Dilute samples at least 1:10 into Sample Diluent NS and assay. Store un-diluted serum at -20°C or below. Avoid repeated freeze-thaw cycles.
Plasma Collect plasma using citrate, EDTA or heparin. Centrifuge samples at 2,000 x g for 10 minutes. Dilute samples at least 1:10 into Sample Diluent NS and assay. Store un-diluted plasma samples at -20°C or below for up to 3 months. Avoid repeated freeze-thaw cycles.
Cell Culture Supernatants Centrifuge cell culture media at 2,000 x g for 10 minutes to remove debris. Collect supernatants and assay. Or dilute samples at least 1:2 into Sample Diluent NS and assay. Store un-diluted samples at -20°C or below. Avoid repeated freeze-thaw cycles.
Cerebrospinal Fluid (CSF) Dilute cerebrospinal fluid at least 1:2 into Sample Diluent NS and assay. Store un-diluted samples at -20°C or below. Avoid repeated freeze-thaw cycles.

Plate Preparation

The 96 well plate strips included with this kit are supplied ready to use. It is not necessary to rinse the plate prior to adding reagents.
Unused plate strips should be immediately returned to the foil pouch containing the desiccant pack, resealed and stored at 4°C.
For each assay performed, a minimum of two wells must be used as the zero control.
For statistical reasons, we recommend each sample should be assayed with a minimum of two replicates (duplicates).
Differences in well absorbance or "edge effects" have not been observed with this assay.

Assay Procedure

Equilibrate all materials and prepared reagents to room temperature prior to use.
We recommend that you assay all standards, controls and samples in duplicate

1. Prepare all reagents, working standards, and samples as directed in the previous sections.
2. Remove excess microplate strips from the plate frame, return them to the foil pouch containing the desiccant pack, reseal and return to 4°C storage.
3. Add 50 µL of all sample or standard to appropriate wells.
4. Add 50 µL of the Antibody Cocktail to each well.
5. Seal the plate and incubate for 1 hour at room temperature on a plate shaker set to 400 rpm.
6. Wash each well with 3 x 350 µL 1X Wash Buffer PT. Wash by aspirating or decanting from wells then dispensing 350 µL 1X Wash Buffer PT into each well. Wash Buffer PT should remain in wells for at least 10 seconds. Complete removal of liquid at each step is essential for good performance. After the last wash invert the plate and tap gently against clean paper towels to remove excess liquid.
7. Add 100 µL of TMB Development Solution to each well and incubate for 10 minutes in the dark on a plate shaker set to 400 rpm.
   Given variability in laboratory environmental conditions, optimal incubation time may vary between 5 and 20 minutes.
   Note: The addition of Stop Solution will change the color from blue to yellow and enhance the signal intensity about 3X. To avoid signal saturation, proceed to the next step before the high concentration of the standard reaches a blue color of O.D.600 equal to 1.0.
8. Add 100 µL of Stop Solution to each well. Shake plate on a plate shaker for 1 minute to mix. Record the OD at 450 nm. This is an endpoint reading.
9. Alternative to 7 – 8: Instead of the endpoint reading at 450 nm, record the development of TMB Substrate kinetically. Immediately after addition of TMB Development Solution begin recording the blue color development with elapsed time in the microplate reader prepared with the following settings:

Mode	Kinetic
Wavelength:	600 nm
Time:	up to 20 min
Interval:	20 sec - 1 min
Shaking:	Shake between readings

Note that an endpoint reading can also be recorded at the completion of the kinetic read by adding 100 µL Stop Solution to each well and recording the OD at 450 nm.

Download our ELISA guide for technical hints, results, calculation, and troubleshooting tips:
www.abcam.com/protocols/the-complete-elisa-guide

ab212166 – Human BDNF ® SimpleStep ELISA® Kit

Additional information

ASSAY SPECIFICITY
This kit is designed for the quantification of free human BDNF.

The standard protein in this kit is mature full length human BDNF.

Native signal was detected in serum, plasma (heparin), plasma (EDTA), plasma (citrate), and cell culture supernatant.

Spiked protein experiments were used to validate cell culture supernatant and CSF sample types.

Milk, saliva, urine, cell extract, and tissue extract samples have not been tested with this kit.

CROSS REACTIVITY
50 ng/mL and 500 pg/mL of recombinant human TRKB was tested for cross reactivity. No cross reactivity was observed.

Cross reactivity against the precursor form of BDNF has not been tested.

INTERFERENCE
50 ng/mL and 1 ng/mL of recombinant human TRKB was tested for interference with 200 pg/mL of recombinant human BDNF. 50 ng/mL of recombinant human TRKB decreased interpolated value of 200 pg/mL of recombinant human BDNF by 76%. 1 ng/mL of recombinant human TRKB decreased interpolated value of 200 pg/mL of recombinant human BDNF by 39%.

SPECIES REACTIVITY
This kit recognizes human and rat free BDNF protein.

Other species reactivity was determined by measuring 10% serum samples of various species, interpolating the BDNF protein concentrations from the human standard curve, and expressing the interpolated concentrations as a percentage of the BDNF protein concentration in human serum assayed at the same dilution.

7% cross-reactivity was observed in pooled normal bovine and mouse serum. 226% cross-reactivity was observed in pooled rat serum.

Other species reactivity not determined.

CALCULATION
- Preconfigured protocols are available when using SoftMax Pro software from Molecular Devices
- Calculate the average fluorescence value for the blank control (zero) standards. Subtract the average blank control standard fluorescence value from all other fluorescence values.
- Create a standard curve by plotting the average blank control subtracted fluorescence value for each standard concentration (y-axis) against the target protein concentration (x-axis) of the standard. Use graphing software to draw the best smooth curve through these points to construct the standard curve.
- Note: Most fluorescence reader software or graphing software will plot these values and fit a curve to the data. A four-parameter curve fit (4PL) is often the best choice; however, other algorithms (e.g. linear, semi-log, log/log, 4-parameter logistic) can also be tested to determine if it provides a better curve fit to the standard values.
- Determine the concentration of the target protein in the sample by interpolating the blank control subtracted fluorescence values against the standard curve. Multiply the resulting value by the appropriate sample dilution factor, if used, to obtain the concentration of target protein in the sample.
- Samples generating fluorescence values greater than that of the highest standard should be further diluted and reanalyzed. Similarly, samples which measure at fluorescence values less than that of the lowest standard should be retested in a less dilute form.

TYPICAL DATA
Typical standard curve – data provided for demonstration purposes only. A new standard curve must be generated for each assay performed

Standard Curve Measurements			
Concentration (pg/mL)	O.D 450 nm		Mean O.D
	1	2	
0	0.082	0.081	0.081
15.6	0.149	0.139	0.144
31.3	0.209	0.203	0.206
62.5	0.330	0.284	0.307
125	0.557	0.566	0.561
250	1.077	1.067	1.072
500	2.022	2.089	2.055
1,000	3.294	3.282	3.288

Table 1. Example of human BDNF standard curve in Sample Diluent NS. The BDNF standard curve was prepared as described in the Standard Preparation section. Raw data values are shown in the table.

TYPICAL SAMPLE VALUES

Sensitivity:

The calculated minimal detectable dose (MDD) is 2.4 pg/mL. The MDD was determined by calculating the mean of zero standard replicates (n=33) and adding 2 standard deviations then extrapolating the corresponding concentration.

Recovery

Three concentrations of BDNF were spiked in duplicate to the indicated biological matrix to evaluate signal recovery in the working range of the assay.

Sample Type	Average % Recovery	Range (%)
10% Human Serum	97	94 - 104
5% Human Plasma - Citrate	103	95 - 111
25% Human Plasma - EDTA	98	85 - 108
5% Human Plasma - Heparin	103	96 - 112
2.5% Rat Serum	107	103 - 110
50% Cell Culture Supernatant	106	103 - 109
50% Human Cerebrospinal Fluid	95	88 - 104

Linearity of Dilution

Linearity of dilution is determined based on interpolated values from the standard curve. Linearity of dilution defines a sample concentration interval in which interpolated target concentrations are directly proportional to sample dilution.

Native BDNF was measured in the following biological samples in a 2-fold dilution series. Sample dilutions are made in Sample Diluent NS.

Dilution Factor	Interpolated value	10% Human Serum	10% Human Plasma (Citrate)	10% Human Plasma (EDTA)	10% Human Plasma (Heparin)	20% Rat Serum
Undiluted	pg/mL	93.5	207.6	70.2	90.4	423.0
	% Expected value	100	100	100	100	100
2	pg/mL	51.0	104.4	35.2	41.3	238.3
	% Expected value	109	101	100	91	113
4	pg/mL	24.4	56.0	17.1	21.5	123.2
	% Expected value	104	108	97	95	117
8	pg/mL	11.1	29.4	8.4	10.4	59.9
	% Expected value	95	113	96	92	113
16	pg/mL	ND	15.3	ND	ND	30.4
	% Expected value	ND	118	ND	ND	115

ND – Not Detectable

Recombinant BDNF was spiked in in the following biological samples in a 2-fold dilution series. Sample dilutions are made in Sample Diluent NS.

Dilution Factor	Interpolated value	50% Cell Culture Media	50% L929 Stimulated Supernatant	50% Human CSF
Undiluted	pg/mL	529.2	573.4	498.3
	% Expected value	100	100	100
2	pg/mL	248.8	247.9	265.5
	% Expected value	94	86	107
4	pg/mL	128.3	128.8	132.3
	% Expected value	97	90	106
8	pg/mL	63.5	61.6	65.1
	% Expected value	96	86	104
16	pg/mL	32.9	32.2	28.1
	% Expected value	99	90	90

Precision

Mean coefficient of variations of interpolated values of BDNF from three concentrations of human serum within the working range of the assay.

	Intra-assay	Inter-assay
N=	5	3
CV (%)	2.8	5.3

Download our ELISA guide for technical hints, results, calculation, and troubleshooting tips:
www.abcam.com/protocols/the-complete-elisa-guide

Technical Support

Copyright © 2023 Abcam. All Rights Reserved. The Abcam logo is a registered trademark. All information / detail is correct at time of going to print.

For all technical or commercial enquiries please go to:
www.abcam.com/contactus
www.abcam.cn/contactus (China)
www.abcam.co.jp/contactus (Japan)

Supplementary information 3

（FOR RESEARCH USE ONLY. DO NOT USE IT IN CLINICAL DIAGNOSIS !）

Elabscience® Total Antioxidant Status (TAS) Colorimetric Assay Kit

Catalog No：E-BC-K801-M

Specification：48T(32 samples)/96T(80 samples)

Measuring instrument：Microplate reader（650-670 nm）

Detection range：0.23-2 mmol Trolox Equiv. /L

This manual must be read attentively and completely before using this product.
If you have any problem, please contact our Technical Service Center for help：

Phone: 240-252-7368(USA)
Fax: 240-252-7376(USA)
Email: techsupport@elabscience.com
Website: www.elabscience.com

Please kindly provide us the lot number (on the outside of the box) of the kit for more efficient service.

Table of contents

Assay summary 3

Intended use 4

Detection principle 4

Kit components & storage 4

Materials prepared by users 5

Reagent preparation 5

Sample preparation 6

The key points of the assay 7

Operating steps 7

Calculation 8

Appendix I Performance Characteristics 9

Appendix Π Example Analysis 11

Statement 12

Assay summary

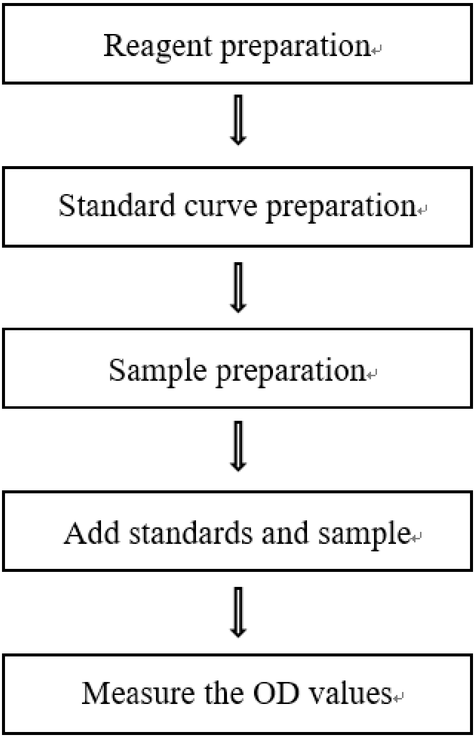

Intended use

The kit is used for the determination of total antioxidant status (TAS) in serum, plasma, urine, cellular supernatant, animal and plant tissue samples.

Detection principle

ABTS is oxidized to green ABTS•+ by appropriate oxidant, which can be reduced to colorless ABTS in the presence of antioxidants. The TAS of the sample can be determined and calculated by measuring the absorbance of $ABTS^+$ at 660 nm. Trolox is an analog of VE and has a similar antioxidant state to that of VE. Trolox is used as a reference substance for total antioxidant status.

Kit components & storage

Item	Component	Size 1(48 T)	Size 2(96 T)	Storage
Reagent 1	Buffer Solution	15 mL ×1 vial	30 mL ×1 vial	-20℃, 12 months
Reagent 2	Chromogenic Agent	5 mL ×1 vial	5 mL ×1 vial	-20℃, 12 months, shading light
Reagent 3	2 mmol/L Standard	2 mL ×1 vial	4 mL ×1 vial	-20℃, 12 months, shading light
	Microplate	96 wells		No requirement
	Plate Sealer	2 pieces		

Note: The reagents must be stored strictly according to the preservation conditions in the above table. The reagents in different kits cannot be mixed with each other. For a small volume of reagents, please centrifuge before use, so as not to obtain sufficient amount of reagents.

Materials prepared by users

Instruments:

Microplate reader (650-670 nm, optimum wavelength: 660 nm), Micropipettor, 37℃ incubator

Reagents:

Double distilled water, 60% Ethanol

Reagent preparation

① Equilibrate all reagents to room temperature before use.

② The preparation of standard curve：

Always prepare a fresh set of standards. Discard working standard dilutions after use.

Dilute 2 mmol/L standard solution with 60% ethanol to a serial concentration. The recommended dilution gradient is as follows: 0, 0.4, 0.8, 1.2, 1.4, 1.6, 1.8, 2 mmol/L. Reference is as follows:

Item	①	②	③	④	⑤	⑥	⑦	⑧
**Concentration (mmol/L)**	**0**	**0.4**	**0.8**	**1.2**	**1.4**	**1.6**	**1.8**	**2**
**2 mmol/L standard (μL)**	0	40	80	120	140	160	180	200
**60% ethanol (μL)**	200	160	120	80	60	40	20	0

Sample preparation

① Sample preparation

Serum and plasma: detect directly. If not detected on the same day, the serum or plasma can be stored at -80℃ for a month.

Tissue sample:

① Harvest the amount of tissue needed for each assay (initial recommendation 20 mg).

② Wash tissue in cold PBS (0.01 M, pH 7.4).

③ Homogenize 20 mg tissue in 180 60% ethanol with a dounce homogenizer at 4℃.

④ Centrifuge at 10000×g for 10 minutes at 4℃ to remove insoluble material. Collect supernatant and keep it on ice for detection.

② Dilution of sample

The recommended dilution factor for different samples is as follows (for reference only):

Sample type	Dilution factor
10% Mouse liver tissue homogenate	1
10% Rat liver tissue homogenate	1
10% Rat lung tissue homogenate	1
Molt4 cellar supernatant	1
Human urine	8-10
Mouse serum	1
Human serum	1
Human saliva	1

Note: The diluent is 60% Ethanol. For the dilution of other sample types, please do pretest to confirm the dilution factor

The key points of the assay

① When adding chromogenic agent, suck and beat with micropipettor repeatedly to ensure the color system mix fully.

② Avoid bubbles when adding samples.

Operating steps

① Standard well: Add 10 μL of standard with different concentration to the standard well.

Sample well: Add 10 μL of sample to the sample well.

② Add 200 μL of buffer solution to each well.

③ Measure the OD values of each well at 660 nm with microplate reader, recorded as A_1.

④ Add 20 μL of chromogenic agent to each well, repeatedly suck and beat for 5-6 times.

⑤ Incubate at 37 ℃ for 5 min. Measure the OD values of each well at 660 nm with microplate reader, recorded as A_2. $\Delta A = A_2 - A_1$.

Calculation

The standard curve:

1. Average the duplicate reading for each standard.

2. Subtract the mean OD value of the blank (Standard #①) from all standard readings. This is the absoluted OD value.

3. Plot the standard curve by using absoluted OD value of standard and correspondent concentration as y-axis and x-axis respectively. Create the standard curve (**y = ax + b**) with graph software (or EXCEL).

The sample:

1. Liquid sample (Trolox is used as a reference substance for total antioxidant status):

$$\text{TAS (mmol Trolox Equiv. /L)} = (\Delta A_{Blank} - \Delta A_{Sample} - b) \div a \times f$$

2. Tissue sample:

$$\text{TAS (mmol Trolox Equiv. /kg wet weight)} = (\Delta A_{Blank} - \Delta A_{Sample} - b) \div a \div (m \div v) \times f$$

[Note]

y: ΔA_{Blank} $-\Delta A_{Standard}$ (ΔA_{Blank} is ΔA when the standard concentration is 0).

x: The concentration of Standard.

a: The slope of standard curve.

b: The intercept of standard curve.

ΔA_{Sample}: The OD value of sample (A_2 - A_1).

m：The weight of tissue sample (g).

V：The volume of added homogenate (mL).

f: Dilution factor of sample before test.

Appendix I Performance Characteristics

1. Parameter:

Intra-assay Precision

Three human serum samples were assayed in replicates of 20 to determine precision within an assay (CV = Coefficient of Variation).

Parameters	Sample 1	Sample 2	Sample 3
**Mean (mmol Trolox Equiv./L)**	0.87	1.05	1.46
**%CV**	4.8	4.5	4.5

Inter-assay Precision

Three human serum samples were assayed 20 times in duplicate by three operators to determine precision between assays.

Parameters	Sample 1	Sample 2	Sample 3
**Mean (mmol Trolox Equiv./L)**	0.87	1.05	1.46
**%CV**	6.8	7.3	6.9

Recovery

Take three samples of high concentration, middle concentration and low concentration to test the samples of each concentration for 6 times parallelly to get the average recovery rate of 99%.

	Standard 1	Standard 2	Standard 3
**Expected Conc. （mmol/L）**	0.7	1.35	1.75
**Observed Conc. （mmol/L）**	0.7	1.3	1.7
**recovery rate(%)**	101	99	97

Sensitivity

The analytical sensitivity of the assay is 0.23 mmol Trolox Equiv./L. This was determined by adding two standard deviations to the mean O.D. obtained when the zero standard was assayed 20 times, and calculating the corresponding concentration.

2. Standard curve:

As the OD value of the standard curve may vary according to the conditions of the actual assay performance (e.g. operator, pipetting technique or temperature effects), so the standard curve and data are provided as below for reference only：

**Concentration (mmol/L)**	**0**	**0.4**	**0.8**	**1.2**	**1.4**	**1.6**	**1.8**	**2**
**Average OD**	1.196	0.978	0.818	0.664	0.583	0.516	0.436	0.368
**Absoluted OD**	0.000	0.217	0.378	0.532	0.612	0.680	0.759	0.827

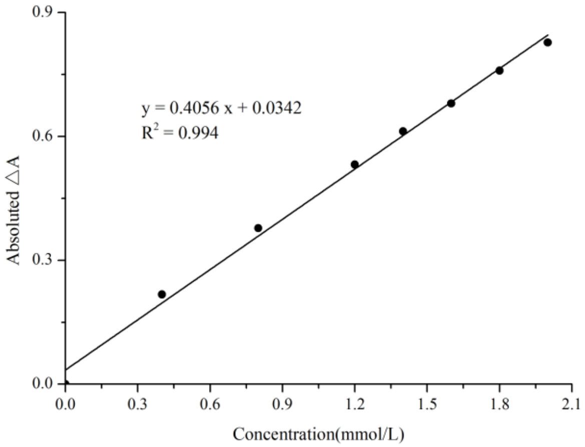

Appendix Π Example Analysis

Example analysis:

For human serum, take human serum sample and carry the assay according to the operation steps. The results are as follows:

Standard curve: y = 0.4056 x + 0.0342, the OD value of the sample (A_1) is 0.08, the OD value of the sample (A_2) is 0.777, $\Delta A_{Sample} = A_2 - A_1$ =0.697, ΔA_{Blank} is 1.196, and the calculation result is:

TAS (mmol Trolox Equiv. /L) = (1.196–0.697 -0.0342) ÷0.4056 =1.14 mmol Trolox Equiv. /L

Detect 10% Mouse kidney tissue homogenate (dilute for 4 times), human serum, mouse serum and porcine serum according to the protocol, the result is as follows:

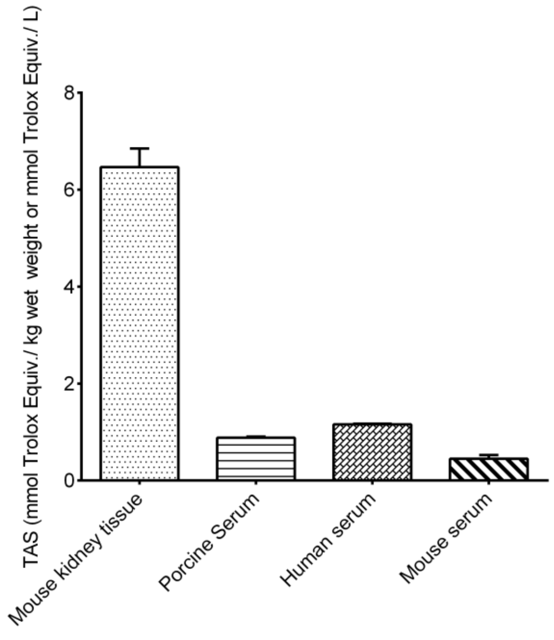

Statement

1. This assay kit is for Research Use Only. We will not response for any arising problems or legal responsibilities causing by using the kit for clinical diagnosis or other purpose.
2. Please read the instructions carefully and adjust the instruments before the experiments. Please follow the instructions strictly during the experiments.
3. Protection methods must be taken by wearing lab coat and latex gloves.
4. If the concentration of substance is not within the detection range exactly, an extra dilution or concentration should be taken for the sample.
5. It is recommended to take a pre-test if your sample is not listed in the instruction book.
6. The experimental results are closely related to the situation of reagents, operations, environment and so on. Elabscience will guarantee the quality of the kits only, and NOT be responsible for the sample consumption caused by using the assay kits. It is better to calculate the possible usage of sample and reserve sufficient samples before use.

For life science research only.
Not for use in diagnostic procedures.

Roche

Cytotoxicity Detection Kit (LDH)

Version: 12
Content Version: November 2020

A nonradioactive alternative to the [^{3}H]-thymidine- and [^{51}Cr]-release assays.
Colorimetric assay for the quantification of cell death and cell lysis, based on the measurement of lactate dehydrogenase (LDH) activity released from the cytosol of damaged cells into the supernatant.

Cat. No. 11 644 793 001 1 kit
2,000 tests

Store the kit at −15 to −25°C.

1. General Information ... 3
1.1. Contents ... 3
1.2. Storage and Stability ... 3
Storage Conditions (Product) ... 3
1.3. Additional Equipment and Reagent required ... 3
1.4. Application ... 4
1.5. Preparation Time ... 4
Assay Time ... 4
2. How to Use this Product ... 5
2.1. Before you Begin ... 5
Sample Materials ... 5
Control Reactions ... 5
Background control ... 5
Low control ... 5
High control ... 5
Overview of the controls ... 6
Calculation with the controls ... 6
General Considerations ... 6
Potential sources of test interference ... 6
Safety Information ... 7
For customers in the European Economic Area ... 7
Laboratory procedures ... 7
Waste handling ... 7
Working Solution ... 7
2.2. Protocols ... 8
Determination of the optimal cell concentration for the assay ... 8
Protocol for a 96-well microplate ... 8
Measurement of the cytotoxic potential of soluble substancesProtocol for a 96-well microplate ... 10
Measurement of cell-mediated cytotoxicitySample arrangement on a 96-well microplate ... 11
Protocol for a 96-well microplate ... 12
Measurement of cell death in eukaryotic cell fermentation ... 13
2.3. Parameters ... 14
Sensitivity ... 14
3. Troubleshooting ... 15
4. Additional Information on this Product ... 16
4.1. Test Principle ... 16
How this product works ... 17
5. Supplementary Information ... 18
5.1. Conventions ... 18
5.2. Changes to previous version ... 18
5.3. Trademarks ... 19
5.4. License Disclaimer ... 19
5.5. Regulatory Disclaimer ... 19
5.6. Safety Data Sheet ... 19
5.7. Contact and Support ... 19

1. General Information

1.1. Contents

Vial / Bottle	Cap	Label	Function / Description	Content
1	blue	Cytotoxicity Detection Kit, Catalyst	▪ Lyophilized, stabilized<br>▪ Catalyst for reaction mix.<br>▪ Diaphorase/NAD^+ mixture.	5 bottles
2	red	Cytotoxicity Detection Kit, Dye solution	▪ Ready-to-use solution.<br>▪ Contains iodotetrazolium chloride (INT) and sodium lactate.<br>▪ Dyes the reaction mix.	5 bottles, 45 ml each

1.2. Storage and Stability

Storage Conditions (Product)

The kit is shipped on dry ice.
When stored at −15 to −25°C, the kit is stable through the expiration date printed on the label.

Vial / Bottle	Cap	Label	Storage
1	blue	Catalyst	Store at −15 to −25°C.
2	red	Dye solution	

1.3. Additional Equipment and Reagent required

Standard laboratory equipment

- +37°C incubator
- Centrifuge with rotor for microplates
- ELISA reader for microplates, with 490 to 492 nm filter
  If a reference wavelength will be subtracted, use a filter >600 nm.
- Microscope
- Hemocytometer
- Multichannel pipettor (100 µl)
- Sterile pipette tips
- 96-well microplates:
  – For the measurement of cell-mediated lysis and for the analysis of cytotoxic compounds, use sterile, cell-culture quality with round or V-bottom for suspension cells, or with flat bottom for adherent cells.
  – For color development in all assays, use optically clear, flat-bottomed microplates.

Standard laboratory reagents

- Double-distilled water
- Assay medium, such as medium containing 1% serum or 1% bovine serum albumin.
  – Both human and animal sera contain various amounts of LDH which may increase background absorbance in the assay. Therefore, to increase the sensitivity, perform the assay in the presence of low serum concentrations, such as 1% or replace serum with 1% bovine serum albumin (BSA) (w/v).
- Triton X-100 solution* (2% Triton X-100 in assay medium)
  – The maximum amount of releasable LDH enzyme activity is determined by lysing the cells with Triton X-100 (final concentration: 1% Triton X-100). At this concentration, Triton X-100 does not affect the LDH activity.
- HCl stop solution (1 N)
  ℹ *The reaction product can be measured without addition of a stop solution. Alternatively, the enzyme reaction can be stopped by the addition of 50 µl/well 1N HCl (final concentration: 0.2 N HCl).*

For LDH standard preparation

- LDH standard solution, such as 0.05 U LDH/ml, see section **Controls.**
  ℹ *If the released LDH activity is calculated in U/ml instead of percent relative cytotoxicity or absorbance, use an appropriate LDH preparation as standard.*

⚠ ***Assay medium, lysing and stopping solutions, as well as LDH standard are not included in the kit; all other reagents necessary to perform 2,000 tests are included.***

1.4. Application

The Cytotoxicity Detection Kit is a precise, fast, and simple colorimetric assay for quantitating cytotoxicity and cytolysis by measuring LDH activity released from damaged cells. Therefore, the kit can be used in many different *in vitro* cell systems when damage to the plasma membrane occurs.

- Determination of the cytotoxic potential of compounds in environmental and medical research, and in the food, cosmetic, and pharmaceutical industries.
- Determination of mediator-induced cytolysis.
- Detection and quantification of cell-mediated cytotoxicity induced by cytotoxic T-lymphocytes (CTL), natural killer (NK) cells, lymphokine activated killer (LAK) cells, or monocytes.
  ℹ *The LDH release assay and the [^{51}Cr] release assay show good correlation when used to monitor cell-mediated cytotoxicity in a variety of murine and human effector-target cell systems, including NK cells, CTL, and macrophages.*
- Measurement of antibody-dependent cellular cytotoxicity (ADCC) and complement-mediated cytolysis.
- Determination of cell death in bioreactors.
  ℹ *Experiments have shown that measurement of the release of cytoplasmic LDH enzyme activity to the culture medium can provide a precise evaluation of cell death during fermentation in bioreactors.*
- The assay can also be used to determine the total numbers of cells present at the end of a proliferation assay.

1.5. Preparation Time

Assay Time

0.5 to 1 hour, including harvesting of the supernatants and substrate reaction.

2. How to Use this Product

2.1. Before you Begin

Sample Materials

The Cytotoxicity Detection Kit (LDH) is used with cell-free supernatants obtained from cells cultured in 96-well microplates or batch cultures. The assay reagent is not harmful to the cells and can be added directly to the cell culture plate. Alternatively, when the samples are not tested directly, remove the cells from the culture medium prior to the determination of LDH activity by centrifugation at approximately 250 × *g*.
⚠ ***Store the cell-free culture supernatant at +2 to +8°C for several days without loss of LDH activity.***

Control Reactions

To calculate percent cytotoxicity, use the following three controls in each experimental setup.

Background control

Determines the LDH activity contained in the assay medium.
⚠ ***The absorbance value obtained in this control must be subtracted from all other values.***

Low control

Determines the LDH activity released from the untreated normal cells (= spontaneous LDH release).

High control

Determines the maximum releasable LDH activity in the cells (= maximum LDH release).

The following two controls are facultative:

Substance control I

Determines the LDH activity contained in the test substance. If cell-mediated cytotoxicity is measured, this control provides information about the LDH activity released from the effector cells (= effector cell control, see **Figure 5, section Protocols**).

Substance control II

Determines whether the test substance itself interferes with LDH activity.
To perform this control:

1. To each control sample (assayed in triplicate) in an optically clear, 96-well, flat-bottom microplate, add 50 µl assay medium containing the test substance.

2. Add 50 µl/well LDH standard solution (0.05 U/ml).

3. Add 100 µl/well Reaction mixture and measure absorbance using an ELISA reader as described in the Protocols below. This is related to the procedure used in section, **Determination of the optimal cell concentration for the assay**, and the other assays in the protocol section.

4. Compare the absorbance values in these controls with absorbance values obtained in separate (triplicate) LDH control samples that contain only 50 µl/well assay medium, 50 µl/well LDH standard solution (0.05 U/ml), and 100 µl/well Reaction mixture.

Overview of the controls

⚠ ***The background, low, and high controls must be determined in each experimental setup.***

Contents of the Well	Background Control [µl]	Low Control [µl]	High Control [µl]	Substance Control 1 [µl]	Substance Control II [µl]	Experimental Sample [µl]
Cell-free culture medium	200	100	–	100	–	–
Cells	–	100	100	–	–	100
Triton X-100 solution (2% in assay medium)	–	–	100	–	–	–
Test substance or effector cells diluted in culture medium	–	–	–	100	50	100
LDH standard solution	–	–	–	–	50	–

Calculation with the controls

To determine the percentage cytotoxicity, calculate the average absorbance values of the triplicate samples and controls, subtract the background from each, then substitute the resulting values in the following equation:

$$\text{Cytotoxicity (\%)} = \frac{\text{exp. value - low control}}{\text{high control - low control}} \times 100$$

Fig. 1: Percent cytotoxicity formula.
To determine the percentage cell-mediated cytotoxicity, calculate the average absorbance of the triplicate samples and controls, subtract the background from each, then substitute the resulting values in the following equation:

$$\text{Cytotoxicity (\%)} = \frac{\text{(effector - target cell mix - effector cell control) - low control}}{\text{high control - low control}} \times 100$$

Fig. 2: Percent cell-mediated cytotoxicity formula.

General Considerations

Potential sources of test interference

- Inherent LDH activity may be found in serum or test substances, see section **Controls**.
- In cell-mediated cytotoxicity assays, the amount of LDH released from damaged effector cells may influence the assay results, see sections **Controls** and **Measurement of cell-mediated cytotoxicity**.
- Substances which inhibit the LDH or diaphorase enzyme activity influence the assay. Include appropriate controls in the assay, see section **Controls**.
- Pyruvate is an inhibitor of the LDH reaction and is contained in some culture media, such as some formulations of DMEM, Ham's F12, or Iscove's.

Safety Information

For customers in the European Economic Area

Contains SVHC: octyl/nonylphenol ethoxylates. For use in research and under controlled conditions only – acc. to Art. 56.3 and 3.23 REACH Regulation.

Laboratory procedures

- Handle all samples as if potentially infectious, using safe laboratory procedures. As the sensitivity and titer of potential pathogens in the sample material varies, the operator must optimize pathogen inactivation by the Lysis/Binding Buffer or take appropriate measures, according to local safety regulations.
- Do not eat, drink, or smoke in the laboratory work area.
- Do not pipette by mouth.
- Wear protective disposable gloves, laboratory coats, and eye protection, when handling samples and kit reagents.
- Wash hands thoroughly after handling samples and reagents.

Waste handling

- Discard unused reagents and waste in accordance with country, federal, state, and local regulations.
- Safety Data Sheets (SDS) are available online on dialog.roche.com, or upon request from the local Roche office.

Working Solution

Content	Reconstitution/Preparation of Working Solution	Storage and Stability
Catalyst (Bottle 1)	Reconstitute the lyophilizate in 1 ml double-distilled water for 10 minutes; mix thoroughly.	▪ The lyophilizate is stable at +2 to +8°C.<br>▪ After reconstitution, store 4 weeks at +2 to +8°C.
Dye solution (Bottle 2)	Ready-to-use solution.	Once thawed, store several weeks at +2 to +8°C.
Reaction mixture	▪ For 100 tests, shortly before use, mix 250 µl of reconstituted Bottle 1 with 11.25 ml of Bottle 2.<br>▪ For 400 tests, shortly before use, add the total volume of Bottle 1 (1 ml) to the total volume of Bottle 2 (45 ml); mix thoroughly.	Always prepare fresh before use; do not store.

2.2. Protocols

Determination of the optimal cell concentration for the assay

Different cell types may contain different amounts of LDH, therefore, determine the optimal cell concentration for a specific cell type in a preliminary experiment (Fig. 3). In general, the optimal cell concentration is the one that produces the greatest difference between the Low and High control; use this concentration for the subsequent assay. For most cell lines, the optimal cell concentration is 0.5 to 2 × 10^4 cells/200 µl assay (= 0.25 to 1 × 10^5 cells/ml).

⚠ ***Perform all test samples in triplicate.***

Protocol for a 96-well microplate

1. Wash cells with assay medium.
   – Adjust cell suspension to a concentration of 2 × 10^6 cells/ml in assay medium.

---

2. Add 100 µl/well assay medium to each well of an entire 96-well, tissue-culture plate.

---

3. Use a multichannel pipette to prepare two-fold serial dilutions of the cells across the plate.
   – Prepare 6 wells of each dilution.
   ⚠ ***After dilution, the final volume in each well should be 100 µl. Leave at least 3 wells cell-free to use as a Background control.***
   – For each cell dilution, designate 3 wells as a Low control (= spontaneous LDH release) and 3 wells as a High control (= maximum LDH release).
   – For an overview of the controls, see section, **Controls**.

---

4. Incubate the cells in an incubator at +37°C, 5% CO_2, and 90% humidity,
   ⚠ ***Use the same incubation time that will be used in the final assay.***

---

5. Centrifuge the microplate at 250 × *g* for 10 minutes.
   ℹ *Optional step for adherent cells.*

---

6. Carefully remove 100 µl/well supernatant; do not disturb the cell pellet.
   – Transfer into corresponding wells of an optically clear, 96-well flat-bottom microplate.

---

7. To determine the LDH activity in these supernatants, add 100 µl freshly prepared Reaction mixture to each well, and incubate for up to 30 minutes at +15 to +25°C.
   ⚠ ***Protect the microplate from light during this incubation period.***

---

8. Measure the absorbance of the samples at 490 or 492 nm according to the available filters using an ELISA reader.

⚠ ***Use a reference wavelength of >600 nm.***

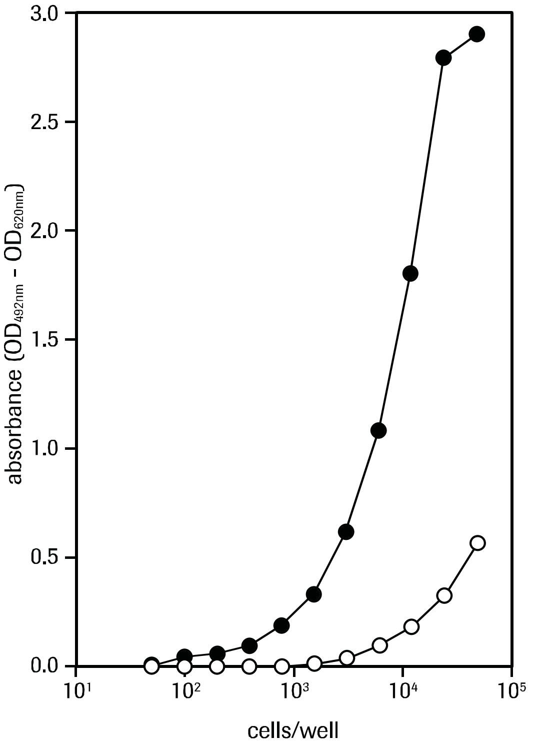

Fig. 3: Determination of the optimal target cell concentration for K-562 cells. K-562 cells were titrated in microplates as described above at cell concentrations indicated in the figure. Culture medium (○) was added for the determination of the spontaneous release of LDH activity and Triton X-100* (●) was added to a final concentration of 1% for the determination of maximal release of LDH activity. Optimal target cell concentration in this experiment is approximately 1 × 10^4 cells/well.

Measurement of the cytotoxic potential of soluble substances

Protocol for a 96-well microplate

⚠ ***Perform all test samples in triplicate.***

1. Wash the cells in assay medium, then dilute to the concentration determined in section, **Determination of the optimal cell concentration for the assay**.

---

2. For suspension cells:
   – Titrate test substances, such as mediators, cytolytic or cytotoxic agents in the appropriate assay medium in sterile, 96-well, tissue-culture plates by serial dilutions (final volume of 100 µl/well).
   – Add 100 µl/well cell suspension to the dilutions of the test substances.

---

3. For adherent cells (Fig. 4):
   – Add 100 µl cell suspension per well in a sterile, 96-well, tissue-culture plate.
   ⚠ ***Do not add cells to wells for Background control and Substance control I.***
   – Incubate the cells overnight in an incubator at +37°C, 5% CO_2, and 90% humidity to allow the cells to adhere tightly.
   – Immediately before use, titrate test substances, such as mediators, cytolytic or cytotoxic agents in the appropriate assay medium in a separate microplate by serial dilutions (final volume of 200 µl/well).
   – Remove the assay medium from the adherent cells to remove LDH activity released from the cells during the overnight incubation step, and add 100 µl fresh assay medium to each well.
   – Transfer 100 µl of the test substance dilutions into corresponding wells containing the adherent cells.

---

4. For an overview of the different controls, see section, **Controls**.
   – On the same plate, prepare the following controls in triplicate:

Controls	Add to each well
Background control	200 µl assay medium only.
Low control	100 µl cell suspension plus 100 µl assay medium.
High control	100 µl Triton X-100 solution plus 100 µl cells.
Substance control I	100 µl test substance at the maximum concentration used in the experiment plus 100 µl medium.

---

5. Incubate the cells in an incubator at +37°C, 5% CO_2, and 90% humidity.
   ⚠ ***Depending on the experimental setup, use incubation times of 2 to 24 hours.***

---

6. For suspension cells, centrifuge the microplate at 250 × *g* for 10 minutes.

---

7. Carefully remove 100 µl/well supernatant; do not disturb the cell pellet.
   – Transfer into corresponding wells of an optically clear, 96-well, flat-bottom microplate.

---

8. To determine the LDH activity in these supernatants, add 100 µl freshly prepared Reaction mixture to each well, and incubate for up to 30 minutes at +15 to +25°C.
   ⚠ ***Protect the microplate from light during this incubation period.***

---

9. Measure the absorbance of the samples at 490 or 492 nm according to the available filters using an ELISA reader.

⚠ ***Use a reference wavelength of >600 nm.***

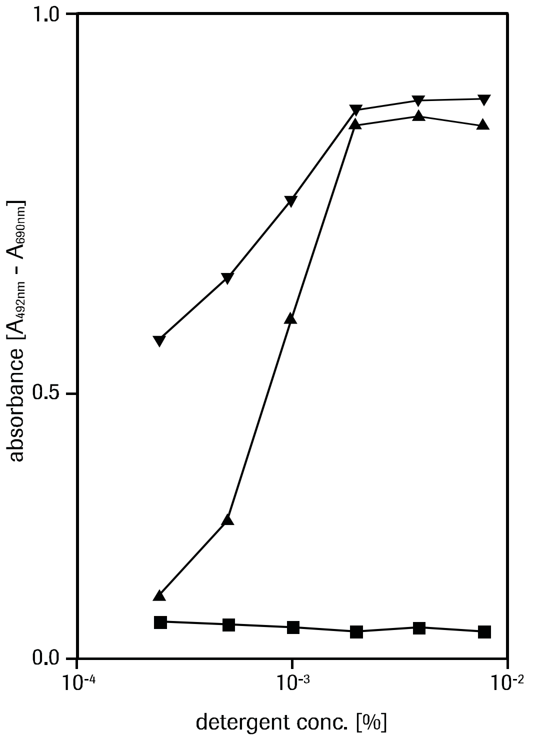

Fig. 4: Measurement of the cytotoxic potential of various detergents, such as Synperonic® F68 (■), Triton X-100* (▲), and Nonidet P40* (▼) were titrated in microplates in culture medium as described in section, **Measurement of the cytotoxic potential of soluble substances** to final concentrations indicated in the figure. Subsequently P815 cells were added to a final concentration of 1 × 10^4cells/well. The cells were incubated for 18 hours and LDH release was determined as described above.

Measurement of cell-mediated cytotoxicity

Sample arrangement on a 96-well microplate

⚠ ***Perform all test samples in triplicate.***

Background Control	Target Cell Low Control	Target Cell High Control	Blank
Effector – target cell mix ratio 1	Effector – target cell mix ratio 7	Effector cell control for mix ratio 1	Effector cell control for mix ratio 7
Effector – target cell mix ratio 2	Effector – target cell mix ratio 8	Effector cell control for mix ratio 2	Effector cell control for mix ratio 8
Effector – target cell mix ratio 3	Effector – target cell mix ratio 9	Effector cell control for mix ratio 3	Effector cell control for mix ratio 9
Effector – target cell mix ratio 4	Effector – target cell mix ratio 10	Effector cell control for mix ratio 4	Effector cell control for mix ratio 10
Effector – target cell mix ratio 5	Effector – target cell mix ratio 11	Effector cell control for mix ratio 5	Effector cell control for mix ratio 11
Effector – target cell mix ratio 6	Effector – target cell mix ratio 12	Effector cell control for mix ratio 6	Effector cell control for mix ratio 12

Protocol for a 96-well microplate

⚠ ***Perform all test samples in triplicate.***

1. Titrate effector cells, such as NK cells, LAK cells, and CTLs into the appropriate assay medium in sterile, 96-well, tissue-culture plates by serial dilutions (final volume of 100 µl/well).

2. Wash the target cells in assay medium; dilute to the concentration determined in the section, **Determination of the optimal cell concentration for the assay**.

3. Add 100 µl/well target cell suspension to the dilutions of effector cells (= effector-target cell mix), see **Sample arrangement on a 96-well microplate**.

4. For the the different controls, see section, **Controls**.
   On the same plate, prepare the following controls in triplicate:

Controls	Add to each well
Background control	200 µl assay medium only.
Low control (= spontaneous LDH release)	100 µl target cells plus 100 µl assay medium.
High control (= maximum LDH release)	100 µl target cells plus 100 µl Triton X-100 solution.
Substance control I (= effector cell control = spontaneous release of LDH by the effector cells)	100 µl assay medium plus 100 µl effector cells.

   ⚠ ***Always determine the spontaneous LDH release for each effector cell concentration used in the assay.***

5. Incubate the cells in an incubator at +37°C, 5% CO_2, and 90% humidity for the appropriate time period.

6. Centrifuge the microplate at 250 × *g* for 10 minutes.

7. Carefully remove 100 µl/well supernatant; do not disturb the cell pellet.
   – Transfer into corresponding wells of an optically clear, 96-well, flat-bottom microplate.

8. To determine the LDH activity in these supernatants, add 100 µl freshly prepared Reaction mixture to each well, and incubate for up to 30 minutes at +15 to +25°C.
   ⚠ ***Protect the microplate from light during this incubation period.***

9. Measure the absorbance of the samples at 490 or 492 nm according to the available filters using an ELISA reader (Fig. 5).

   ⚠ ***Use a reference wavelength of >600 nm.***

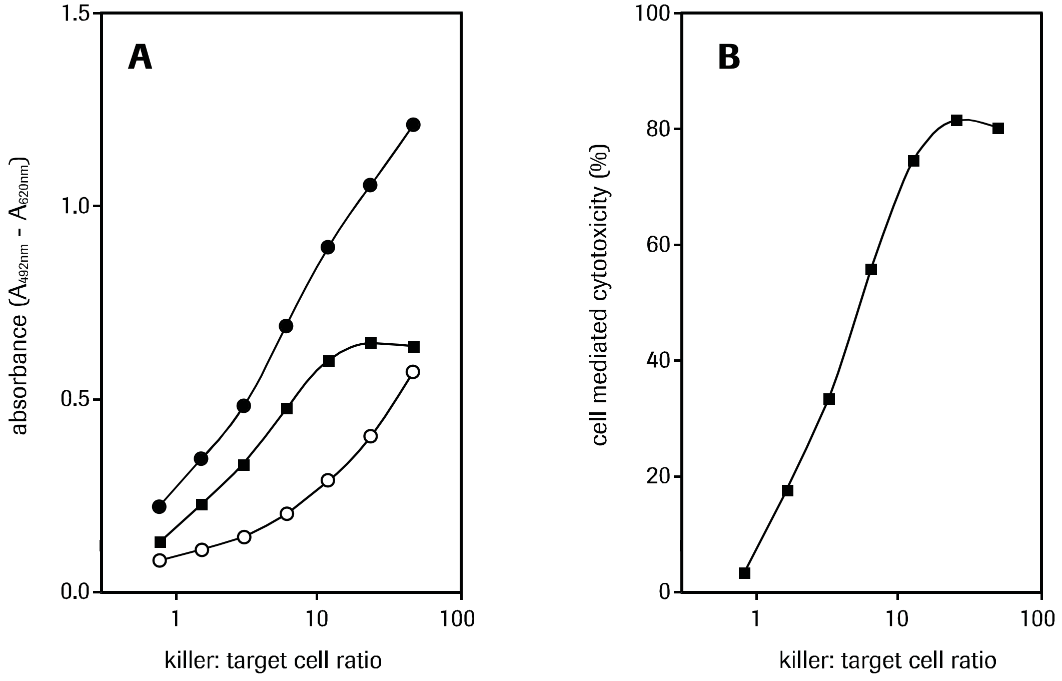

Fig. 5: Determination of the cytolytic activity of allogen-stimulated, cytotoxic T lymphocytes (CTLs). Spleen cells of C57/Bl 6 mice (H-2b) were stimulated *in vitro* with P815 cells (H-2d). Viable CTLs were purified by ficoll density gradient, washed and titrated in the microplate as described in section**, Measurement of cell mediated cytotoxicity**. 1 × 10^4 P815 target cells/well were added to the effector cells. The cells were centrifuged and incubated for 4 hours. 100 µl of culture supernatant was removed and LDH activity determined as described in section, **Measurement of cell mediated cytotoxicity**.
A. Absorbance values. Effector cell control (○), effector-target cell mix (●), effector-target cell mix minus effector cell control (■).
B. Percentage cell-mediated cytotoxicity, calculated as described in section, **Controls**.

Measurement of cell death in eukaryotic cell fermentation

1. Collect 0.5 to 1 ml samples at regular intervals of 12 or 24 hours from cell culture.

2. Spin the samples and carefully remove culture supernatant.
   Collect and store the cell-free supernatants at +2 to +8°C without loss of enzyme activity for several days.

3. Titrate the culture supernatants in the appropriate culture medium by serial dilutions to obtain a final volume of 100 µl/well.

4. Add 100 µl freshly prepared Reaction mixture to each well, and incubate for up to 30 minutes at +15 to +25°C.
   Protect the microplate from light during this incubation period.

5. Measure the absorbance of the samples at 490 or 492 nm according to the available filters using an ELISA reader (Fig. 6).

Use a reference wavelength of >600 nm.

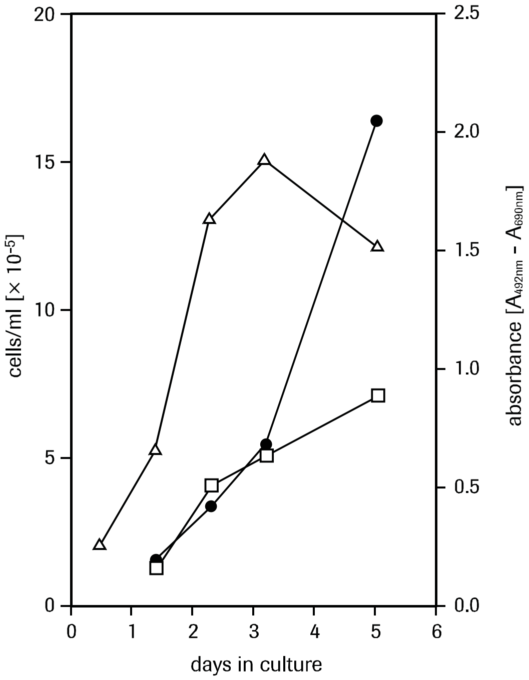

Fig. 6: Correlation of cell death and LDH release in cell culture. Ag 8 cells were seeded at a concentration of 2 × 10^5 cells/ml and incubated at +37°C, 5% CO_2. At days 1, 2, 3, and 5 of culture, aliquots were removed. The amount of viable (△) and dead (●) cells were determined by Trypan Blue exclusion. LDH activity of cell-free culture supernatant (□) was determined as described above.

2.3. Parameters

Sensitivity

Depending on the individual cell type used, 0.2 to 2 × 10^4 cells/well are sufficient for most experiments (Fig. 3).

3. Troubleshooting

Observation	Possible cause	Recommendation
Weak color reaction.	Cell concentration is too low.	Titrate cell concentration.
	Substance or assay medium inhibits LDH activity.	Use Substance control II, see section **Controls**, to test substance and/or assay medium for compounds inhibiting LDH activity.
		Avoid culture media containing pyruvate.
Strong color reaction present in Low controls.	Cell concentration is too high.	Titrate cell concentration.
	Substance or assay medium have LDH activity.	Use Substance control I, see section **Controls**, to test substance and/or assay medium for compounds with LDH activity.
	High spontaneous release due to poor condition of the cells used in the assay.	Check culture conditions; some cell lines do not survive in serum-free media even at short incubation times. Increase serum concentration to approximately 1 to 5%.
Strong color reaction with low absorbance values.	Background values too high.	High background values may lead to low absorbance values if background is automatically subtracted by the plate reader.
	Substance or assay medium have LDH activity.	Use Substance control I, see section **Controls**, to test substance and/or assay medium for compounds with LDH activity.
Strong color reaction in effector cells controls.	Poor conditions of the effector cells due to inappropriate isolation or culture conditions.	Improve cell culture conditions.
		Separate viable effector cells from dead cells by density gradient centrifugation.

4. Additional Information on this Product

4.1. Test Principle

The cell-free culture supernatant is collected and incubated with the reaction mixture from the kit. LDH activity is determined in an enzymatic test:

1. NAD^+ is reduced to NADH/H^+ by the LDH-catalyzed conversion of lactate to pyruvate.

2. The catalyst (diaphorase) transfers H/H^+ from NADH/H^+ to the tetrazolium salt INT which is reduced to formazan (Fig. 7).

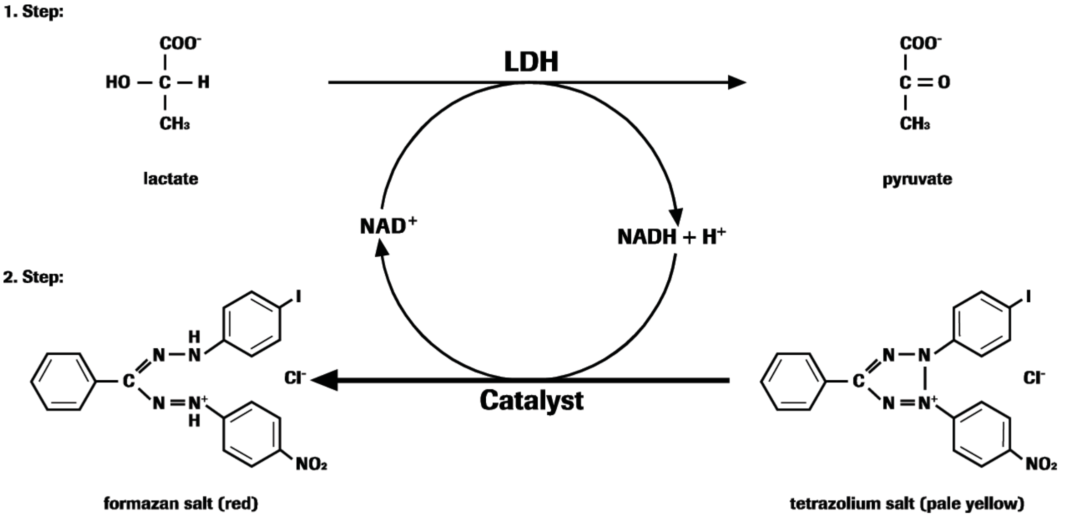

Fig. 7: In the first step, released lactate dehydrogenase (LDH) reduces NAD^+ to $NADH^+$ H^+ by oxidation of lactate to pyruvate. In the second enzymatic reaction 2 H are transferred from $NADH^+$ H^+ to the yellow tetrazolium salt INT (2-[4-iodophe-nyl]-3-[4-nitrophenyl]-5-phenyltetrazolium chloride) by a catalyst.An increase in the amount of dead or plasma membrane-damaged cells results in an increase of the LDH activity in the culture supernatant. This increase in the amount of enzyme activity in the supernatant directly correlates to the amount of formazan formed during a limited time period. Therefore, the amount of color formed in the assay is proportional to the number of lysed cells. The formazan dye formed is water soluble and has a broad absorption maximum at approximately 500 nm, whereas the tetrazolium salt INT shows no significant absorption at these wavelengths (Fig. 8).

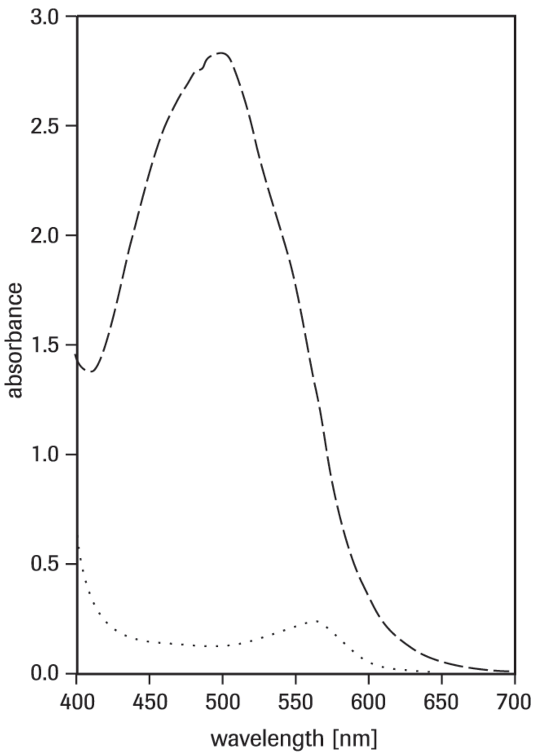

Fig. 8: Absorbance spectra of the working solution of the Cytotoxicity Detection Kit (LDH). The reaction mixture of the Cytotoxicity Detection Kit (LDH) was added to RPMI 1640 with 1% BSA and the absorbance spectra was measured in the absence (.......) and presence (– – –) of LDH.

How this product works

Cell death is classically evaluated by quantifying plasma membrane damage. The need for sensitive, quantitative, reliable, and automated methods for precisely determining cell death led to the development of several standard assays for the quantification of cellular viability.

Lactate dehydrogenase (LDH) is a stable cytoplasmic enzyme present in all cells. It is rapidly released into the cell culture supernatant when the plasma membrane is damaged. With the use of the Cytotoxicity Detection Kit (LDH), activity can easily be measured in culture supernatants by a single measurement at one time point. A spectrophotometric microplate reader (ELISA reader) may be used to simultaneously measure multiple wells and thereby makes easy processing of a large number of samples possible. The test is safe as no radioactive isotopes are used.

5. Supplementary Information

5.1. Conventions

To make information consistent and easier to read, the following text conventions and symbols are used in this document to highlight important information:

Text convention and symbols	
Information Note: Additional information about the current topic or procedure.	
**Important Note: Information critical to the success of the current procedure or use of the product.**	
①②③ etc.	Stages in a process that usually occur in the order listed.
❶❷❸ etc.	Steps in a procedure that must be performed in the order listed.
* (Asterisk)	The Asterisk denotes a product available from Roche Diagnostics.

5.2. Changes to previous version

Layout changes.
Editorial changes.
New information added related to the REACH Annex XIV.
Update to include new safety Information to ensure handling according controlled conditions.

5.3. Trademarks

All product names and trademarks are the property of their respective owners.

5.4. License Disclaimer

For patent license limitations for individual products please refer to:
List of biochemical reagent products.

5.5. Regulatory Disclaimer

For life science research only. Not for use in diagnostic procedures.

5.6. Safety Data Sheet

Please follow the instructions in the Safety Data Sheet (SDS).

5.7. Contact and Support

To ask questions, solve problems, suggest enhancements or report new applications, please visit our **Online Technical Support Site**.

To call, write, fax, or email us, visit **sigma-aldrich.com**, and select your home country. Country-specific contact information will be displayed.

1120.11653083001 ⑫

Roche Diagnostics GmbH
Sandhofer Strasse 116
68305 Mannheim
Germany

Supplementary information 5

Product Manual

OxiSelect™ Trolox Equivalent Antioxidant Capacity (TEAC) Assay Kit (ABTS)

Catalog Number

XAN-5040	200 assays

FOR RESEARCH USE ONLY
Not for use in diagnostic procedures

CELL BIOLABS, INC.
Creating Solutions for Life Science Research

Introduction

Oxidative stress is a physiological condition where there is an imbalance between concentrations of reactive oxygen species (ROS) and antioxidants. However, excessive ROS accumulation will lead to cellular injury, such as damage to DNA, proteins, carbohydrates, and lipid membranes. The cellular damage caused by ROS has been implicated in the development of many disease states, such as cancer, diabetes, cardiovascular disease, atherosclerosis, and neurodegenerative diseases. Under normal physiological conditions, cellular ROS generation is counterbalanced by the action of cellular antioxidant enzymes, macro or micro molecules, as well as other redox molecules.

Antioxidants include both hydrophilic and lipophilic molecules for metabolizing ROS. These may be localized transiently within different tissues or cells. Due to their potential harmful effects, excessive ROS must be promptly eliminated from the cells by this variety of antioxidant defense mechanisms. Antioxidants commonly neutralize radicals via a hydrogen atom transfer (HAT) or single electron transfer (SET) mechanism. Although the products of ROS-induced oxidative stress are extensively used to monitor their biological effects, it is also important to evaluate the antioxidant capacity of biological fluids, cells, and extracts.

Cell Biolabs' OxiSelect™ Trolox Equivalent Antioxidant Capacity (TEAC) Assay Kit measures the total antioxidant capacity of biomolecules from a variety of hydrophilic or lipophilic samples. The TEAC Assay is based on the conversion of oxidized $ABTS^{\cdot+}$ radical to ABTS via SET or HAT antioxidant mechanisms. Antioxidants neutralize the radical ion in a concentration dependent manner, which correlates with a proportional decrease in color intensity. The antioxidant concentration, steric accessibility, and reaction kinetics on the radical adsorption will influence the antioxidant activity values. Antioxidant activity is compared to the water soluble vitamin E analog Trolox.

Cell Biolabs' OxiSelect™ TEAC Assay Kit is a versatile, fast and reliable kit for the direct measurement of total antioxidant capacity from plasma, serum, urine, cell lysates, tissue homogenates, and food extracts. Each kit provides sufficient reagents to perform up to 200 assays, including blanks, antioxidant standards and unknown samples. Both hydrophilic and lipophilic samples are compatible with the assay and it is stable over a broad pH range. The assay does not distinguish between hydrophilic and lipophilic antioxidants, thus the combined antioxidant capacity is measured. The assay is designed for use in single plate microplate readers as well as readers with high-throughput capabilities. The assay may be performed as an end point assay or run kinetically if needed. Please read the complete kit insert prior to performing the assay.

Assay Principle

Cell Biolabs' OxiSelect™ TEAC Assay Kit measures the total antioxidant capacity within a sample. Samples are compared to known concentrations of Trolox standards within a 96-well microtiter plate format. Samples and standards are added to the microplate well and, upon the addition of the primed ABTS probe, the reaction proceeds for a few minutes. The reaction is read with a standard 96-well spectrophotometric microplate reader at 405-415 nm. Antioxidant capacity is determined by comparison with the Trolox standards.

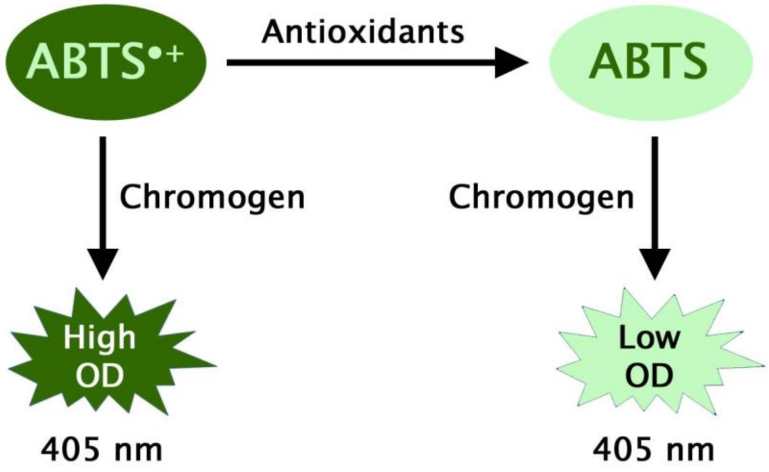

Figure 1. TEAC Assay Principle. The oxidized $ABTS^{\cdot +}$ radical is reduced to the ABTS form in the presence of antioxidants in a concentration-dependent manner, leading to a decrease in optical density at 405 nm.

Related Products

1. STA-312: OxiSelect™ Total Glutathione (GSSG/GSH) Assay Kit
2. STA-340: OxiSelect™ Superoxide Dismutase Activity Assay
3. STA-341: OxiSelect™ Catalase Activity Assay
4. STA-342: OxiSelect™ Intracellular ROS Assay Kit (Green Fluorescence)
5. STA-344: OxiSelect™ Hydrogen Peroxide/Peroxidase Assay (Fluorometric)
6. STA-345: OxiSelect™ ORAC Activity Assay
7. STA-346: OxiSelect™ HORAC Activity Assay
8. STA-347: OxiSelect™ In Vitro ROS/RNS Assay Kit (Green Fluorescence)
9. STA-349: OxiSelect™ Cellular Antioxidant Activity Assay Kit (Green Fluorescence)
10. STA-360: OxiSelect™ Total Antioxidant Capacity (TAC) Assay Kit
11. STA-844: OxiSelect™ Hydrogen Peroxide/Peroxidase Activity Assay (Colorimetric)
12. STA-859: OxiSelect™ Ferric Reducing Antioxidant Power (FRAP) Assay Kit
13. STA-860: OxiSelect™ Ascorbic Acid Assay Kit (FRASC)

Kit Components

1. ABTS Colorimetric Probe (Part No. 50401B): One 25 mg amber tube of powder
2. Trolox™ Standard (Part No. 50402C): One 200 µL amber tube of a 10 mM solution in ethanol
3. ABTS Primer (Part No. 50403B): One 200 mg amber tube of powder
4. Assay Buffer (10X) (Part No. 50404A): One 15 mL bottle

CELL BIOLABS, INC.
Creating Solutions for Life Science Research

Materials Not Supplied

1. Standard 96-well microtiter plates for use in microplate reader
2. 10 kDa MWCO centrifugal filter (for high protein content samples)
3. 1X PBS and deionized water
4. Ethanol or other organic solvent for lipid-based samples
5. Sonicator or homogenizer for sample preparations
6. 10 µL to 1000 µL adjustable single channel micropipettes with disposable tips
7. 50 µL to 300 µL adjustable multichannel micropipette with disposable tips
8. Spectrophotometric microplate reader capable of reading 405-415 nm

Storage

Upon receipt store the Trolox Standard at -20ºC. Store all remaining kit components at room temperature.

Preparation of Reagents

Reagents may be prepared for either hydrophilic or lipophilic samples. Although many lipophilic samples are soluble upon dilution with 1X PBS, the kit reagents may be prepared in ethanol to ensure complete solubility.

- 10X ABTS Primer: Dissolve 10 mg of ABTS Primer in 1.5 mL deionized water. Vortex thoroughly until dissolved. Store the 10X ABTS Primer at 4ºC and protected from light for up to 24 hours.
- 50X ABTS Reagent: Prepare the ABTS Reagent by first priming the ABTS Colorimetric Probe. Dilute the 10X ABTS Primer solution 1:10 in 1.3 mL total volume with 5 mg of ABTS powder. (e.g. Dissolve 5 mg ABTS powder in 1.170 mL deionized water and then add 130 µL 10X ABTS Primer solution). Mix to homogeneity. Allow the 50X ABTS Reagent to incubate at room temperature protected from light for at least 4 hours. The solution will turn from light green to dark green. Once complete, store the 50X ABTS Reagent at 4ºC and protected from light for up to 48 hours.
- 1X Assay Buffer: Dilute the stock Assay Buffer (10X) 1:10 with deionized water. Mix to homogeneity. Store the 1X Assay Buffer at 4ºC up to six months.

Preparation of Samples

These preparation protocols are intended as a guide for preparing unknown samples. The user may need to adjust the sample treatment accordingly. All samples should be assayed immediately or stored for up to 2 months at -80°C. A trial assay with a representative test sample should be performed to determine the sample compatibility with the dynamic range of the standard curve. High levels of interfering substances may cause variations in results. Samples may be diluted in 1X Assay Buffer as necessary before testing. Run proper controls as necessary. Always run a standard curve with samples.

Note: Please see the Potential Interference and Compatibility section for chemicals that may interfere with assay results.

- Serum*: Collect blood in a tube with no anticoagulant. Allow the blood to clot at room temperature for 30 minutes. Centrifuge at 2500 x g for 20 minutes. Remove the yellow serum supernatant without disturbing the white buffy layer. Samples should be tested immediately or frozen at -80°C.
- Plasma*: Collect blood sample and add to a blood collection tube containing heparin as the anticoagulant. Centrifuge at 3,000 rpm for 10-15 minutes at 4ºC. Remove the upper yellow plasma supernatant layer without disturbing the white buffy coat (leukocytes). Samples should be tested immediately or frozen at -80°C.

**Note: Hemolyzed plasma or serum should be avoided. Heparinized plasma is recommended over EDTA plasma.*

- Cell lysates: Lyse 1-2 x 10^6 cells/mL by sonication or multiple freeze-thaw cycles in 4 volumes of cold PBS or 1X Assay Buffer. Centrifuge at 12,000 rpm for 15 minutes at 4ºC and remove insoluble cell material. A high concentration of protein may interfere with the assay. In this case, filter the sample with a 10kDa MWCO centrifugal filter before assaying (to reduce protein interference and turbidity). Test samples immediately or store at -80°C.
- Tissue lysates/homogenates: Homogenize/sonicate approximately 10 mg of tissue in 1-2 mL of cold PBS or 1X Assay Buffer. Centrifuge the homogenate at 12,000 rpm for 15 minutes at 4ºC and collect the supernatant. A high concentration of protein may interfere with the assay. In this case, filter the sample with a 10kDa MWCO centrifugal filter before assaying (to reduce protein interference and turbidity). Test samples immediately or store at -80°C.
- Urine: Test neat or diluted with PBS or 1X Assay Buffer if appropriate.
- Lipophilic Fractions: Dissolve lipophilic samples in 100% ethanol or acetone and then dilute in 100% ethanol or 50% acetone. Incubate the mixture for 1 hour at room temperature with mixing. Further dilute samples as necessary prior to testing.
- Food Samples: Results may vary depending on sample source and purification. Dilution and preparation of these samples is at the discretion of the user, but use the following guidelines:
    - Solid or High Protein Samples: Weigh solid sample and then homogenize after adding deionized water (1:2, w/v). Centrifuge the homogenate at 10,000 x g for 10 minutes at 4ºC. Recover the supernatant which is the water-soluble fraction. The insoluble fraction (pulp) is further extracted by adding acetone (1:4, w(solid pulp)/v) and mixing at room temperature for 30-60 minutes. Centrifuge the extract/solid at 10,000 x g for 10 minutes at 4ºC. Recover the acetone extract and dilute with PBS, 1X Assay Buffer, ethanol or water as necessary prior to running the assay. The TEAC value is calculated by combining the results from the water-soluble fraction and the acetone extract from the pulp fraction.
    - Aqueous Samples: Centrifuge the sample at 10,000 x g for 10 minutes at 4ºC to remove any particulates. Dilute the supernatant in PBS or 1X Assay Buffer as necessary prior to running the assay.

Potential Interference and Compatibility

Table 1 contains some common substance compatibilities. The listed concentration values are prior to performing the assay. Dilution of the substance/buffer, and ultimately samples, may be required to

completely eliminate interference. Even with some interference, accurate quantitation can be achieved by running standards in the same buffer as samples, although kit sensitivity may be compromised.

Substance	Compatible Concentration
Acetone	50%
Deoxycholic acid	0.5%
Dithiothreitol	Not Compatible
DMSO	5%
EDTA	Not Compatible
Glycerol	50%
100 mM Hepes	Not Compatible
Methanol	50%
2-Mercaptoethanol	Not Compatible
NP-40	Not Compatible
PBS	undiluted
SDS	Not Compatible
Tris, pH 7.5	500 mM
Triton-X 100	0.1%
Tween-20	Not Compatible

Table 1. Substance Compatibilities

Preparation of Trolox Standard Curve

I. Hydrophilic (aqueous) Samples

1. Prepare fresh aqueous standards by diluting the 10 mM Trolox Standard stock solution to 0.3 mM in Assay Buffer (e.g. add 15 µL of Trolox Standard stock tube to 485 µL of Assay Diluent). Prepare a series of the remaining Trolox standards according to Table 1 below.

Tubes	10 mM Trolox™ Standard (µL)	Assay Diluent (µL)	Resulting Trolox™ Concentration (µM)
1	15	485	300
2	250 of tube #1	250	150
3	250 of tube #2	250	75
4	250 of tube #3	250	37.5
5	250 of tube #4	250	18.8
6	250 of tube #5	250	9.4
7	250 of tube #6	250	4.7
8	0	250	0

Table 2. Preparation of Standards for use when testing Hydrophilic Samples.

Note: Do not store diluted Trolox Standard solutions.

II. Lipophilic Samples

1. Prepare fresh lipophilic standards by diluting the 10 mM Trolox Standard stock solution to 0.3 mM in ethanol (e.g. add 15 µL of Trolox Standard stock tube to 485 µL of ethanol).
2. Prepare a series of the remaining Trolox standards according to Table 2 below.

Tubes	10 mM Trolox™ Standard (µL)	100% Ethanol (µL)	Resulting Trolox™ Concentration (µM)
1	15	485	300
2	250 of tube #1	250	150
3	250 of tube #2	250	75
4	250 of tube #3	250	37.5
5	250 of tube #4	250	18.8
6	250 of tube #5	250	9.4
7	250 of tube #6	250	4.7
8	0	250	0

Table 3. Preparation of Standards for use when testing Lipophilic Samples.

Note: Do not store diluted Trolox Standard solutions.

Assay Protocol

Each Trolox Standard and sample should be assayed in duplicate or triplicate. A freshly prepared standard curve should be used each time the assay is performed.

1. Add 25 µL of the diluted Trolox Standards or samples to a 96-well microtiter plate.
2. Dilute the 50X ABTS Reagent 1:50 in either 1X Assay Buffer (Hydrophilic samples) or 75% ethanol (Lipophilic samples) (e.g. for 100 assays, combine 300 µL ABTS Reagent with 14.7 mL of either Assay Buffer or 75% ethanol). Vortex thoroughly.
3. Add 150 µL of the diluted ABTS Reagent to each well using either a multichannel pipette or a plate reader liquid handling system. Mix thoroughly. Immediately begin timing the reaction.
4. Incubate 5 minutes on an orbital shaker.
5. Read the plate at 405-415 nm immediately. The green colored product will fade over time.

Note: Trolox standards will completely react within 5 minutes. Different antioxidants may need more or less time to completely react. In these situations, the assay may also be performed kinetically by reading the plate at multiple time points.

Example of Results

The following figures demonstrate typical OxiSelect™ TEAC Assay results (hydrophilic). One should use the data below for reference only. This data should not be used to interpret or calculate actual sample results.

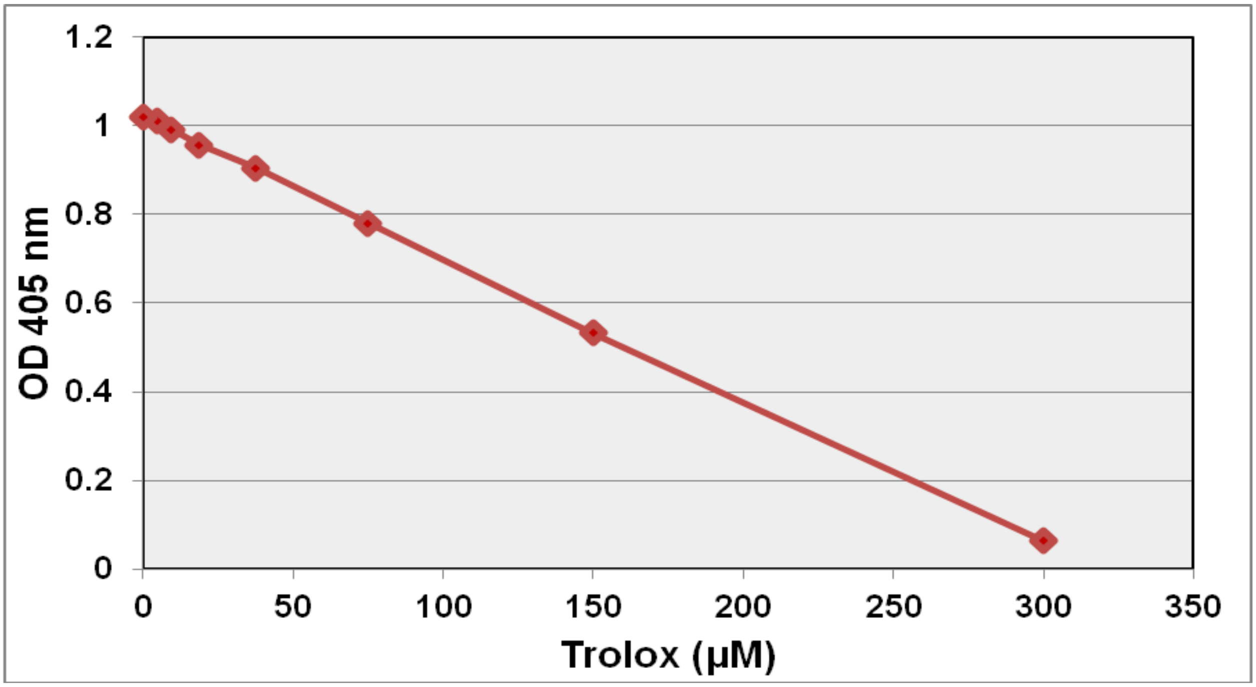

Figure 2: TEAC Assay Standard Curve (Hydrophilic).

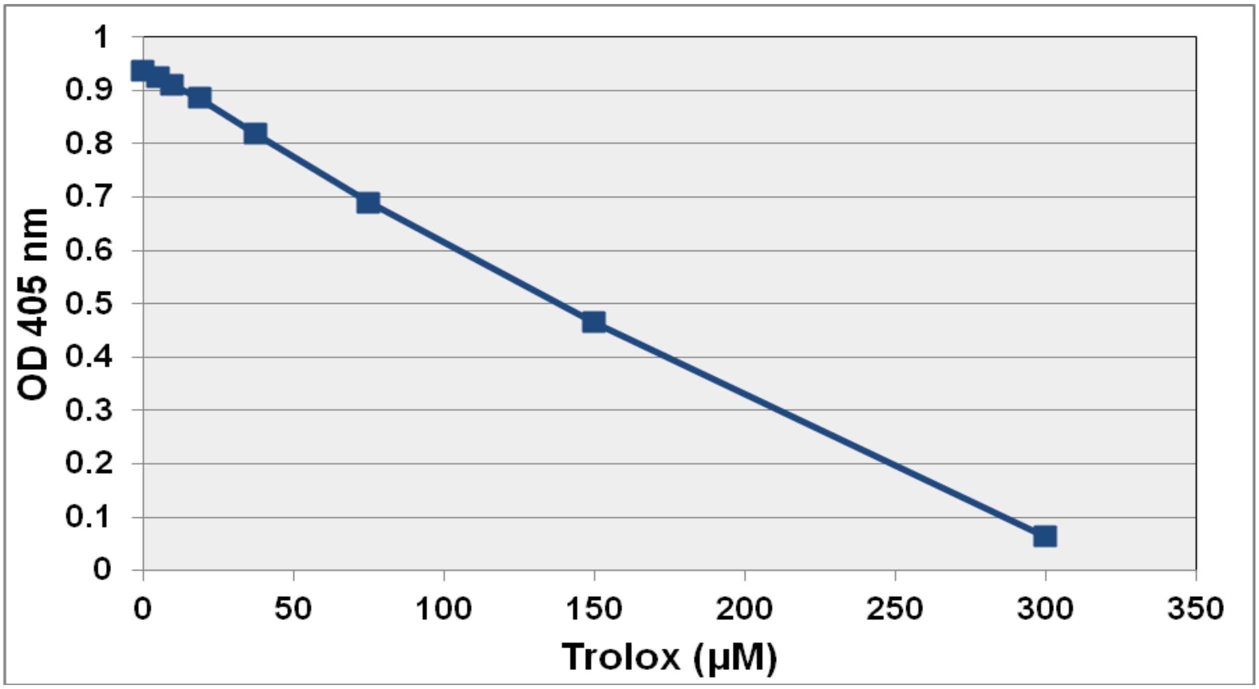

Figure 3: TEAC Assay Standard Curve (Lipophilic).

CELL BIOLABS, INC.
Creating Solutions for Life Science Research

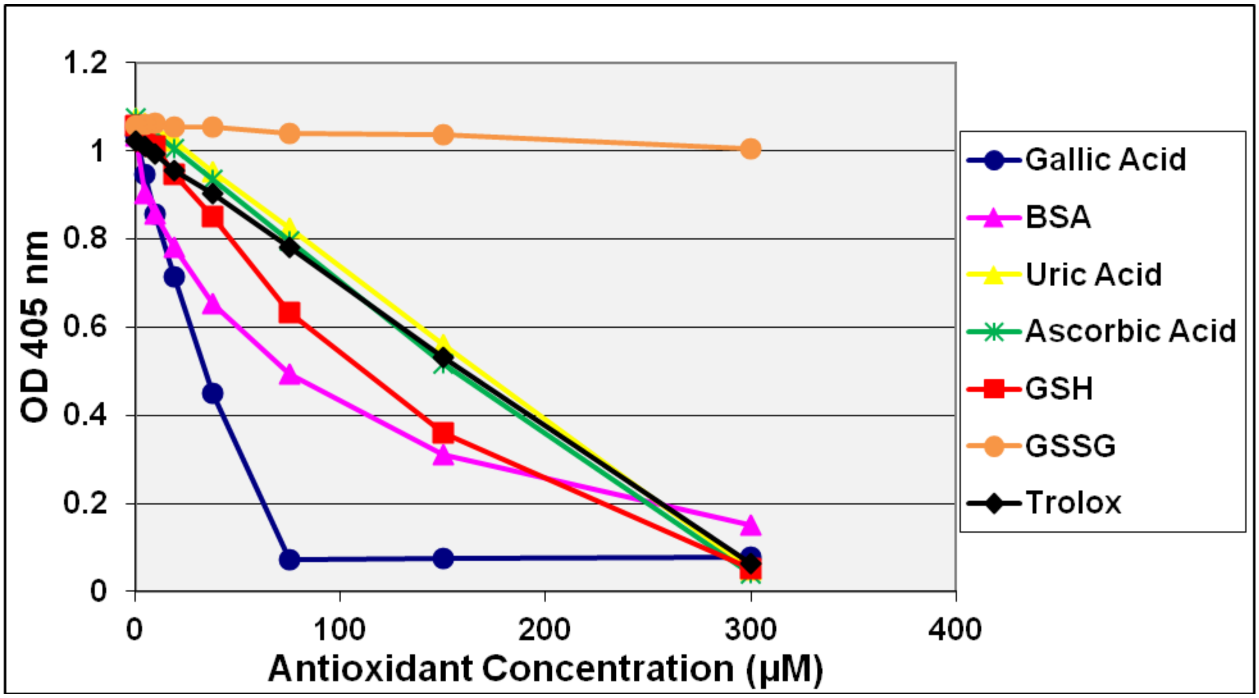

Figure 4: TEAC Assay of various hydrophilic antioxidants.

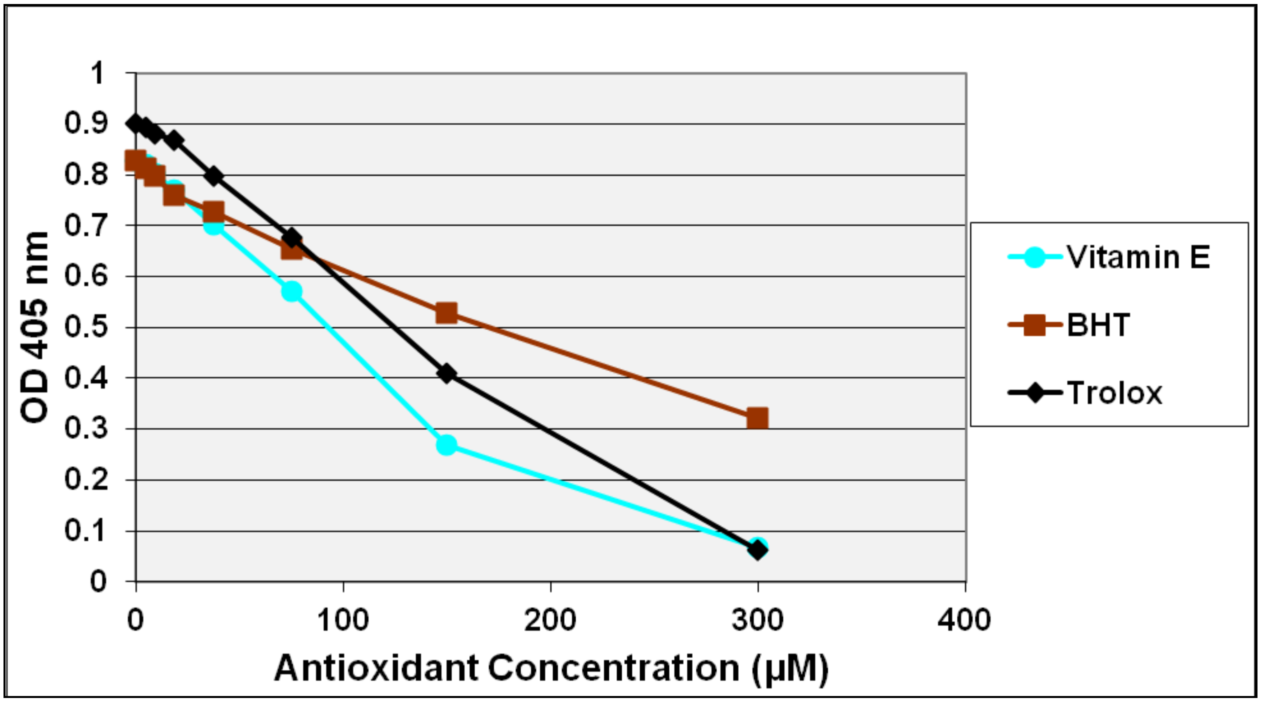

Figure 5: TEAC Assay results of various lipophilic antioxidants.

CELL BIOLABS, INC.
Creating Solutions for Life Science Research

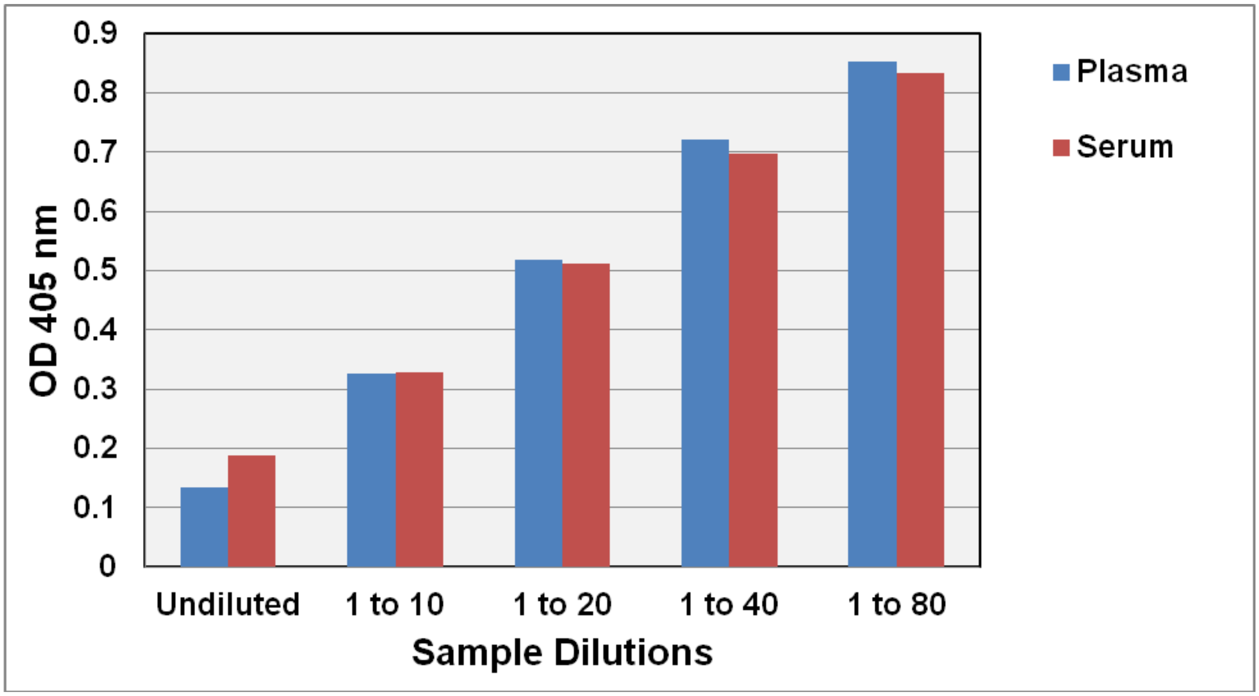

Figure 6: TEAC Assay results of human serum and plasma samples.

Calculation of Results

1. Calculate the average absorbance values for every standard, control, and sample.
2. Plot the average absorbance for the standards against the final concentration of the trolox standards from Table 2 (hydrophilic assays), or Table 3 (lipophilic assays) to determine the best curve. See Figures 2 and 3 for example standard curves.
3. Determine the antioxidant concentration, as µM Trolox equivalents (TEAC value), in of the samples with the equation obtained from the linear regression analysis of the standard curve. Substitute the average absorbance values for each sample. Remember to account for dilution factors. Only use values within the range of the standard curve.
4.

$$\textbf{Antioxidant } (\mu\textbf{M}) = \left[\frac{\textbf{(Sample average absorbance – (y-intercept))}}{\textbf{Slope}}\right] \times \textbf{Sample dilution}$$

Results may also be expressed as micromoles of Trolox equivalents per gram of sample (TE µmol / g). The formula weight of Trolox is 250.29 g / mol.

References

1. Halliwell, B. (1996) *Free Radic. Res.* **25**: 57-74.
2. Huang, H., et al. (2005) *J. Agric. Food Chem.* **53**: 1841-1856.
3. Kambayashi, Y., et al. (2009) *J. Clin. Biochem. Nutr.* **44**: 46-51.
4. Koracevic, D., et al. (2001) *J. Clin. Pathol.* **54**: 356-361.

5. Miller, N.J., et al. (1997) *Free Radic. Res.* **26**: 195-199.
6. Rice-Evans, C.A. (2000) *Free Radic. Res.* **33**: S59-66.

Trolox™ is a trademark of Hoffman-LaRoche

Recent Product Citations

1. Elhassaneen, Y. A. et al. (2023). Influence of Novel Freezing and Storage Technology on Nutrient Contents, Bioactive Compounds and Antioxidant Capacity of Black Eggplant. *Journal of Agriculture and Crops*. **93**:338–352. doi: 10.32861/jac.93.338.352.
2. Pérez-Peiró, M. et al. (2023). Nitrosative and Oxidative Stress, Reduced Antioxidant Capacity, and Fiber Type Switch in Iron-Deficient COPD Patients: Analysis of Muscle and Systemic Compartments. *Nutrients*. **15**(6):1454. doi: 10.3390/nu15061454.
3. Qin, L. et al. (2021). Systemic Profiles of microRNAs, Redox Balance, and Inflammation in Lung Cancer Patients: Influence of COPD. *Biomedicines*. **9**(10):1347. doi: 10.3390/biomedicines9101347.
4. Nallan Chakravartula, S.S. et al. (2021). Stinging Nettles as Potential Food Additive: Effect of Drying Processes on Quality Characteristics of Leaf Powders. *Foods*. **10**(6):1152. doi: 10.3390/foods10061152.

Warranty

These products are warranted to perform as described in their labeling and in Cell Biolabs literature when used in accordance with their instructions. THERE ARE NO WARRANTIES THAT EXTEND BEYOND THIS EXPRESSED WARRANTY AND CELL BIOLABS DISCLAIMS ANY IMPLIED WARRANTY OF MERCHANTABILITY OR WARRANTY OF FITNESS FOR PARTICULAR PURPOSE. CELL BIOLABS's sole obligation and purchaser's exclusive remedy for breach of this warranty shall be, at the option of CELL BIOLABS, to repair or replace the products. In no event shall CELL BIOLABS be liable for any proximate, incidental or consequential damages in connection with the products.

Contact Information

Cell Biolabs, Inc.
7758 Arjons Drive
San Diego, CA 92126
Worldwide: +1 858 271-6500
USA Toll-Free: 1-888-CBL-0505
E-mail: tech@cellbiolabs.com
www.cellbiolabs.com

©2016-2023: Cell Biolabs, Inc. - All rights reserved. No part of these works may be reproduced in any form without permissions in writing.

Fast FRAP Antioxidant capacity assay kit

KF01006

100/200/400 test (96 well plate)

Materials:

100 test

Product	Quantity	Storage
Reagent A	1 bottle	RT
Standard (Ferrous Ammonium Sulphate)	1 vial (Powder)	RT

200 test

Product	Quantity	Storage
Reagent A	2 bottles	RT
Standard	2 vials (Powder)	RT

400 test

Product	Quantity	Storage
Reagent A	4 bottles	RT
Standard	4 vials (Powder)	RT

Volume of sample required:

10 µL/test

Types of sample compatible:

Biological fluids, foods and beverages, plant extracts

Linear range:

100 – 800 µM FRAP

Type of detection:

Colorimetric (593 nm)

Sensitivity:

0.001 Absorbance $_{593nm}$/FRAP (µM)

Time required for the assay:

10 min

Sample preparation:

Smoothies: Use a food processor to make the smoothie. Filter through a 0.2 µm membrane filter. Cool on ice to assay or freeze at -20°C.

Beverages, fruits and vegetables: If solubilized, they can be used directly with appropriate dilutions.

Tissue homogenate: **1.** Rinse tissue with PBS (pH 7.4). **2.** Homogenize in 5-10 mL of cold buffer/g tissue. **3.** Centrifuge at 10,000 x g for 15 min at 4°C. **4.** Collect supernatant to assay or freeze.

Cell lysate: **1.** Centrifuge sample at 1,000-2,000 x g for 10 min at 4°C.Do not use proteolitic enzymes. **2.** Homogenize/ sonicate cell pellet with 1-2 mL of cold buffer. **3.** Centrifuge at 10,000 x g for 15 min at 4°C. **4.** Collect supernatant to assay or freeze.

Plasma: **1.** Centrifuge blood sample (with anticoagulant) at 700-1,000 x g for 10 min at 4°C. **2.** Collect the supernatant to assay or freeze.

Reagent Preparation:

Standard preparation (Iron (II) Standard): Add exactly 1 mL of ddH_2O to the standard vial that is going to be used immediately and mix well. Dilute standard 1:10 with ddH_2O. For example: 100 µL standard + 900 µL Reagent ddH_2O.

Fast FRAP Antioxidant capacity assay kit

KF01006

100/200/400 test (96 well plate)

Standard curve preparation:

Table 1. Reagent volumes needed to carry out the standard curve.

Sample	ddH_2O [µL]	Standard [µL]	*FRAP [µM]
**S1 (Blank)**	100	0	0
**S2**	97.5	2.5	100
**S3**	95	5	200
**S4**	90	10	400
**S5**	85	15	600
**S6**	80	20	800

Figure 1. Typical standard curve for FRAP assay.

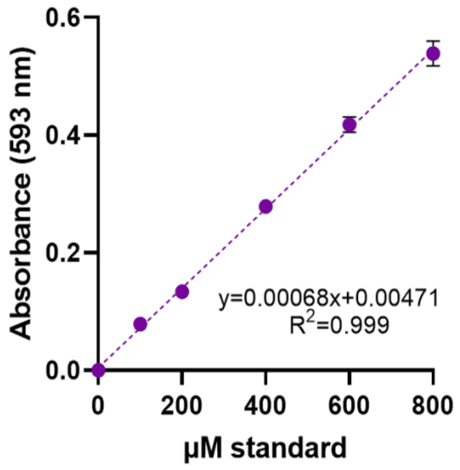

*Antioxidant activity is expressed as FRAP values (Ferric Reducing Ability of Plasma). These values are related to Fe^{2+} concentration.

Performing the assay:

1. Add 10 µL of the sample or standard in each well.
2. Add 220 µL of Reagent A in each well (for sample blanks add 220 µL of ddH_2O).
3. Mix the mixture for 4 minutes under continuous stirring.
4. Read the absorbance at 593 nm.

Data analysis:

1. If the spectrophotometer or microplate reader was not zeroed with the blank, then average the blank values and subtract it from the standards.
2. Create an antioxidant standard curve by plotting Absorbance 593 nm (y-axis) *vs.* µM standard (x-axis) (See Figure 1. for a typical standard curve)
3. Subtract the sample blanks from the values obtained for each sample. Determine the unknown sample concentration using the standard curve with the following formula:

$$\text{FRAP } (\mu\text{M}) = \left(\frac{\text{Absorbance } (593\text{ nm}) - \text{intercept}}{\text{slope}}\right) \text{x dilution factor}$$

ORAC Antioxidant capacity assay kit

KF01004

100/200/400 tests (96 well plate)

Materials:

100 tests:

Product	Quantity	Storage
ORAC Reagent A	1 bottle	4 ºC
ORAC Reagent B	1 vial	-20 ºC
ORAC Reagent C	1 vial (Powder)	-20 ºC
ORAC Reagent D	1 bottle	4 ºC
Standard (Trolox)	1 vial (Powder)	-20 ºC

200 tests:

Product	Quantity	Storage
ORAC Reagent A	2 bottles	-20 ºC
ORAC Reagent B	2 vials	4 ºC
ORAC Reagent C	2 vials (Powder)	-20 ºC
ORAC Reagent D	1 bottle	4 ºC
Standard (Trolox)	2 vials (Powder)	-20 ºC

400 tests:

Product	Quantity	Storage
ORAC Reagent A	4 bottles	4 ºC
ORAC Reagent B	4 vials (Powder)	-20 ºC
ORAC Reagent C	4 vials (Powder)	-20 ºC
ORAC Reagent D	1 bottle	4 ºC
Standard (Trolox)	2 vials (Powder)	-20 ºC

Volume of sample required:
15 µL/test

Types of sample compatible:
Biological fluids, cell lysates, tissue homogenates, foods and beverages

Linear range:
10 – 100 µM TEAC

Type of detection:
Fluorimetric (Ex: 485 nm/Em: 528-538 nm)

Sensitivity:
0.20 *AUC/µM TEAC

Time required for the assay:
100 min

*AUC: Area Under Curve

Sample Preparation:

Tissue homogenate: **1.** Rinse tissue with PBS (pH 7.4). **2.** Homogenize in 5-10 ml of cold buffer (containing protease inhibitors)/g tissue **3.** Centrifuge at 10,000 x g for 15 min at 4ºC. **4.** Collect supernatant to assay or freeze.

Supplementary information 6

Cell lysate: **1.** Obtain a cell pellet (1 x10^6 cells). If cells are adherent, please use scraper technique to obtain it. **2.** Mix cells with 1 mL of cold PBS. To lysis use homogenization or sonication on ice. **3.** Centrifuge between 1 and 5,000 x g for 10 min at 4 ºC. **4.** Collect supernatant to assay or freeze.

Plasma: **1.** Centrifuge blood sample (with anticoagulant) at 700-1,000 x g for 10 min at 4ºC. **2.** Collect the supernatant to assay or freeze.

Food: **1.** Homogenize solid food in a small volume of cold PBS. Liquid foods do not need preparation. **2.** Store at -20°C until it is used.

Reagent Preparation:

Solution B: In a separate tube, mix 5 µl of Reagent B with 20 ml of Reagent A. This solution is enough for 100 tests and remains stable in the fridge for a few hours.

Solution C: Add 4.5 ml of Reagent A to the vial of Reagent C and mix. Take 3.75 mL of this solution and add 2.25 mL of Reagent A. This reagent is stable only for a few hours, discarding the remaining solution after use.

ORAC standard (Trolox): Add exactly 500 µl of Reagent D to the standard vial. Mix well and add 500 µl of Reagent A. Then, dilute this solution 1:100 with Reagent A. For example: 10 µl standard + 990 µl Reagent A.

Standard curve preparation:

Table 1. Reagent volumes needed to carry out the standard curve

Sample	Reagent A [µl]	Standard [µl]	*TEAC [µM]
**S1 (Blank)**	100	0	0
**S2**	90	10	10
**S3**	75	25	25
**S4**	50	50	50
**S5**	25	75	75
**S6**	0	100	100

*Antioxidant capacity is expressed as TEAC (Trolox Equivalent Antioxidant Capacity)

Figure 1. Typical standard curve for ORAC assay

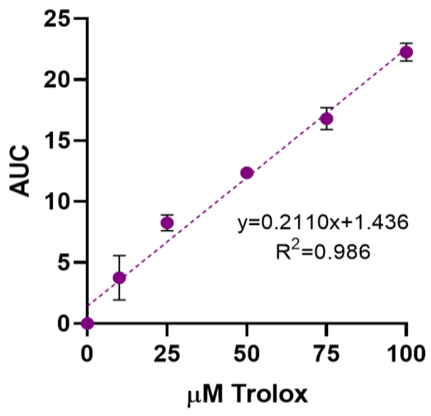

Performing the assay:

1. Prepare all reagents and 96 well plate.
2. Equilibrate the plate reader incubation chamber to 37°C before beginning. Set-up plate reader to perform a kinetic read for 60 minutes with 1 minute intervals (total reads 61). Excitation = 485 nm; Emission = 528 - 538 nm.
3. Add 15 µl of the sample or standard in each well.
4. Add 90 µl of Solution B previously prepared (see Reagent preparation) in each well. Mix.
5. Incubate at 37 ºC for 15 minutes.
6. Add 45 µl of Solution C previously prepared (see Reagent preparation) in each well. Mix.
7. Read the fluorescence for 60 minutes.

Data analysis:

1. Calculate normalized values for each well at each point time as the fluorescence value divided by the first measurement: $RF_n = \frac{F_n}{F_1}$ where F_n is the value at each point time; F_1 is the value at first measurement
2. Calculate the area under the curve for each well with the following formula:

$$\mathrm{AUC} = 0.5 + \sum_{1}^{60} \mathrm{RF} + \frac{\mathrm{RF}_{61}}{2}$$

3. Subtract the average blank value S1 from the rest of the values of the standard. Then, create a standard curve by plotting the AUC (y-axis) vs. standard, µM Trolox (x-axis) (Example in figure 1).
4. Subtract the average blank value S1 from the rest of the values obtained for each sample. Determine the unknown sample concentration using the standard curve:

$$\mathrm{TEAC}\ (\mu\mathrm{M}) = \left(\frac{\mathrm{AUCsample} - \mathrm{intercept}}{\mathrm{slope}}\right) \mathrm{x\ dilution\ factor}$$

HORAC Antioxidant Assay Kit
(60 point kit)
Cat# AOX-6

INSTRUCTION MANUAL ZBM0118.00

STORAGE CONDITIONS

All orders are delivered via Federal Express Priority courier at 4°C.
All orders must be processed immediately upon arrival. Any adverse conditions upon arrival must be reported within 7 days.

Fluorescein Solution and Radical Initiator solution
Remove from box and store at 4°C
Gallic Acid Standard and Fenton Reagent
Remove from box and store at -20°C
AOX-6 Assay Buffer and black assay plate
Store at Room Temperature
Long-term Storage
Reagents are good for at least 3 months upon arrival if stored properly.

For *in vitro* Use Only

LIMITED PRODUCT WARRANTY

This warranty limits our liability to replacement of this product. No other warranties of any kind, expressed or implied, including without limitation, implied warranties of merchantability or fitness for a particular purpose, are provided by Zen-Bio, Inc. Zen-Bio, Inc. shall have no liability for any direct, indirect, consequential, or incidental damages arising out of the use, the results of use, or the inability to use this product.

ORDERING INFORMATION AND TECHNICAL SERVICES

- **Zen-Bio, Inc.**
- **3920 South Alston Ave**
- **PO Box 13888**
- **Durham, NC 27713**
- **Telephone** (919) 547-0692
- **Facsimile (FAX)** (919) 547-0693
- **Toll Free** **1-866-ADIPOSE (866)-234-7673**
- **Electronic mail (e-mail)** **information@zen-bio.com**
- **World Wide Web** http://www.zenbio.com

TABLE OF CONTENTS

	PAGE#
Introduction	3
Principle of Assay	4
Items Included in the Kit	4
Sample Preparation	5
Assay Procedure	6
Gallic Acid Standard Curve	7
Appendix A: Plate layout	8
Appendix B: Protocol Flowchart	9
References	9

INTRODUCTION

Free radicals and reactive oxygen species (ROS) are highly reactive molecules that are generated by normal cellular processes, environmental stresses, and UV irradiation. ROS react with cellular components, damaging DNA, carbohydrates, proteins, and lipids causing cellular and tissue injury. Excess production of reactive oxygen species can also lead to inflammation, premature aging disorders, and several disease states, including cancer, diabetes, and atherosclerosis. Organisms have developed complex antioxidant systems to protect themselves from oxidative stress, however, excess ROS can overwhelm the systems and cause severe damage.

The Zen-Bio HORAC (Hydroxyl (HO•) Radical Absorbance Capacity) Antioxidant Assay Kit can be used to determine the total antioxidant capacity of biological fluids, cells, and tissue. It can also be used to assay the antioxidant activity of naturally occurring or synthetic compounds for use as dietary supplements, topical protection, and therapeutics. The assay measures the loss of fluorescein fluorescence over time due to hydroxyl-radical formation by the mixture of hydrogen peroxide and oxidizable metal ions (Co(II)). Gallic Acid [3,4,5-Trihydroxybenzoic acid], a simple phenolic compound, serves as a positive control inhibiting fluorescein decay in a dose dependent manner. The HORAC assay is a kinetic assay measuring fluorescein decay and antioxidant protection over time. The antioxidant activity in biological fluids, cells, tissues, and natural extracts can be normalized to equivalent Gallic Acid units to quantify the composite antioxidant activity present. This assay measures antioxidant activity by hydrogen atom transfer and when combined with Zen-Bio's other antioxidant assay kits, provides a comprehensive analysis of a test sample's antioxidant activity.

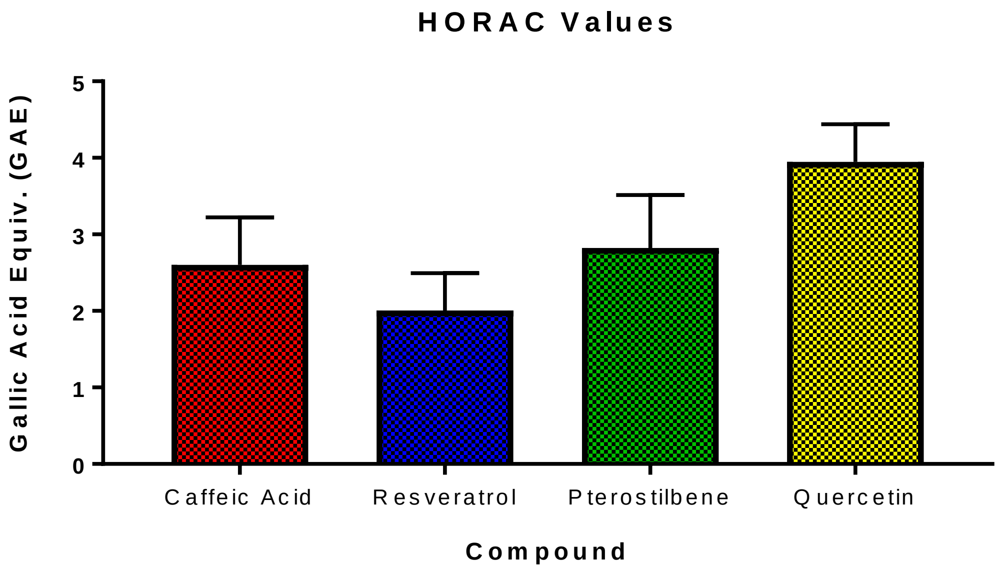

Figure 1. Effects of antioxidants in HORAC assay
Caffeic Acid, Resveratrol, Pterostilbene and Quercetin were tested for their antioxidant activity in the HORAC antioxidant assay.

PRINCIPLE OF THE ASSAY

Hydroxyl radicals ($HO^{\bullet}$) are formed from the mixture of oxidizable cobalt(II) ions and hydrogen peroxide in solution. The hydroxyl radical can oxidize fluorescein (3',6'-dihydroxy-spiro[isobenzofuran-1[3H], 9'[9H]-xanthen]-3-one) to generate a product without fluorescence. Antioxidants suppress this reaction by a hydrogen atom transfer mechanism, inhibiting the oxidative degradation of the fluorescein signal. The fluorescence signal is measured over 45 minutes by excitation at 485 nm, emission at 535 nm. The concentration of antioxidant in the test sample is proportional to the fluorescence intensity through the course of the assay and is assessed by comparing the net area under the curve to that of a known antioxidant, gallic acid.

H2O2 * Co(II)   HO$^{\bullet}$ OH^{-} Co(III)

fluorescein ⟶ **non-fluorescent product**

[Antioxidants inhibit the oxidation of fluorescein by hydrogen atom transfer]

ITEMS INCLUDED IN THE KIT

ITEM	DESCRIPTION	Cap Color	UNIT	QTY	STORAGE
Blank Assay Plates	96-**well assay plates, black clear bottom**	---	PLATE	1	-----
AOX Assay Buffer	35 ml	---	BOTTLE	1	RT
Radical Initiator	53.3x stock solution	---	100 µl /VIAL	1	4°C
Gallic Acid Solution	5mM in AOX-6 Buffer	---	200 µl /VIAL	1	-20°C
Fenton Reagent	2 ml metal ion solution	---	BOTTLE		-20°C
Fluorescein Solution	47.3x stock		300 µl /VIAL	1	4°C
Tray	For multi-channel pipettes, clear polyvinyl	---	EACH	1	-----

Other equipment/reagents required but not provided with the kit:

- Multi-channel pipet, single channel pipet and pipet tips
- Tubes for preparing standards and working solutions
- Fluorescence plate reader able to perform excitation=485nm; emission=528 -538nm (cutoff=530nm, if necessary)
- Fluorescence plate reader with incubator chamber set to 37°C

SAMPLE PREPARATION

Cell Lysate Preparation

1. Scrape ~1 x10^6 cells and centrifuge at 1,000xg to prepare a cell pellet. DO NOT use proteolytic enzymes such as trypsin but scrape using a rubber policeman or cell scraper tool.
2. Homogenize or sonicate the cell pellet on ice in 1ml cold AOX Assay buffer
3. Centrifuge at 10,000 x g for 15 minutes at 4°C.
4. Remove the supernatant and keep on ice until ready to use in the assay.
5. If not using the same day, store the samples at -80°C.
6. Data is expressed as Gallic Acid equivalents (GAE) per cell number (i.e. µmole GA/10^6 cells)

Tissue Lysate Preparation

1. Homogenize tissue samples on ice in cold buffer at ~200mg tissue per ml cold buffer
2. Centrifuge at 10,000 x g for 15 minutes at 4°C.
3. Remove the supernatant and keep on ice until ready to use in the assay.
4. If not using the same day, store the samples in small aliquots at -80°C.
5. Data is expressed as Gallic Acid equivalents (GAE) per gram of starting sample (i.e. µmole GA/g)

Plasma Preparation

1. Collect the blood in a tube containing heparin or other anticoagulant.
2. Centrifuge at 1,000 x g for 10 minutes at 4°C.
3. Remove the supernatant and keep on ice until ready to use in the assay.
4. If not using the same day, store the samples in small aliquots at -80°C.
5. Data is expressed as micromoles Gallic Acid equivalents (GAE) per volume sample (i.e. µmole GA/L) **[Dilute 100-fold in assay buffer prior to assaying].**

Serum Preparation

1. Collect the blood in a tube WITHOUT any anticoagulant. Allow the blood to clot.
2. Centrifuge at 2,000 x g for 10 minutes at 4°C.
3. Remove the supernatant and keep on ice until ready to use in the assay.
4. If not using the same day, store the samples in small aliquots at -80°C.
5. Data is expressed as micromoles Gallic Acid equivalents (GAE) per volume sample (i.e. µmole GA/L) **[Dilute 100-fold in assay buffer prior to assaying].**

Saliva Collection

1. Collect whole saliva for a defined period of time (i.e. 1-5 minutes) into polypropylene tubes.
2. Immediately place on ice or store at -80°C for later analysis.
3. Data is expressed as micromole Gallic Acid equivalents (GAE) per volume sample (i.e. µmole GA/L)

Food Extract Preparation

1. Weigh the starting material.
2. Homogenize in a small volume ice cold buffer or water.
3. Store small aliquots at -80°C for analysis.
4. When ready to assay, keep thawed samples on ice.
5. Data is expressed as Gallic Acid equivalents (GAE) per gram of starting sample (i.e. µmole GA/g)

ASSAY PROCEDURE

THIS KIT PROVIDES SUFFICIENT REAGENTS TO ASSAY 60 WELLS. AT LEAST 6 OF THESE WELLS ARE REQUIRED FOR GALLIC ACID STANDARDS

1. Equilibrate the plate reader incubation chamber to 37°C before beginning. Set-up plate reader to perform a kinetic read for 45 minutes with 1 minute intervals. Excitation = 485 nm; Emission = 528 - 538 nm (Cutoff = 530 nm, if required). **SET PLATE READER TO BOTTOM READ.**

2. Prepare fluorescein working solution from the stock solution provided by transferring **11.75ml** of AOX Assay Buffer to an empty tube (not provided) and adding **0.254ml** stock fluorescein solution. Mix and protect from light.

3. Prepare Gallic acid standards as follows:

   Briefly spin down the contents of the 5 mM Gallic acid standard tube after thawing. Pipette **160 µl** of the 5 mM Gallic acid standard solution into a tube containing **840 µl** AOX Assay Buffer and mix well by vortexing. This produces a diluted stock Gallic Acid standard of **800 µM**. Pipette **100 µl** of AOX Assay Buffer into 5 tubes (not provided). Using the table and diagram below, prepare Gallic Acid standards 600, 400, 300, 200 and 100 µM. Mix each new dilution thoroughly before proceeding to the next. The **800 µM** standard dilution serves as the highest standard, and assay buffer serves as the zero standard (or blank).

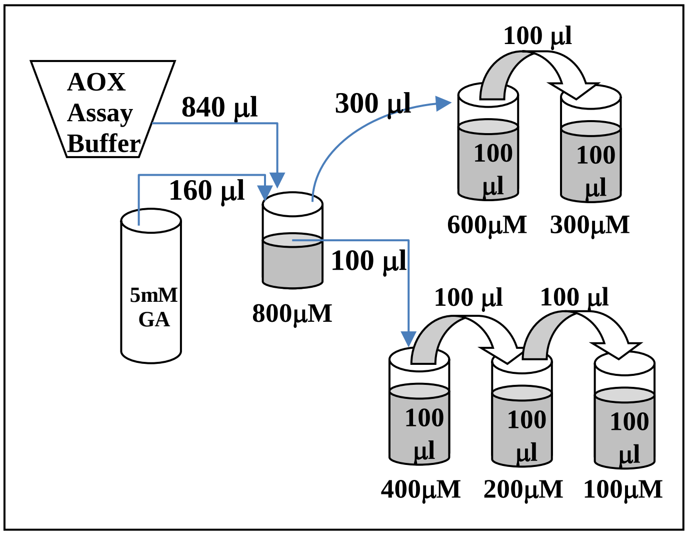

Final Conc. (µM)	Buffer (µL)	Volume (µL)	GA Solution
800	840	160	5mM Stock
600	100	300	800 µM Std
400	100	100	800 µM Std
300	100	100	600 µM Std
200	100	100	400 µM Std
100	100	100	200 µM Std

4. Add 140 µl of the working fluorescein solution to each of the **INNER 60 WELLS** of the assay plate provided. Fill the outer wells with 200 µl water to maintain a constant temperature for all wells.

5. Add 20 µl of samples or Gallic acid standards (with zero standard) to individual wells of the assay plate provided. Place plate at 37°C for at least **10** minutes. [**IF THE AOX ACTIVITY OF THE TEST SAMPLES IS UNKNOWN, WE RECOMMEND PREPARING SEVERAL DILUTIONS IN AOX ASSAY BUFFER.]**

6. While the assay plate is equilibrating to 37°C, prepare the Radical Initiator Working Solution by adding 37.5 µl of the concentrated Radical Initiator solution to 1.9625 mL of water.

7. After the assay plate has equilibrated to 37°C, add 20 μl of the Radical Initiator Working Solution to each inner 60 assay well.
8. To begin the assay, add 20 μl of the Fenton Reagent to each of the wells containing standards and samples from step 5. Place the assay plate in the 37°C plate reader and begin kinetic fluorescence reading.

GALLIC ACID STANDARD CURVE ______________________

Generate standard curve: see example below (Collected using a SpectraMax iD3)

[DO NOT use this standard curve to generate your data. This is an example.]

Kinetic RLU Values

			Gallic Acid				
Time (min)	800	600	400	300	200	100	0
0	3204677	3224222	3275543	3165677	3174140	3196064	2706418
1	3198845	3221342	3194853	3174483	3201116	3143567	2509882
2	3158966	3193611	3178068	3130211	3120474	3118326	2351955
3	3114410	3139431	3139222	3116647	3105044	3043751	2144727
4	3114958	3120308	3111903	3094643	3076547	3029441	1993790
5	3079319	3107630	3070934	3092071	3034362	2907369	1865437
6	3092500	3095182	3069419	3019674	3053490	2844406	1684772
7	3086678	3086251	3043584	3039228	2969976	2750370	1541569
8	3060684	3040795	3024565	3005937	2870750	2630228	1400518
9	3019969	3008712	2988459	2929144	2800988	2547167	1312548
10	2970267	3019721	2921819	2924410	2746422	2457060	1190740
11	3018399	3024731	2847319	2803394	2636318	2333812	1080637
12	2964877	2979408	2785634	2732468	2503402	2240730	1001914
13	2965813	2971425	2720400	2649618	2405662	2117228	902737
14	2944946	2935978	2608636	2536935	2313061	2037318	820416
15	2918394	2874385	2543448	2403096	2187728	1943101	757484
16	2884539	2826559	2510472	2367278	2118385	1818681	684150
17	2857641	2742356	2406208	2241843	1966486	1716607	620368
18	2837963	2657552	2301753	2143597	1892621	1593519	557591
19	2830490	2617374	2237390	2023861	1736858	1511692	509242
20	2746710	2507688	2129011	1954257	1649878	1409292	460306
21	2687219	2433790	2066244	1811573	1515258	1290576	416120
22	2601844	2379419	1995165	1774013	1416449	1175981	376705
23	2591411	2301738	1923422	1656120	1289339	1100343	340929
24	2493928	2197819	1856615	1528304	1192552	1002876	315324
25	2420286	2143348	1767766	1450692	1090751	914225	293514
26	2351296	2050256	1666331	1323560	983730	848707	257609
27	2256073	1978252	1610924	1218451	909005	781303	249885
28	2208555	1913771	1549019	1152475	839003	713857	205778
29	2130508	1866158	1485180	1067767	745696	642048	198210
30	2036790	1783387	1409028	979406	685441	579929	185988
31	1984731	1725004	1336560	901929	634238	545480	168408
32	1898873	1638496	1248135	826618	586490	498175	149097
33	1815739	1602074	1185218	777166	539318	444942	137550
34	1758944	1535198	1110415	694075	491303	410974	128998
35	1698724	1456856	1073889	640275	458252	393768	118984
36	1597989	1400837	985664	592100	423791	350258	116617
37	1577522	1331798	937854	541615	383369	313551	107429
38	1497900	1279581	882389	501291	360312	284390	95877
39	1429658	1217176	825412	467753	325538	264675	92324
40	1365810	1165163	799255	431389	299162	246017	85533
41	1293853	1106094	714790	396637	284865	223367	75600
42	1194964	1059070	672640	366641	255695	195378	72369
43	1143408	1013720	640136	326683	239638	192745	70375
44	1068377	957570	595963	290874	224546	160851	63976
45	1016814	909813	535757	263591	206540	146371	56809

Normalized to Time=0 by (RLU/RLU0)

			Gallic Acid				
Time (min)	800	600	400	300	200	100	0
0	1	1	1	1	1	1	1
1	0.99818	0.999107	0.975366	1.002782	1.008499	0.983574	0.927382
2	0.985736	0.990506	0.970242	0.988797	0.983093	0.975677	0.869029
3	0.971833	0.973702	0.958382	0.984512	0.978232	0.952344	0.79246
4	0.972004	0.967771	0.950042	0.977561	0.969254	0.947866	0.73669
5	0.960883	0.963839	0.937534	0.976749	0.955964	0.909672	0.689264
6	0.964996	0.959978	0.937072	0.953879	0.96199	0.889972	0.62251
7	0.963179	0.957208	0.929185	0.960056	0.935679	0.860549	0.569598
8	0.955068	0.94311	0.923378	0.94954	0.904418	0.822958	0.51748
9	0.942363	0.933159	0.912355	0.925282	0.88244	0.79697	0.484976
10	0.926854	0.936574	0.892011	0.923787	0.865249	0.768777	0.439969
11	0.941873	0.938127	0.869266	0.885559	0.830561	0.730214	0.399287
12	0.925172	0.92407	0.850434	0.863154	0.788687	0.70109	0.370199
13	0.925464	0.921594	0.830519	0.836983	0.757894	0.662449	0.333554
14	0.918953	0.9106	0.796398	0.801388	0.728721	0.637446	0.303137
15	0.910667	0.891497	0.776497	0.75911	0.689235	0.607967	0.279884
16	0.900103	0.876664	0.766429	0.747795	0.667389	0.569038	0.252788
17	0.89171	0.850548	0.734598	0.708172	0.619533	0.5371	0.229221
18	0.885569	0.824246	0.702709	0.677137	0.596263	0.498588	0.206025
19	0.883237	0.811785	0.683059	0.639314	0.54719	0.472986	0.188161
20	0.857094	0.777765	0.649972	0.617327	0.519787	0.440946	0.170079
21	0.83853	0.754846	0.63081	0.572255	0.477376	0.403802	0.153753
22	0.81189	0.737982	0.60911	0.56039	0.446247	0.367947	0.13919
23	0.808634	0.713889	0.587207	0.523149	0.406201	0.344281	0.125971
24	0.778215	0.681659	0.566811	0.482773	0.375709	0.313785	0.11651
25	0.755236	0.664764	0.539686	0.458256	0.343637	0.286047	0.108451
26	0.733708	0.635892	0.508719	0.418097	0.30992	0.265548	0.095184
27	0.703994	0.613559	0.491804	0.384894	0.286378	0.244458	0.092331
28	0.689166	0.593561	0.472904	0.364053	0.264325	0.223355	0.076033
29	0.664812	0.578793	0.453415	0.337295	0.234929	0.200887	0.073237
30	0.635568	0.553122	0.430166	0.309383	0.215945	0.181451	0.068721
31	0.619323	0.535014	0.408042	0.284909	0.199814	0.170672	0.062225
32	0.592532	0.508183	0.381047	0.261119	0.184771	0.155871	0.05509
33	0.56659	0.496887	0.361839	0.245498	0.16991	0.139216	0.050824
34	0.548868	0.476145	0.339002	0.21925	0.154783	0.128588	0.047664
35	0.530077	0.451847	0.327851	0.202255	0.14437	0.123204	0.043964
36	0.498643	0.434473	0.300916	0.187037	0.133514	0.10959	0.043089
37	0.492256	0.41306	0.28632	0.17109	0.120779	0.098105	0.039694
38	0.467411	0.396865	0.269387	0.158352	0.113515	0.088981	0.035426
39	0.446116	0.37751	0.251992	0.147758	0.102559	0.082813	0.034113
40	0.426193	0.361378	0.244007	0.136271	0.09425	0.076975	0.031604
41	0.403739	0.343058	0.21822	0.125293	0.089746	0.069888	0.027934
42	0.372881	0.328473	0.205352	0.115818	0.080556	0.061131	0.02674
43	0.356794	0.314408	0.195429	0.103195	0.075497	0.060307	0.026003
44	0.333381	0.296993	0.181943	0.091884	0.070742	0.050328	0.023639
45	0.317291	0.282181	0.163563	0.083265	0.06507	0.045797	0.02099

Use normalized data to generate Area Under the Curve (AUC) values. AUC values can be calculated by a statistical program (such as GraphPad Prism) or by the following formula:

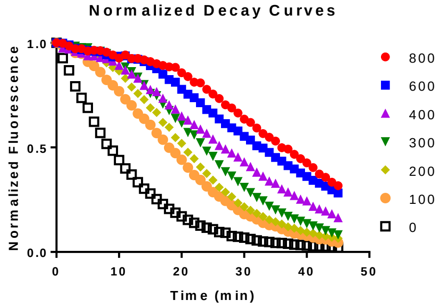

AUC= 0.5 + (F1/F0) + (F2/F0) + …+ 0.5*(F30/F0)
Where F0= normalized fluorescence at t=0

Net AUC is determined by subtracting the AUC for no compound addition from the other AUC values.

	800	600	400	300	200	100	0
AUC	33.4	31.3	26.9	24.6	21.8	19.5	11.5
Net AUC	21.9	19.8	15.4	13.1	10.3	8.1	0

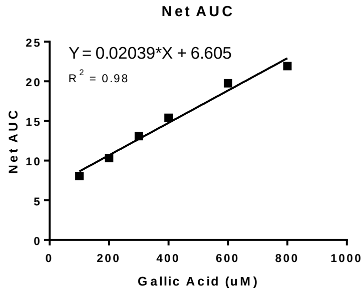

Data for unknowns may be expressed as
µM Gallic Acid Equivalents or µmole GA/gram.

APPENDIX A: Plate layout________________________________

	1	2	3	4	5	6	7	8	9	10	11	12
A												
B												
C												
D												
E												
F												
G												
H												

APPENDIX B: Protocol Flowchart

HORAC ASSAY

Make necessary test compound dilutions in Assay Buffer.

↓

Prior to assay, warm plate chamber to 37°C, prepare fluorescein working solution and gallic acid standards.

↓

Add 140 µl/well Working Fluorescein Solution to blank assay plate.

↓

Add 20 µl/well gallic acid standards and test samples to fluorescein containing wells and place in incubator at 37°C for 10 minutes.

↓

Prepare Radical Initiator solution. Add 20 µl/well of Radical Initiator working solution.

↓

Add 20 µl/well of Fenton Reagent Solution and place assay plate in plate reader. Begin kinetic fluorescence read.
Excitation= 485 nm; Emission=528 - 538 nm; (Cutoff=530 nm, if necessary)

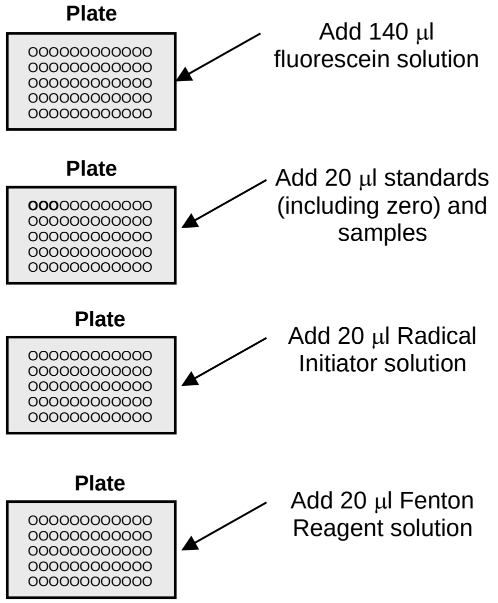

REFERENCES

1. J Agr and Food Chem. 50: 2772-2777, 2002.
2. Food Control, 21:518-523, 2010.

FREQUENTLY ASKED QUESTIONS

1. **Is it alright that my fluorescence values are lower than those in the sample data but still generate a good Gallic Acid standard curve?** Yes, the relative fluorescence values detected by the fluorimeter are based on the sensitivity of the instrument used. Our data was collected using a SpectrMax iD3 fluorimeter, other instruments vary in sensitivity and can give lower values. If the Gallic Acid standards still generate a robust standard curve, the assay is functioning appropriately.

2. **Should I dilute my sample for testing its AOX activity?** In order to accurately determine the AOX activity of your sample, the Net AUC value must fall on the Gallic Acid Net AUC standard curve. We recommend preparing several serial dilutions of your test sample using the AOX assay buffer to ensure that you generate usable Net AUC values.

Supplementary information 9

(A)

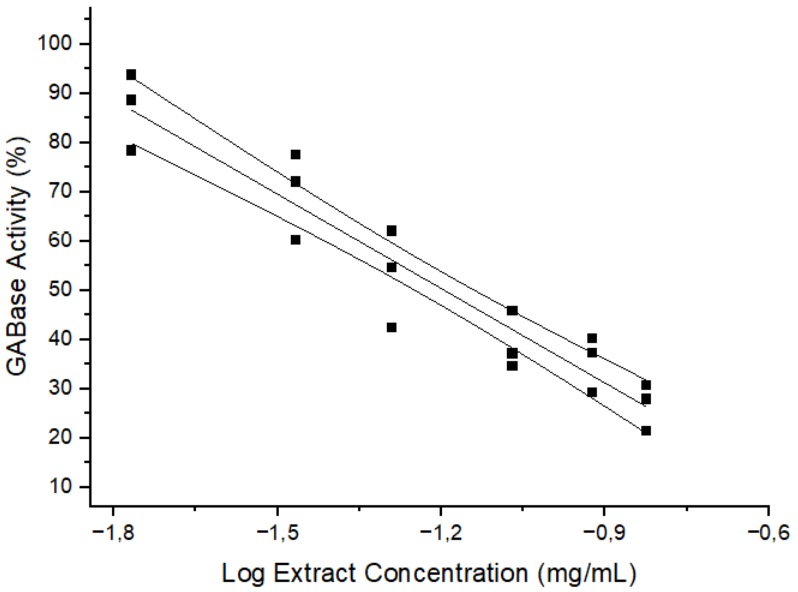

(B)

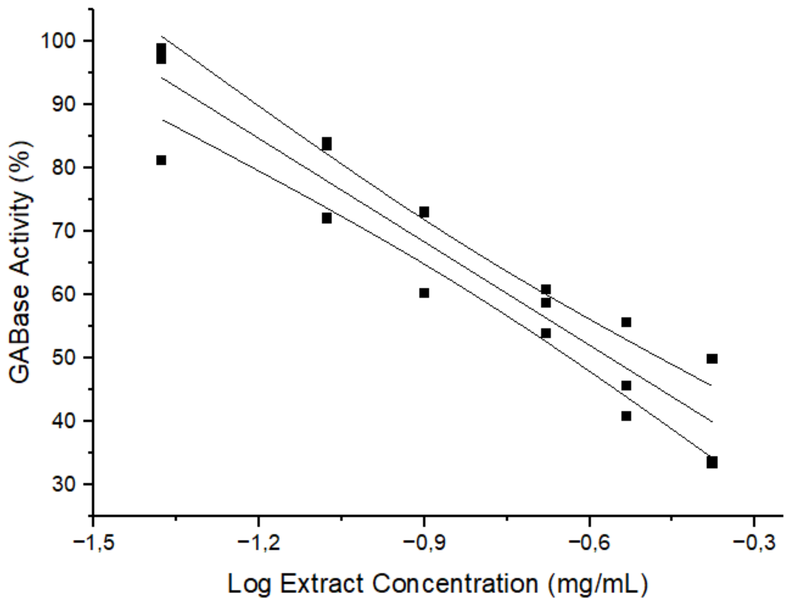

Linear regression analysis [IC_{50} (±95% CI)] of the *Melissa officinalis. L* (MO) extracts in *in vitro* GABA-transaminase (GABA-T) activity, (A) Relissa™ [IC_{50} = 0.064 mg/mL (0.056-0.072)] and (B) unformulated dry MO extract [IC_{50} = 0.27 mg/ml (0.23-0.33)]. GABA-T activity expressed as percent relative to control. Each concentration was tested in triplicate.

Supplementary information 10

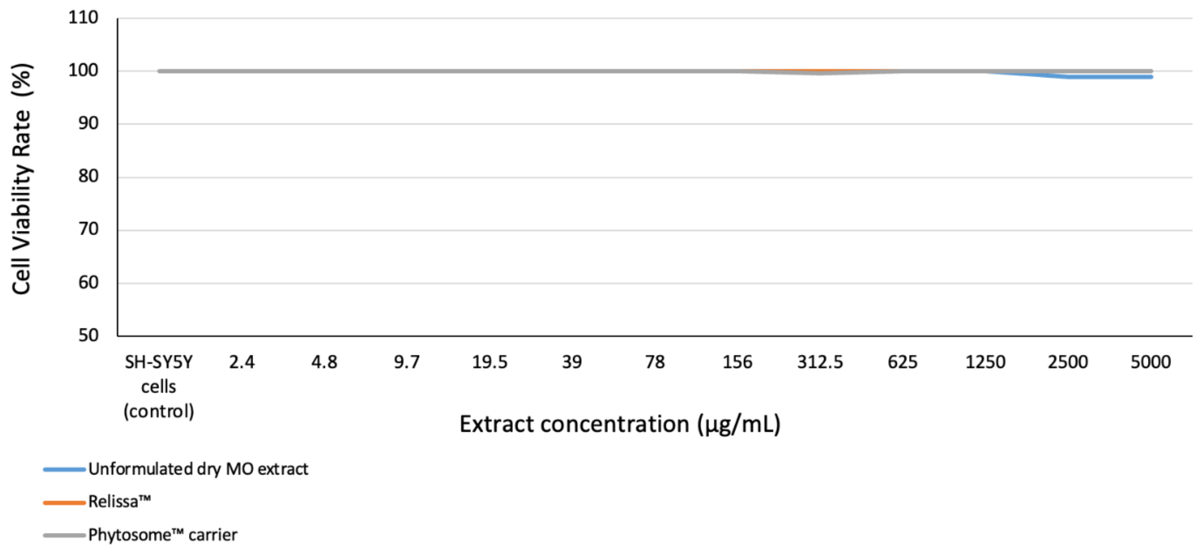

Results of SH-SY5Y cells MTT assay with *Melissa Officinalis. L* (MO) extracts
